# High-throughput screening for small-molecule stabilizers of misfolded glucocerebrosidase in Gaucher disease and Parkinson’s disease

**DOI:** 10.1073/pnas.2406009121

**Published:** 2024-10-10

**Authors:** Darian Williams, Logan M. Glasstetter, Tiffany T. Jong, Tiffany Chen, Abhijeet Kapoor, Sha Zhu, Yanping Zhu, Raul Calvo, Alexandra Gehrlein, Kimberly Wong, Andrew N. Hogan, David J. Vocadlo, Ravi Jagasia, Juan J. Marugan, Ellen Sidransky, Mark J. Henderson, Yu Chen

**Affiliations:** ^a^Division of Preclinical Innovation, National Center for Advancing Translational Sciences, NIH, Rockville, MD 20850; ^b^Molecular Neurogenetics Section, Medical Genetics Branch, National Human Genome Research Institute, NIH, Bethesda, MD 20892; ^c^Department of Chemistry, Simon Fraser University, Burnaby, BC V5A 1S6, Canada; ^d^Department of Molecular Biology and Biochemistry, Simon Fraser University, Burnaby, BC V5A 1S6, Canada; ^e^Roche Pharma Research and Early Development, Neuroscience and Rare Diseases Discovery and Translational Area, Roche Innovation Center Basel, 4070 Basel, Switzerland

**Keywords:** Gaucher disease, Parkinson’s disease, high-throughput screening, glucocerebrosidase, pharmacological chaperone

## Abstract

Glucocerebrosidase (GCase) is implicated in both a rare, monogenic disorder (Gaucher disease, GD) and a common, multifactorial condition (Parkinson’s disease, PD); hence, it is an urgent therapeutic target. To identify correctors of severe protein misfolding and trafficking obstruction manifested by the pathogenic L444P-variant of GCase, we developed a suite of quantitative, high-throughput, cell-based assays. First, we labeled GCase with a small proluminescent HiBiT peptide reporter tag, enabling quantitation of protein stabilization in cells while faithfully maintaining target biology. TALEN-based gene editing allowed for stable integration of a single HiBiT-*GBA1* transgene into an intragenic safe-harbor locus in *GBA1*-knockout H4 (neuroglioma) cells. This GD cell model was amenable to lead discovery via titration-based quantitative high-throughput screening and lead optimization via structure–activity relationships. A primary screen of 10,779 compounds from the NCATS bioactive collections identified 140 stabilizers of HiBiT-GCase-L444P, including both pharmacological chaperones (ambroxol and noninhibitory chaperone NCGC326) and proteostasis regulators (panobinostat, trans-ISRIB, and pladienolide B). Two complementary high-content imaging-based assays were deployed to triage hits: The fluorescence-quenched substrate LysoFix-GBA captured functional lysosomal GCase activity, while an immunofluorescence assay featuring antibody hGCase-1/23 directly visualized GCase lysosomal translocation. NCGC326 was active in both secondary assays and completely reversed pathological glucosylsphingosine accumulation. Finally, we tested the concept of combination therapy by demonstrating synergistic actions of NCGC326 with proteostasis regulators in enhancing GCase-L444P levels. Looking forward, these physiologically relevant assays can facilitate the identification, pharmacological validation, and medicinal chemistry optimization of small molecules targeting GCase, ultimately leading to a viable therapeutic for GD and PD.

## Significance

Gaucher disease, the inherited deficiency of glucocerebrosidase, is caused by biallelic loss-of-function mutations in the gene *GBA1,* which is also the most frequent genetic risk factor for Parkinson’s disease. While the development of small-molecule stabilizers of glucocerebrosidase is being considered for both disorders, discovery and optimization of lead compounds is limited by the lack of robust cell-based assays amenable to high-throughput screening format. We developed a comprehensive assay pipeline for preclinical discovery of glucocerebrosidase modulators and began by screening libraries enriched with bioactive compounds with known mechanisms of action. The screen identified small molecules with established relevance to glucocerebrosidase, provided an atlas of potential new molecular targets regulating the *GBA1* pathway, and produced a set of promising potential therapeutics.

*GBA1* encodes β-glucocerebrosidase (GCase), a lysosomal enzyme that hydrolyzes glycosphingolipid substrates, including glucosylceramide (GluCer) and glucosylsphingosine (GluSph). Biallelic loss-of-function mutations in *GBA1* cause Gaucher disease (GD), the most common lysosomal storage disorder (LSD). In addition, *GBA1* mutations serve as the most frequent genetic risk factor for synucleinopathies, such as Parkinson’s disease (PD) (1) and dementia with Lewy bodies (DLB) (2). This genetic association is strongly supported by preclinical (3, 4) and clinical (5, 6) evidence. GCase is thus firmly positioned as a well-validated therapeutic target for modification of both a rare, monogenic disorder and a common, multifactorial disease, motivating translational efforts aimed at its enhancement.

The current standard of care for GD is enzyme replacement therapy (ERT), whereby recombinant, mannose-terminated GCase is delivered to lipid-laden macrophages via biweekly intravenous infusions (7). While ERT offers dramatic reversal of the peripheral symptoms of the disease, such as hepatosplenomegaly and hematologic abnormalities (8), it is rapidly cleared from the blood and does not penetrate the blood–brain barrier (BBB) (9). Therefore, it has no effect on the neurologic manifestations seen in type 2 (acute neuronopathic) or type 3 (subacute neuronopathic) GD (10), and it does not prevent or modify the progression of parkinsonism in patients with GD (11). In addition, ERT is expensive and inconvenient. Since GD is a multisystem disorder, an oral small-molecule therapy is highly desirable. Unfortunately, substrate reduction therapy, which inhibits the synthesis of GluCer, does not slow the progression of *GBA1*-PD (12), and it fails to target GCase dysfunction upstream of the lipid pathology. Thus, there is an urgent unmet need for a BBB-penetrant, orally available small-molecule drug that enhances GCase levels and functions in the brain, either by rescuing mutant forms of the protein or, in *GBA1* mutation carriers, by enhancing remaining GCase-WT to compensate for haploinsufficiency. We envision that such a pharmaceutical would be widely applicable, as a first-line therapy for neuronopathic GD, as a prospective prophylactic agent against *GBA1*-PD, and, perhaps most broadly impactful, as a potential disease-modifying therapy for sporadic PD (3, 13–15).

GD is fundamentally a loss-of-function (LOF) disease. Several underlying mechanisms exist, including null mutations (e.g., c.84GG and IVS2+1), loss of catalytic activity (16), and disrupted interactions with binding partners (17), but many disease-causing variants encode a misfolded protein that is not properly trafficked (18). Missense mutations that disrupt protein folding can lead to premature endoplasmic reticulum-associated degradation (ERAD) of GCase (18–20), which, if it were productively trafficked to the lysosome, might express some residual catalytic activity (21–23). The ERAD process involves recognition and retention of aberrant GCase in the ER, followed by its retrotranslocation into the cytosol and subsequent degradation by the ubiquitin–proteasome system (24). Importantly, different mutant GCase variants present variable degrees of ERAD, underlying some of the clinical heterogeneity encountered in GD (25). The two most common *GBA1* mutations are N370S (p.N409S), which represents 70% of the mutant alleles in the Ashkenazi Jewish population and is exclusively associated with type 1 GD, and L444P (p.L483P), a severe, pan-ethnic mutation within the hydrophobic core of the immunoglobulin-like domain of GCase that leads to substantial protein instability and is often encountered in neuronopathic (types 2 and 3) GD (26).

Attempts to salvage misfolded GCase from the ERAD pathway using small molecules have focused on two potentially synergistic therapeutic paradigms (27): a generic biological arm involving reprogramming of the protein homeostasis network with proteostasis regulators (PRs) (28–31) and a tailored chemical arm consisting of pharmacological chaperones (PCs) that stabilize the native state of the enzyme (32) or mobilize a trafficking-competent conformation (33–36) through direct binding. The PC approach initially focused on the identification of active-site inhibitors; these are primarily substrate-mimetic compounds (iminosugars), such as isofagomine (37, 38) and *N*-(*n*-nonyl)deoxynojirimycin (32), but some unique chemical scaffolds were also identified via high-throughput screening efforts (39–41). These compounds must be utilized at subinhibitory concentration, suffer from a narrow therapeutic window between chaperoning and inhibitory behavior, and exhibit poor selectivity against related hydrolases (e.g., α-glucosidase, α-galactosidase) (42). The most promising PC in the inhibitor class is ambroxol, an expectorant that was isolated in a screen of 1,040 approved drugs based on a thermal denaturation assay with wild-type recombinant GCase (43). Ambroxol exhibits pH-dependent chaperone behavior (43), is BBB permeable and well tolerated (13, 44), and showed favorable results in small pilot studies for neuronopathic GD (44, 45), with clinical trials in progress for PD (NCT05778617) and DLB (NCT04588285). However, ambroxol is an inhibitory chaperone (46), which may place a ceiling on its therapeutic potential (13).

Noninhibitory, allosteric site-directed PCs have emerged as a favorable therapeutic strategy, with a wider therapeutic window than active-site inhibitors. The first members of this class were discovered through a quantitative high-throughput screening (qHTS) campaign (47), which utilized Gaucher spleen homogenate as a source of mutant (N370S) GCase (48) and leveraged pure activation toward cleavage of a fluorogenic substrate as a surrogate for binding and chaperoning potential (49). Two hits from this screen of 250,000 compounds were advanced by medicinal chemistry to yield lead compounds NCGC607 (50) and NCGC758 (49, 51), which have distinct chemotypes. In *GBA1*-PD patient-derived macrophages, these PCs increased GCase protein levels, enhanced GCase activity, and reduced GluCer and GluSph substrate accumulation; in patient-derived dopaminergic neurons, they also lowered α-synuclein levels (50–52).

While biochemical assays (48) enable identification of GCase activators, they are not suitable for directly identifying small molecules that stabilize GCase and promote its translocation to the lysosome, either through chemical (PCs) or biological (PRs) mechanisms. Furthermore, the binding of small molecules to GCase, modulation of its enzymatic activity, and promotion of its folding do not always correlate, so previously described screening methods are not adequate to drive medicinal chemistry efforts toward optimization of PCs. Ideally, lead compounds should be discovered and optimized in a cellular or phenotypically relevant context. In this work, we solve these issues by successfully labeling GCase with a small (1.3 kDa) HiBiT tag (53), which, unlike larger reporters, does not perturb the trafficking or function of the enzyme. HiBiT undergoes high-affinity complementation with exogenous LgBiT to reconstitute an active luciferase. As a primary assay, it provides a bioluminescent readout of GCase protein levels in cells that is amenable to both lead discovery (54, 55) and medicinal chemistry optimization via structure–activity relationships. To confirm that hits identified from the HiBiT primary screen enhance GCase lysosomal activity and trafficking, we implemented two high-content imaging-based secondary assays, leveraging the fluorescence-quenched substrate LysoFix-GBA (56) and the newly described GCase antibody hGCase-1/23 (57). Finally, as an endpoint, we evaluated the reversal of glycosphingolipid substrate accumulation (58). We chose to first deploy this screening pipeline on pharmacologically active, mechanistically annotated compound libraries (59, 60), with a focus on drug repurposing (54). The best PRs derived from this screen were then combined with a newly identified analog of an in-house, noninhibitory PC, to establish a potent and efficacious, synergistic coformulation (27). Future work will deploy the pipeline on a diversity library to discover additional PCs and PRs.

## Results

### The HiBiT Peptide Reporter Tag Preserves Trafficking and Function of Labeled GCase Variants, Providing a Cell-Based Platform for qHTS.

**Fig. 1.**
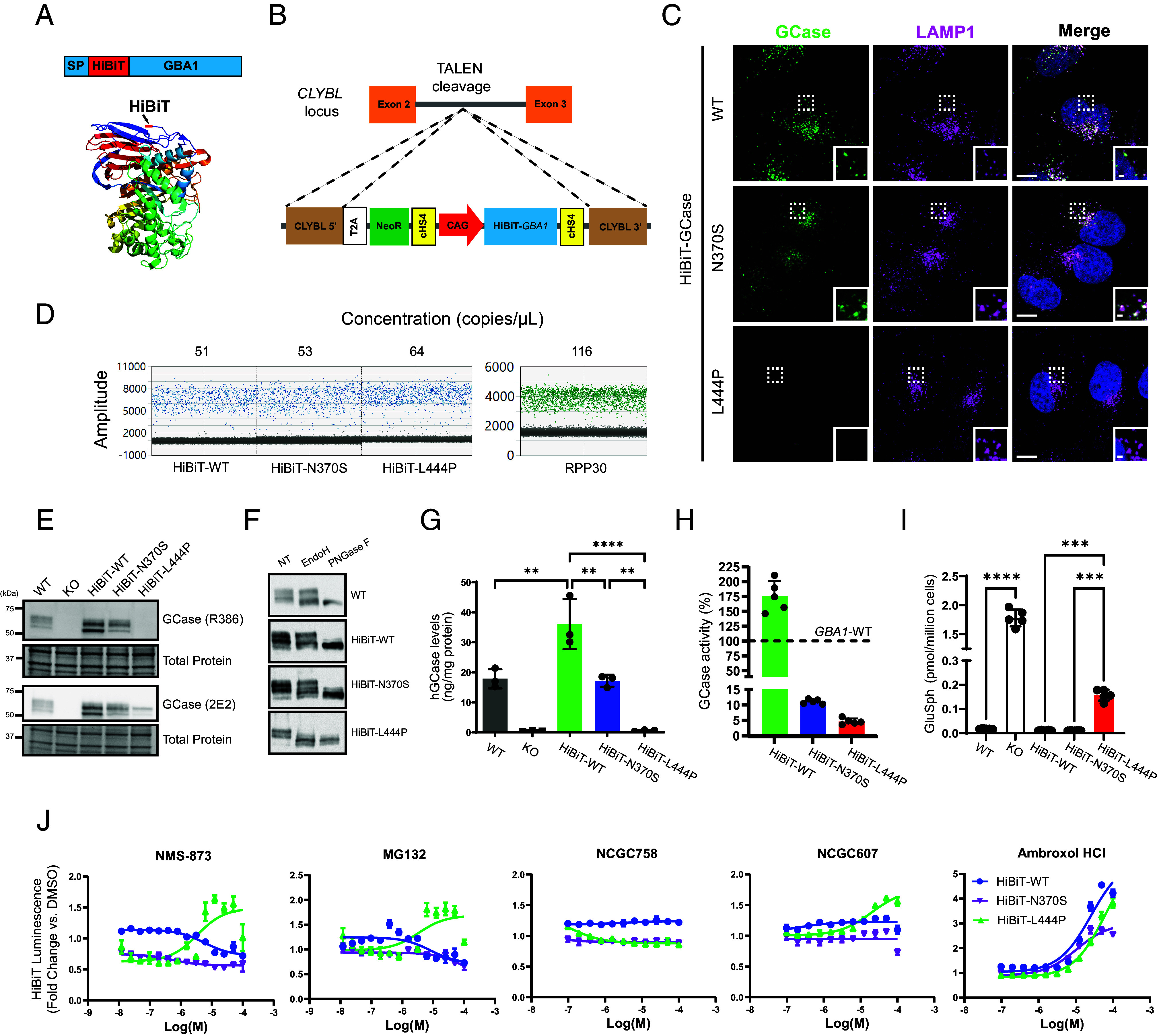
HiBiT-tagged GCase retains normal trafficking and function, enabling a high-throughput screening assay measuring cellular GCase levels. (*A*) GCase was labeled with a small (1.3 kDa), proluminescent, N-terminal HiBiT peptide tag immediately following the signal peptide (SP) sequence. (*B*) HiBiT-GCase reporters (WT, N370S, or L444P) were engineered into the human Citrate Lyase Beta-Like (*CLYBL)* intragenic safe-harbor locus within a *GBA1*-KO H4 cell background using TALEN-enhanced integrative gene transfer. (*C*) Colocalization of GCase (green) with lysosomal marker LAMP1 (magenta) was determined by immunofluorescent staining. (Scale bar, 10 µm; *Inset* scale bar, 1 µm.) (*D*) The copy number of the stably integrated transgene was confirmed to be 1 across all three HiBiT-GCase lines via Droplet Digital PCR (ddPCR). The HiBiT-GCase H4 lines featured ~60 copies/μL of the HiBiT-*GBA1* transgene, as compared with ~120 copies/μL of the reference gene RPP30, which has a known copy number of 2 in the *GBA1*-WT H4 cell line. (*E*) GCase protein level was measured by western blot in *GBA1*-WT (unedited), *GBA1*-KO, and HiBiT-GCase (WT, N370S, or L444P) H4 cell lines using anti-GCase (R386 and 2E2) antibodies, with total protein as the loading control. (*F*) Glycosidase sensitivity analysis indicates that HiBiT-GCase-L444P is entirely retained in the ER. The Endo H-sensitive fraction (lower band) on the blot contains immature, ER-retained GCase, while the Endo H-resistant fraction (top band) contains maturely glycosylated, post-ER-localized GCase. Both fractions are responsive to PNGase F treatment. NT: nontreated. (*G*) GCase protein levels were quantitated by AlphaLISA (Amplified Luminescent Proximity Homogeneous Assay) utilizing a sandwich configuration of two monoclonal antibodies recognizing nonoverlapping epitopes, hGCase-1/23 (which was biotinylated and associated with a streptavidin-coated donor bead) and hGCase-1/17 (which was directly conjugated to an acceptor bead). (Error bars: SEM [*n* = 3 biological replicates]). (*H*) GCase activity was measured in cell lysates using the fluorogenic substrate 4-methylumbelliferyl-β-D-glucopyranoside. Relative GCase activity was calculated by adjusting for protein concentration, correcting for *GBA1*-KO H4 cell background, and normalizing to *GBA1*-WT signal. (Error bars: SD [*n* = 5 biological replicates]). (*I*) Levels of glucosylsphingosine (GluSph) in H4 cell pellets were quantified by positive ion electrospray LC–MS/MS in multiple reaction-monitoring mode, using deuterated compounds as internal standards. (Error bars: SD [*n* = 5 biological replicates]). (*J*) Pilot testing of the HiBiT-GCase assay was performed in ultrahigh-throughput 1536-well plate format. Cells were treated with known-active ERAD modulators (NMS-873, p97 inhibitor; MG132, proteasome inhibitor) or GCase stabilizers (ambroxol, NCGC758, or NCGC607) for 24 h, followed by measurement of HiBiT-GCase luminescence. For each respective cell line, data are represented as fold change in luminescence (RLU) in compound-treated versus DMSO-treated cells. (Error bars: SEM [*n* = 3 to 6]). Dose–response curves were fit using log(agonist) vs. response (three parameters). *P-value ≤ 0.05; **P-value ≤ 0.01; ***P-value ≤ 0.001; ****P-value ≤ 0.0001.

The HiBiT reporter is an 11-amino acid peptide tag suitable for luminescence-based qHTS. To explore the feasibility of tagging human GCase at its N terminus with HiBiT (Fig. 1*A*), prior to generating stable cell lines, we transiently transfected cells with constructs encoding a transgene containing the *GBA1* signal peptide, the HiBiT peptide tag, and a Gly/Ser linker upstream of *GBA1*. The transgene was expressed in a *GBA1*-KO H4 (human neuroglioma) cell line, and localization of the reporter protein was assessed using a monoclonal human GCase antibody, hGCase-1/23 (57) (*SI Appendix*, Fig. S1). When moderately expressed, HiBiT-GCase-WT showed specific lysosomal localization comparable to endogenous GCase and transfected untagged GCase, suggesting that the HiBiT tag did not interfere with GCase trafficking. However, higher levels of untagged GCase led to ER accumulation without clear lysosomal localization, indicating that GCase trafficking is perturbed by overexpression. To address this, HiBiT-GCase-WT expression was constrained by integrating the transgene cassette into a safe-harbor site within an intron of the Citrate Lyase Beta-Like (*CLYBL*) gene using transcription activator-like effector nucleases (TALENs) (61, 62) in the *GBA1*-KO H4 cell line (Fig. 1*B*). This integrative gene transfer method allows efficient knock-in of the large transgene, with minimal impact on local and global gene expression (61). Sustained expression of the transgene is driven by a strong CAG promoter. A *GBA1*-KO H4 clone with a single copy of HiBiT-*GBA1*-WT was identified via Droplet Digital PCR (ddPCR) and used for subsequent experiments (Fig. 1*D*). The expression level (Fig. 1 *E* and *G*) and activity (Fig. 1*H*) of HiBiT-GCase-WT were moderately higher than those of endogenous GCase. Both proteins showed similar lysosomal localization (Fig. 1*C* and *SI Appendix*, Fig. S2 *A* and *B*) and glycosylation status (Fig. 1*F*). Colocalization of GCase-WT with its trafficking receptor, LIMP-2, was not affected by the HiBiT tag (*SI Appendix*, Fig. S2 *C* and *D*). Moreover, lysosomal targeting of HiBiT-GCase-WT protein was dependent on the presence of LIMP-2 in the H4 cells (*SI Appendix*, Fig. S3*B*), as observed for endogenous GCase (63) (*SI Appendix*, Fig. S3*A*), suggesting that the small HiBiT tag did not disrupt this protein–protein interaction. Additionally, HiBiT-GCase-WT successfully reduced accumulated GluSph (Fig. 1*I*) and GluCer (*SI Appendix*, Fig. S4) in the *GBA1*-KO H4 cell line, behaving akin to endogenous GCase and confirming functional activity of the reporter protein.

HiBiT-GCase-N370S and -L444P cell lines were also generated using the same strategy in *GBA1*-KO H4 cells. HiBiT-GCase-N370S exhibited lysosomal localization similar to HiBiT-GCase-WT, whereas HiBiT-GCase-L444P had no staining, likely due to severe misfolding (Fig. 1*C* and *SI Appendix*, Fig. S2 *A* and *B*). Furthermore, HiBiT-GCase-N370S, unlike HiBiT-GCase-L444P, had near-normal colocalization with LIMP-2 (*SI Appendix*, Fig. S2 *C* and *D*). The expression level of HiBiT-GCase-L444P, detected by western blot, was notably lower than HiBiT-GCase-WT and HiBiT-GCase-N370S levels (Fig. 1*E*). GCase protein levels in the HiBiT-GCase-N370S and -L444P lines were quantified by a bead-based immunoassay—Amplified Luminescent Proximity Homogeneous Assay (AlphaLISA)—as 48% and 2% of HiBiT-GCase-WT levels, respectively (Fig. 1*G*). Endo H treatment revealed sensitivity in a modest fraction of HiBiT-GCase-N370S and a large fraction of HiBiT-GCase-L444P, indicating the predominantly immature glycosylation status of the latter, resulting from increased misfolding and ER retention (Fig. 1*F*). GCase activity was measured in lysates of HiBiT-GCase-N370S and HiBiT-GCase-L444P H4 cells as 11% and 4.6%, respectively, relative to the endogenous level in *GBA1*-WT H4 cells (Fig. 1*H*). Consistent with the threshold theory of residual enzymatic activity proposed by Conzelmann and Sandhoff to describe other LSDs (64), it has been demonstrated in cellular models of GD that GCase activity can be reduced to ~11 to 15% of the normal control level before increased storage of substrates occurs (65). While HiBiT-GCase-N370S and -L444P both successfully eliminated accumulated GluCer in the *GBA1*-KO H4 cell line (*SI Appendix*, Fig. S4), HiBiT-GCase-L444P only partially decreased the accumulation of GluSph, unlike HiBiT-GCase-WT and -N370S (Fig. 1*I*), suggesting that HiBiT-GCase-N370S, but not HiBiT-GCase-L444P, exceeds the critical threshold for GluSph accumulation.

### HiBiT-GCase-L444P Is Responsive to PCs and PRs in a Quantitative High-Throughput Luminescence Assay.

To demonstrate the utility of the HiBiT-GCase H4 lines as a drug discovery tool, we miniaturized the HiBiT assay to ultrahigh-throughput (1,536-well plate) format and tested a set of known-active GCase stabilizers after 24 h treatment. MG132 is a proteasome inhibitor that also up-regulates ER folding capacity through unfolded protein response (UPR) activation (27). NMS-873 is an inhibitor of the valosin-containing protein (VCP) p97, which hydrolyzes ATP to extract misfolded proteins from the ER into the cytosol, thereby enabling ERAD via the proteasome. Both PRs selectively stabilized HiBiT-GCase-L444P with a dose-dependent response, indicating its constitutive loss through ERAD (25) (Fig. 1*J*). The response to noninhibitory, allosteric-site-directed PC NCGC607 (50) displayed a similar selectivity for HiBiT-GCase-L444P, whereas the inhibitory, active-site-directed PC ambroxol (43, 66) increased HiBiT-GCase levels in all three lines. Notably, noninhibitory PC NCGC758 was inactive in all three lines under these experimental conditions. Overall, these results demonstrate the ability of the HiBiT-GCase method to detect relevant small-molecule stabilizers of GCase, including both PRs and PCs. A treatment period of 24 h was sufficient to detect changes in the steady-state levels of GCase; biogenesis and maturation of native GCase occur within this timeframe (67), and the half-life of L444P-mutant GCase is substantially shorter (68). While a fraction of GCase folding intermediates will be targeted for ERAD regardless of mutation status, ERAD plays a more direct, rate-limiting role in the processing of the severely misfolded L444P variant, as compared with WT or N370S (25, 67, 69, 70). Under the given assay conditions, the effect of PRs on GCase stabilization was highest in the L444P reporter line, so this variant was the focus of subsequent high-throughput screening.

### Identification of Novel Compounds and Mechanistic Classes that Promote Stabilization of HiBiT-GCase-L444P Protein Levels.

**Fig. 2.**
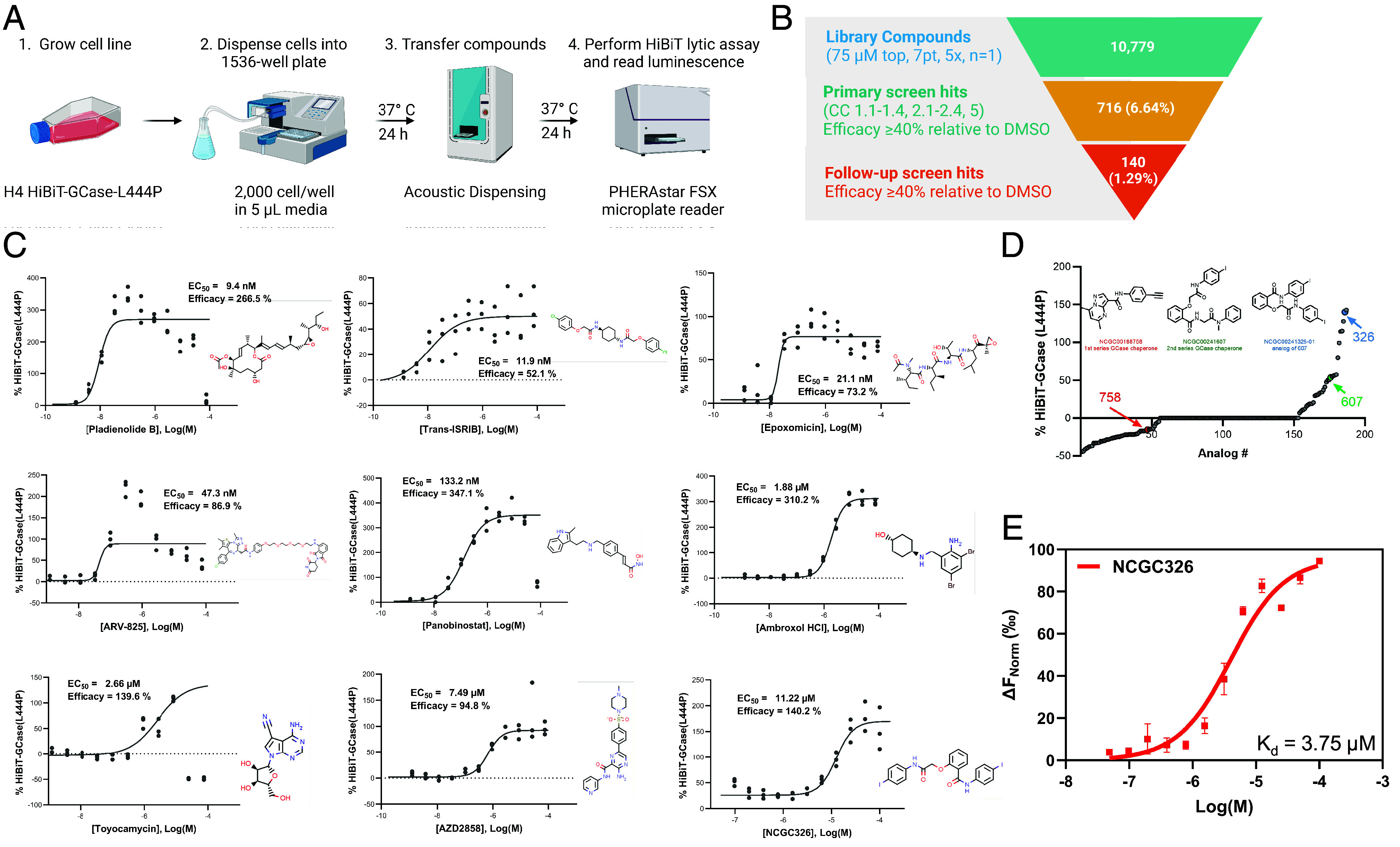
Quantitative high-throughput screening of mechanistically annotated small-molecule libraries for GCase-L444P stabilizers. (*A*) Schematic of high-throughput screening methodology using H4 HiBiT-GCase-L444P reporter cell line. (*B*) In the primary screen, 10,779 compounds, including the NCATS Pharmaceutical Collection (NPC), NCATS Pharmacologically Active Chemical Toolbox (NPACT), and Helping to End Addiction Long-term (HEAL) chemical libraries, as well as analogs of the noninhibitory chaperone chemotypes NCGC607 and NCGC758, were evaluated at concentrations most commonly ranging from 5 nM to 75 μM in a 7-point, 5× dilution series using a single replicate. Compounds were then triaged based on curve class (CC) and efficacy: Those with CC of 1.1 to 1.4, 2.1 to 2.4, or 5 and efficacy ≥40% were considered primary screen hits. These 716 compounds were retested (NCGC326: 100 nM to 100 μM, 11-point, 2× dilution; others: 1 nM to 75 μM, 11-point, 3× dilution) with *n* = 3 replicates and categorized as final hits if they met a cutoff of efficacy ≥40%, regardless of curve class, resulting in 140 confirmed hits. (*C*) Top representative follow-up screen hits from each mechanistic cluster were selected based on their potency and efficacy in stabilizing GCase-L444P levels. Response values were normalized to intraplate DMSO-treated controls, such that 100% efficacy reflects a doubling of GCase levels. *n* = 3. (*D*) Dose–response profiles for 187 analogs of chaperones NCGC758 and NCGC607 revealed 11 compounds, including NCGC326 (blue), with greater efficacy than NCGC607 (green). NCGC758 (red) was inactive under the screening conditions. (*E*) Target engagement studies with microscale thermophoresis revealed that NCGC326 binds recombinant GCase-WT with a dissociation constant (K_d_) of 3.75 μM in 50 mM sodium citrate buffer (pH 5.5). *n* = 2.

Using the HiBiT-GCase-L444P H4 posttranslational reporter cell line (Fig. 2*A*), we performed qHTS on a collection of 10,779 compounds, including three annotated small-molecule libraries: NPC (NCATS Pharmaceutical Collection) (59), NPACT (NCATS Pharmacologically Active Chemical Toolbox) (60), and HEAL (Helping to End Addiction Long-term; https://ncats.nih.gov/research/research-activities/heal/expertise/library). We also screened analogs of two noninhibitory PC chemotypes, NCGC607 and NCGC758, based on a SMILES similarity search within all internal NCATS libraries using a similarity cutoff of 80%, which retrieved 187 compounds (NCGC607 analogs: 91 compounds; NCGC758 analogs: 96 compounds). All 10,779 compounds were screened at 7 concentrations ranging most commonly from 5 nM to 75 µM (Fig. 2*B*). From the primary screen, 716 compounds (6.6%) met the selection criteria as hits and were cherrypicked for expanded dose–response testing, along with CellTiter-Glo cytotoxicity testing (*SI Appendix*, Fig. S5). Of the cherrypicked hits, 140 were confirmed in follow-up testing: 75 final hits were derived from NPACT, 43 from HEAL, 6 from NPC, and 16 from the set of chaperone analogs (Fig. 2*B* and *SI Appendix*, Fig. S6*A* and Table S1). The final hits represented a few salient mechanisms of action (Fig. 2*C*), and analysis of their molecular targets (*SI Appendix*, Fig. S6*B*) revealed several enriched pathways (*SI Appendix*, Table S2). Most hits were epigenetic modulators, including histone deacetylase (HDAC) inhibitors (42/140) like panobinostat, and BET bromodomain protein inhibitors or degradation inducers (5/140) like ARV-825. HDAC inhibitors are known GCase-L444P stabilizers (71, 72). In addition, known actives NMS-873 (*SI Appendix*, Fig. S7) and ambroxol HCl (Fig. 2*C*) were detected as final hits in the screen, providing validation of the HiBiT-GCase-L444P H4 reporter cell line as a powerful qHTS tool that identifies relevant chemical matter.

Other mechanistic classes identified in the primary screen included two proteasome inhibitors (epoxomicin and ixazomib) and five glycogen synthase kinase 3 (GSK-3) inhibitors like AZD2858 (Fig. 2*C*). Of note, the most potent hit identified in the primary screen was pladienolide B (EC_50_ = 9.4 nM), a splicing factor SF3B1 modulator (Fig. 2*C*). The third-most potent hit was trans-ISRIB (Fig. 2*C*), a known PR acting through inhibition of the PERK-mediated UPR pathway, whereby it releases the brake on translation (73); its potential therapeutic utility for GD or *GBA1*-PD remains to be determined. Toyocamycin is another hit acting through UPR inhibition; specifically, it blocks activation of the IRE1α-XBP1 arm (74). The most efficacious hit was an HDAC inhibitor, vorinostat (SAHA), with an efficacy of 658% relative to the DMSO control (*SI Appendix*, Table S1 and Fig. S7). The screen of analogs of PCs NCGC607 and NCGC758 (Fig. 2*D*) captured thirteen active compounds related to the NCGC607 (salicylic acid derivative) scaffold, including NCGC607 itself, and three active compounds with structural similarity to the NCGC758 (pyrazolopyrimidine) scaffold, including the investigational drug LTI-291/BIA 28-6156, which is currently being evaluated in a clinical trial for *GBA1*-PD (*SI Appendix*, Figs. S6*A* and S7) (75, 76). None of the PC analogs showed improved potency relative to the active parent compound NCGC607 (EC_50_ > 10 μM for all compounds). Nonetheless, the screen identified NCGC326 as a novel analog of NCGC607 with ~3-fold increased efficacy (140% vs. 54%) and similar potency (Fig. 2*C*). Moreover, microscale thermophoresis (49) confirmed binding of NCGC326 to recombinant GCase-WT (K_d_ = 3.75 μM), reaffirming its status as a PC (Fig. 2*E*). Finally, selected final hits underwent a counterscreen to rule out interference with the reconstituted luciferase enzyme during the HiBiT-GCase-L444P assay in H4 cells (*SI Appendix*, Fig. S8). Representative hits from each mechanistic cluster, along with the newly discovered PC NCGC326, were subsequently analyzed in orthogonal secondary assays.

### Orthogonal High-Content Screening Assays Enable Triage of Hits from the Primary Screen.

To enable triage of hits from the primary screen, we next sought to develop high-content imaging-based secondary assays to characterize the effect of small molecules on lysosomal translocation of an enzymatically active GCase. Small-molecule regulators of GCase-L444P can increase cellular protein levels without enhancing lysosomal translocation and enzymatic activity of the mutant protein. For example, proteasome inhibitors like bortezomib may not relinquish persistent ER-retention of the misfolded protein, while active-site-directed PCs like isofagomine (and even ambroxol) have an inhibitory effect on GCase lysosomal activity at high concentrations (μM) (46). Therefore, compounds which can increase total levels of GCase-L444P while simultaneously increasing lysosomal translocation and functional activity offer the greatest therapeutic potential. To identify such compounds, we implemented two complementary high-content screening (HCS) assays (77, 78). First, a *GBA1*-specific, fluorescence-quenched substrate, LysoFix-GBA, was used to directly evaluate GCase function within lysosomes (56). This sensitive lysosomal GCase activity probe is fixable, lysosomotropic (to minimize diffusive signal loss), and red-shifted (to reduce autofluorescence background) (*SI Appendix*, Fig. S9*A*) (56). Second, folding and translocation of GCase were assessed in an immunofluorescence assay using a new GCase antibody that only recognizes the properly folded, lysosomal fraction of GCase-L444P, but not the misfolded, ER-retained portion of the protein (57). Overall, these secondary assays provide complementary evidence and allow for triage of hits with the most desirable activity. The combination of the two orthogonal assays also enables deconvolution of allosteric-site-directed GCase enhancers functioning as pure enzyme activators from those behaving as bona fide PCs driving lysosomal translocation of the protein (49).

### LysoFix-GBA, a Fluorescence-Quenched Substrate, Provides Direct Visualization of GCase Function Within Lysosomes and Enables High-Content Validation of Small-Molecule GCase Enhancers.

To implement the LysoFix-GBA assay in high-throughput 384-well microplate format, we first aimed to identify the optimal concentration of the substrate for use in HiBiT-GCase-L444P H4 cells. Automated high-content analysis of subcellular structures provided a readout of integrated LysoFix-GBA spot intensity per cell. LysoFix-GBA demonstrated lysosome-specific activity at concentrations greater than 2.5 μM, which was inhibited by 24 h pretreatment with the GCase-selective inhibitor AT3375 (*SI Appendix*, Fig. S9 *B* and *C*). At a LysoFix-GBA concentration of 5 μM, HiBiT-GCase-L444P signal was ~15% of HiBiT-GCase-WT signal, and minimal background was detected in the *GBA1*-KO H4 line (*SI Appendix*, Fig. S9 *D* and *E*), providing an adequate dynamic range to interrogate modulation of GCase-L444P lysosomal activity. To further characterize the assay, we examined the effects of control compounds NMS-873, bortezomib, isofagomine, NCGC607, and NCGC758 on LysoFix-GBA signal in the HiBiT-GCase-L444P line (*SI Appendix*, Fig. S9*F*). Isofagomine showed an inhibitory LysoFix-GBA response, as expected for an active-site-directed PC; the response to bortezomib was also negative. ERAD modulator NMS-873 was inactive in the LysoFix-GBA assay, despite activity in the HiBiT assay. Allosteric-site-directed PCs NCGC607 and NCGC758 were both inactive in the LysoFix-GBA assay. Prior studies indicate that NCGC607 and NCGC758 are effective PCs of GCase in different cellular models—macrophages or dopaminergic neurons derived from patients with GD—and given a longer duration of treatment (6 to 21 d) (50, 51). These findings motivate utilization of the LysoFix-GBA assay as an orthogonal approach to filter hits from the HiBiT assay, allowing for selection of compounds that increase both GCase-L444P levels and lysosomal activity.

**Fig. 3.**
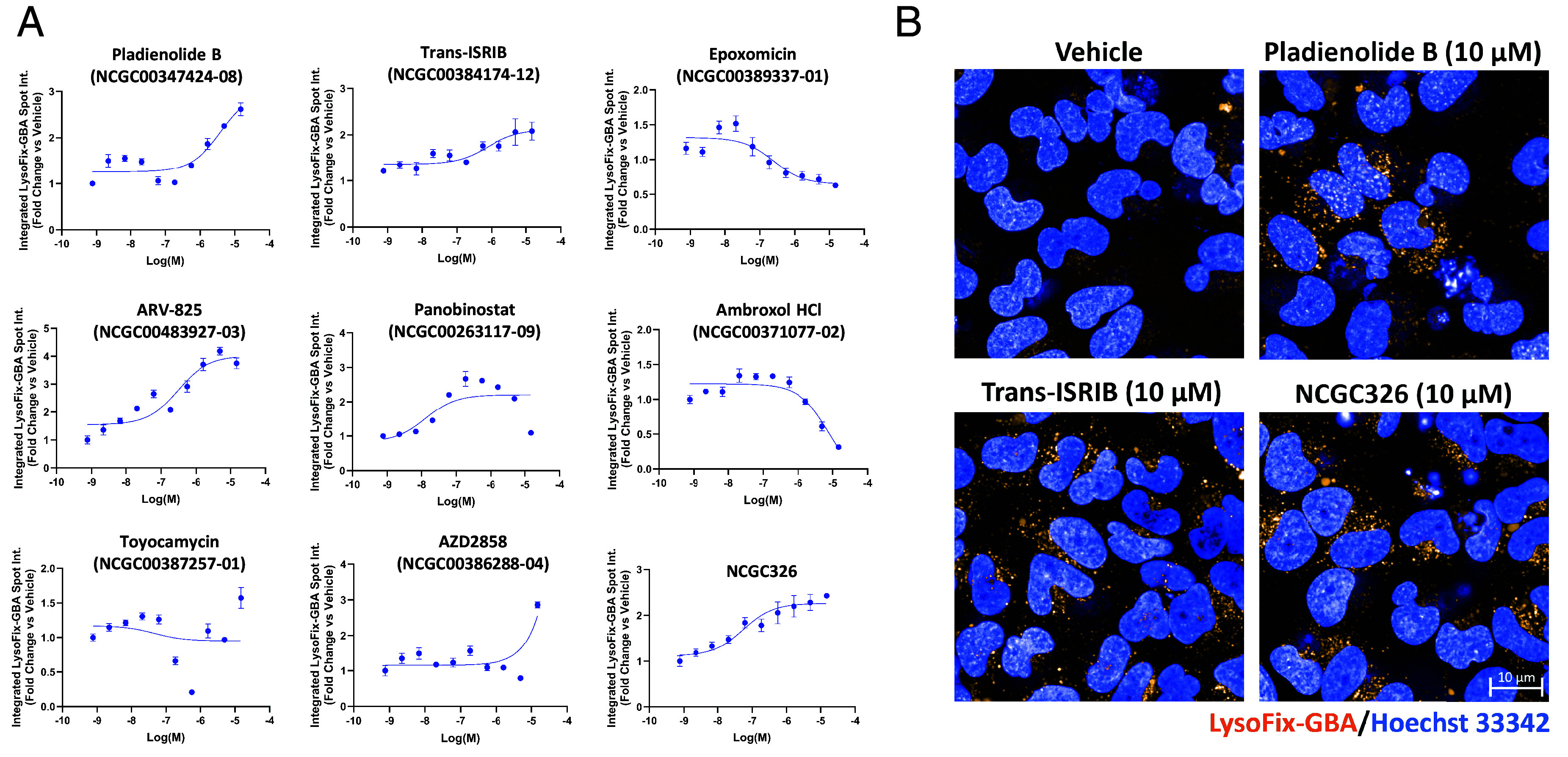
A high-content imaging-based secondary assay using the fluorescence-quenched substrate LysoFix-GBA quantifies GCase activity in the lysosome. Lysosomal activity of GCase was directly visualized using the optimized LysoFix-GBA secondary assay in live HiBiT-GCase-L444P H4 cells treated with vehicle (DMSO) or hit compounds. H4 cells were seeded into 384-well PerkinElmer PhenoPlates (25,000 cells in 40 μL media) and incubated for 24 h. Thereafter, the cells were treated with a titration of compounds for 24 h and then incubated with LysoFix-GBA (5 μM) for 2 h at 37 °C and 5% CO_2_. High-content imaging was performed after 15 min of nuclear staining with Hoechst-33342 (1 μg/mL) in Fluorobrite media. (*A*) Select hits from the primary screen were tested in a dose–response titration series. Data are represented as the fold change (compound-treated vs. DMSO-treated) in integrated LysoFix-GBA spot intensity per cell. Dose–response curves were fit using log(agonist) vs. response (three parameters). (Error bars: SEM [*n* = 3 to 6]). (*B*) Representative images are shown for pladienolide B, trans-ISRIB, and NCGC326 at their most effective concentrations in the LysoFix-GBA assay. (Scale bar, 10 μm.)

Following optimization of the LysoFix-GBA assay in H4 cells, it was deployed on hits arising from the HiBiT-GCase-L444P primary screen, including representatives from each mechanistic cluster (Fig. 2*C*). NCGC326, pladienolide B, panobinostat, and ARV-825 showed the strongest dose-dependent enhancement of GCase-L444P lysosomal activity (two- to fourfold) after 24 h of compound incubation (Fig. 3*A*). Trans-ISRIB showed moderate efficacy in the LysoFix-GBA assay after 24 h (Fig. 3 *A* and *B*), and its effect size was further increased after 72 h of compound incubation (*SI Appendix*, Fig. S10). Interestingly, response to ambroxol peaked at 80 to 160 nM concentration after both 24 h and 72 h of compound incubation; high concentrations (>5 μM), which stabilized HiBiT-GCase-L444P protein levels, were found to have an inhibitory effect on enzymatic activity (Fig. 3*A* and *SI Appendix*, Fig. S10), as expected for an active-site-directed PC (46). In contrast, micromolar concentrations of noninhibitory, allosteric-site-directed PC NCGC326 increased GCase-L444P lysosomal activity up to twofold after 24 h (Fig. 3 *A* and *B*) or 72 h (*SI Appendix*, Fig. S10) of compound incubation. For certain PRs (e.g., panobinostat and toyocamycin), the observed hook effects are due to cytotoxicity (*SI Appendix*, Fig. S5), not enzymatic inhibition. As an orthogonal dose–response assay, LysoFix-GBA thus validated the pharmacology of several primary screen hits, including PC NCGC326 and some PRs, such as pladienolide B, trans-ISRIB, and ARV-825, providing strong support for these compounds increasing levels of functional GCase within lysosomes.

### An Immunofluorescence-Based High-Content Screening Assay Allows Direct Visualization of GCase Translocation to the Lysosome.

To directly visualize productive lysosomal trafficking of GCase, we utilized a new monoclonal GCase antibody, hGCase-1/23, which was raised against recombinant GCase (imiglucerase). Based on its glycosylation pattern, HiBiT-GCase-L444P is entirely retained in the ER (Fig. 1*F*), yet the hGCase-1/23 antibody fails to detect ER localization of HiBiT-GCase-L444P in H4 cells (Fig. 1*C*). The same observation was also noted in fibroblasts from patients homozygous for L444P in our previous work, where hGCase-1/23 was able to stain a small fraction of GCase-L444P in lysosomes but did not detect the ER-retained portion of the protein (57). These results indicate that hGCase-1/23 recognizes a tertiary structure that is disrupted in misfolded, ER-retained GCase-L444P. We hypothesized that small molecules which promote proper folding, maturation, and trafficking of GCase-L444P should restore its staining in lysosomes.

**Fig. 4.**
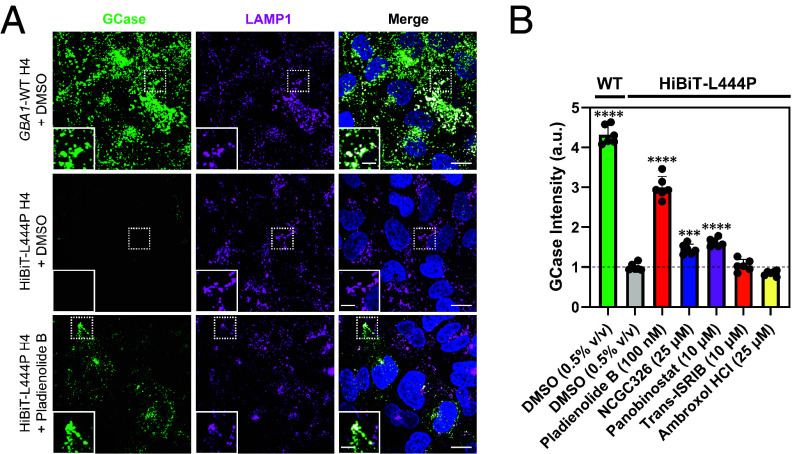
Immunofluorescence secondary assay measures GCase translocation to the lysosome via high-content imaging. HiBiT-GCase-L444P and unedited *GBA1*-WT H4 cells were seeded into 96-well PerkinElmer PhenoPlates and stained for GCase using monoclonal antibody hGCase-1/23, as well as for lysosomal marker LAMP1, following treatment with vehicle (DMSO, 0.5% v/v), pladienolide B (100 nM), NCGC326 (25 μM), panobinostat (10 μM), trans-ISRIB (10 μM), or ambroxol (25 μM) for ~34 h. Representative images are shown in (*A*), and data are quantified as mean GCase intensity in spots of LAMP1, summed per well, and normalized to cell count, in (*B*). (Error bars: SD [*n* = 6]). (Scale bar, 15 μm; *Inset* scale bar, 5 μm.) ***P-value ≤ 0.001 vs. HiBiT-L444P+DMSO; ****P-value ≤ 0.0001 vs. HiBiT-L444P+DMSO.

Costaining for GCase and lysosomal marker LAMP1 in 96-well plate format enabled HCS; data were quantified as GCase intensity in spots of LAMP1 and normalized to the number of nuclei. The assay demonstrated clear lysosomal localization of endogenous GCase in the *GBA1*-WT H4 line and a 77% reduction in signal in the HiBiT-GCase-L444P H4 line (Fig. 4 *A* and *B*). Treatment of HiBiT-L444P H4 cells with hit compound pladienolide B (100 nM) for ~34 h caused a threefold increase in lysosomal GCase staining (Fig. 4 *A* and *B*). In comparison, treatment with NCGC326 (25 μM) or panobinostat (10 μM) increased signal by 1.45- or 1.6-fold, respectively, relative to DMSO control (Fig. 4*B*). Collectively, these results demonstrate the utility of hGCase-1/23 for HCS, enabling visual representation of GCase translocation by PRs and PCs.

### NCGC326 Is a PC and a Weak Allosteric Activator of GCase-L444P.

We have thus far presented evidence that NCGC326 is a direct binder of GCase (Fig. 2*E*), a stabilizer of GCase-L444P protein levels (Fig. 2*C*), and an enhancer of GCase-L444P lysosomal translocation (Fig. 4*B*) and activity (Fig. 3). These results are highly suggestive of its role as a PC, whereby it offers protection against ERAD and enables productive trafficking of the enzyme to the lysosome. However, we have previously reported that noninhibitory PCs of GCase, including the NCGC607 series, can fulfill a dual role by behaving as enzyme activators that accelerate the cleavage of specific substrates (79). Enzyme activators reduce the activation energy of the reaction by increasing the affinity of the active site for the transition state, hence their activity is substrate dependent (49, 80). These two classes of small-molecule modulators of GCase—noninhibitory PCs and allosteric activators—likely induce distinct conformations in the enzyme, so the two effects do not necessarily correlate. To assist further deconvolution of the mechanism of action of NCGC326, we performed pure activator assays using the synthetic fluorescent substrate 4-methylumbelliferyl-β-D-glucopyranoside (4-MUG) and GCase that was sourced from clinical infusions (recombinant GCase-WT) or lysates of H4 cells and human fibroblasts (*SI Appendix*, Fig. S11). To best reproduce physiological conditions, including the presence of natural activators of GCase and the absence of artificial activators (sodium taurocholate), we also performed the activation assay using homogenate of spleen tissue from a patient with GD (N370S/N370S) (*SI Appendix*, Fig. S12) (48). Evaluating NCGC326 in these assays confirmed that, as an analog of NCGC607, it modestly increases GCase enzymatic activity toward cleavage of 4-MUG (*SI Appendix*, Figs. S11 and S12). Compared to known allosteric activator LTI-291/BIA 28-6156, NCGC326 is more potent and efficacious in the HiBiT assay (Fig. 2*C* and *SI Appendix*, Fig. S7*A*), but it performed significantly worse in the GCase activation assays (*SI Appendix*, Figs. S11 and S12), suggesting chaperoning of GCase as its major effect. PRs like pladienolide B and trans-ISRIB, which are not expected to directly bind GCase, did not alter its enzymatic activity in the activation assays (*SI Appendix*, Figs. S11*B* and S12*A*).

### Matrix Combination Screening Approach Identifies Coformulations of PCs and PRs which Synergistically Increase HiBiT-GCase-L444P Levels.

**Fig. 5.**
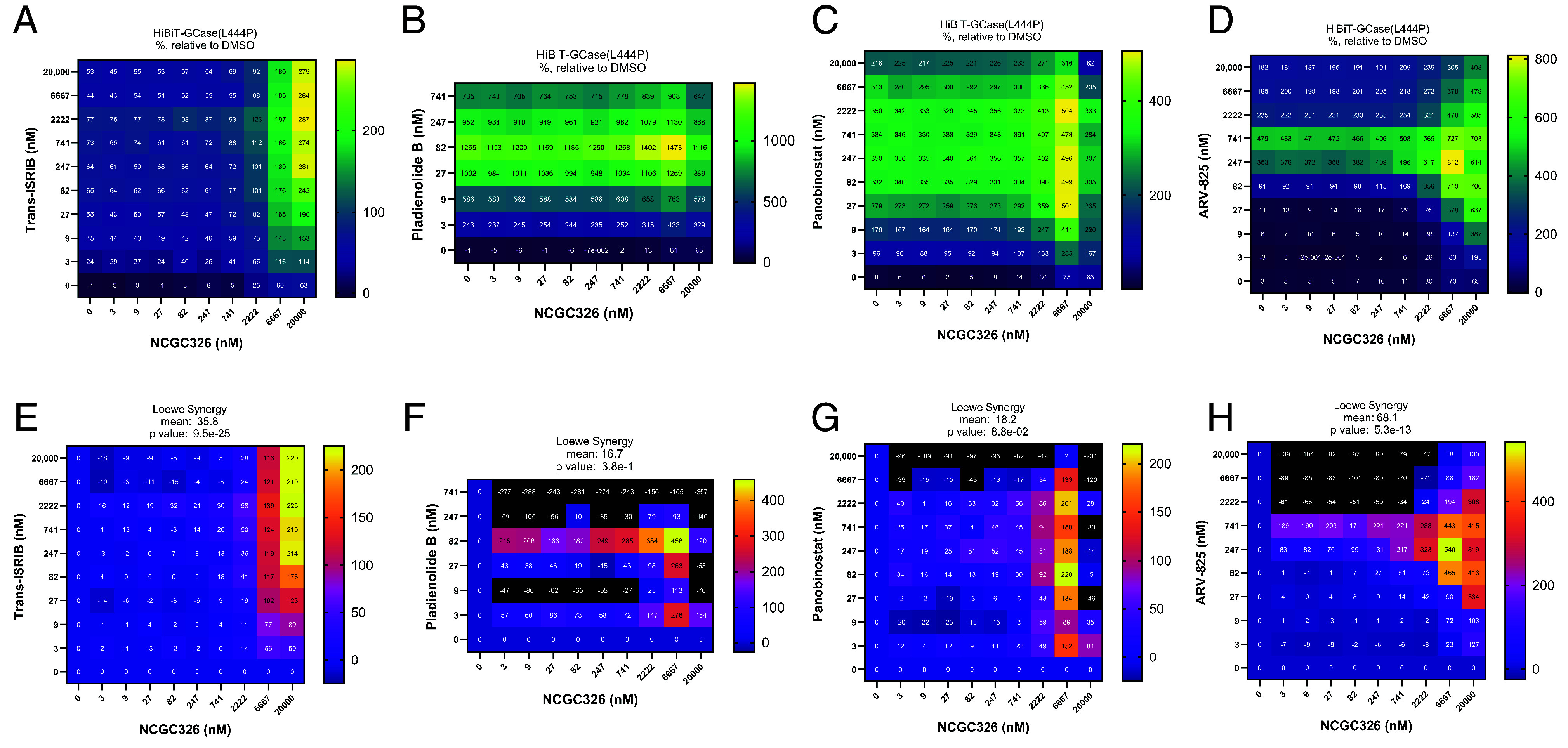
Matrix combination screening approach identifies synergistic coformulations of a PC with a proteostasis regulator. HiBiT-GCase-L444P H4 cells were tested in 10 × 10 pairwise dose–response combinatorial matrix format. Cells were treated for 24 h with chaperone NCGC326 in a 9-point titration (3 nM to 20 μM, 3× dilution) against the same 9-point titration of PRs trans-ISRIB (*A*), pladienolide B (*B*), panobinostat (*C*), or ARV-825 (*D*); the HiBiT-GCase lytic assay was then performed. Luminescence response values were normalized to intraplate DMSO-treated controls, such that 100% efficacy reflects a doubling of HiBiT-GCase levels (*A*–*D*). Synergy was evaluated based on the Loewe synergy score (*E*–*H*). In general, negative, zero, and positive synergy scores indicate antagonistic, additive, and synergistic interactions, respectively, between drugs. *n* = 3.

**Fig. 6.**
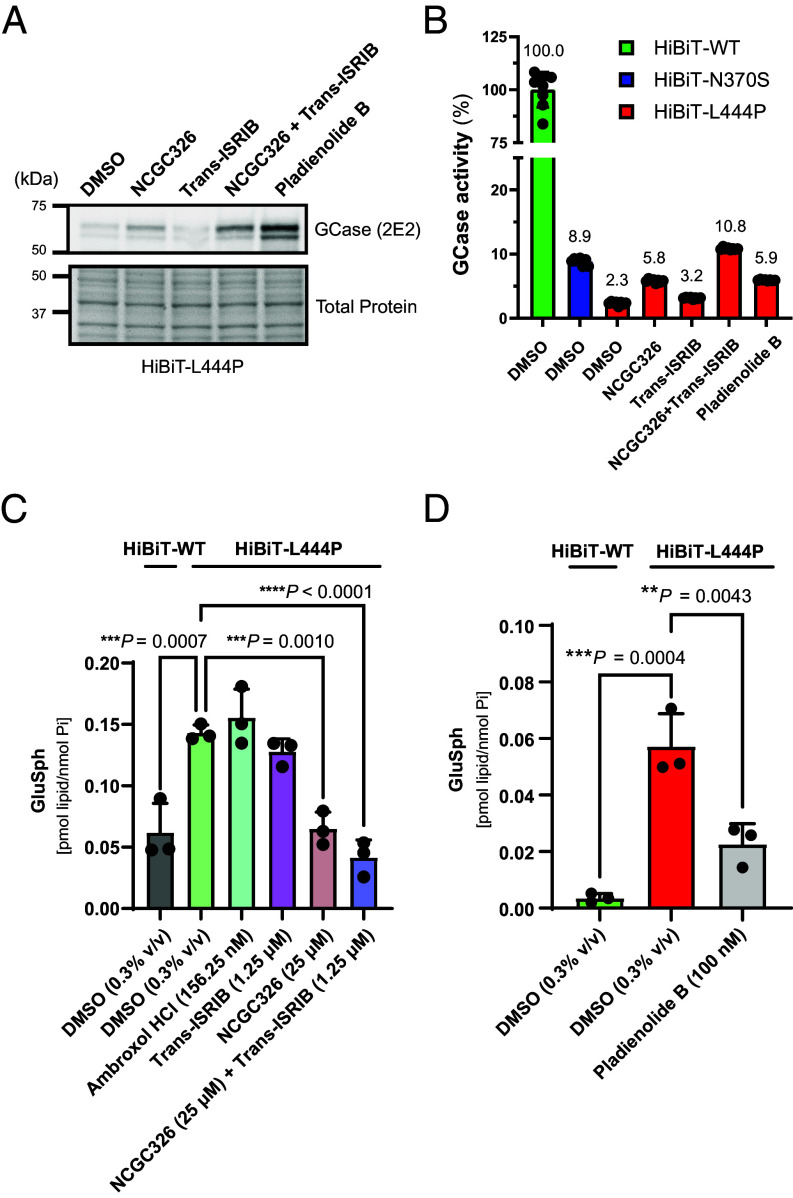
Screening hits reverse glycosphingolipid substrate accumulation in H4 cells. (*A* and *B*) HiBiT-GCase-L444P H4 cells were treated (starting at 50 to 60% confluence) with vehicle (DMSO, 0.3% v/v), NCGC326 (25 μM), trans-ISRIB (1.25 μM), or the combination of NCGC326 (25 μM) and trans-ISRIB (1.25 μM) for 3 d, and (starting at 90 to 100% confluence) with pladienolide B (100 nM) for 24 h. (*A*) GCase protein levels in cell lysates were visualized on western blot using the 2E2 antibody, with total protein as the loading control. (*B*) GCase activity in cell lysates of HiBiT-GCase H4 cells (WT, N370S, or L444P) treated with DMSO or compounds. Relative GCase activity was calculated by adjusting for protein concentration, correcting for *GBA1*-KO H4 cell background, and normalizing to HiBiT-WT+DMSO signal. (Error bars: SD [*n* = 8 technical replicates]). (*C* and *D*) Levels of glucosylsphingosine (GluSph) in HiBiT-GCase-WT and HiBiT-GCase-L444P H4 reporter lines were evaluated by supercritical fluid chromatography (SFC) separation coupled with tandem mass spectrometry (MS/MS) detection and normalized to total cellular inorganic phosphate (P_i_) levels. Cells were treated with vehicle (0.3% v/v DMSO) or hit compounds for 3 d (*C*), except for the cytotoxic hit compound pladienolide B, for which treatment lasted 48 h (*D*). (Error bars: SD [*n* = 3 biological replicates]).

Following the discovery of several PRs that increase GCase-L444P levels through distinct mechanisms of action (Fig. 2*C*), we sought to determine whether these PRs would synergize with a PC of GCase (27). We performed a matrix combinatorial screening assay (Fig. 5), in which a titration series of PC NCGC326 was screened in pairwise combination against a titration series of PRs representing different mechanistic classes. Potential synergy was evaluated based on the HiBiT-GCase-L444P response matrix (Fig. 5 *A*–*D* and *SI Appendix*, Fig. S13*A*), which was used to calculate the Loewe synergy score (Fig. 5 *E*–*H* and *SI Appendix*, Fig. S13*B*). The combinations of NCGC326 with PRs ISRIB (Fig. 5 *A* and *E*) or ARV-825 (Fig. 5 *D* and *H*) displayed the greatest synergy across the entire matrix. Pladienolide B and panobinostat also synergized with NCGC326 while inducing great overall fold changes in HiBiT-GCase-L444P levels (Fig. 5 *B* and *C*); however, at high concentrations, their synergy (Fig. 5 *F* and *G*) was constrained by strong cytotoxicity (*SI Appendix*, Fig. S5). Synergistic effects between NCGC326 and trans-ISRIB were further observed by western blot (Fig. 6*A*) and GCase activity assay (Fig. 6*B*).

### Selected Small Molecules Successfully Rescue the Metabolic Defect of GD in HiBiT-GCase-L444P H4 Cells.

GD is a metabolic disorder that leads to cellular accumulation of pathological glycosphingolipid species, GluCer and GluSph, which are substrates of GCase. We hypothesized that small molecules which promote GCase-L444P folding, trafficking, and lysosomal activity would correct the biochemical defect contributing to substrate accumulation. HiBiT-GCase-L444P H4 cells were treated for 3 d (starting at 60 to 70% confluence) with either DMSO (0.3% v/v) or hit compounds ambroxol (156.25 nM), trans-ISRIB (1.25 μM), and NCGC326 (25 μM), as well as the combination of NCGC326 with trans-ISRIB; dose selection was guided by 72 h testing with LysoFix-GBA (*SI Appendix*, Fig. S10). Levels of GluSph (Fig. 6*C*) were then quantitated by supercritical fluid chromatography (SFC) separation coupled with tandem mass spectrometry (MS/MS) detection (SFC-MS/MS) (15, 58). Impaired clearance of GluSph resulted in threefold or higher accumulation of the substrate in HiBiT-GCase-L444P H4 cells, relative to the HiBiT-GCase-WT line, providing a moderate window to interrogate the effect of hit compounds (Fig. 6 *C* and *D* and *SI Appendix*, Fig. S14). NCGC326, as a single agent or in combination with trans-ISRIB, completely reversed lipid accumulation in HiBiT-GCase-L444P H4 cells, to HiBiT-GCase-WT levels (Fig. 6*C*). A lower concentration of NCGC326 (10 μM) was also effective in reversing GluSph accumulation in the HiBiT-GCase-L444P line (*SI Appendix*, Fig. S14). Ambroxol and trans-ISRIB did not show an effect on lipid accumulation at the concentrations tested (Fig. 6*C*). H4 cells were treated with pladienolide B (100 nM) for only 48 h (starting at 80 to 90% confluence), due to the severe cytotoxic effect of the compound (*SI Appendix*, Fig. S5); a reduction in GluSph levels was observed (Fig. 6*D*). Collectively, results from the primary screen, both secondary assays, and the lipid functional endpoint support the designation of pladienolide B and NCGC326 as true GCase-L444P enhancers in H4 cells.

### Small-Molecule Stabilizers of GCase-L444P Also Modulate GCase-WT and GCase-N370S in H4 Cells and Show Efficacy in Patient-Derived Fibroblasts.

To examine the relevance of our assay pipeline for other *GBA1* genotypes, we tested select compounds (NCGC326, pladienolide B, and trans-ISRIB) in the HiBiT (*SI Appendix*, Fig. S15 *A*–*C*) and LysoFix-GBA (*SI Appendix*, Fig. S15 *D* and *E*) assays using the HiBiT-GCase-WT and HiBiT-GCase-N370S H4 reporter cell lines. After 24 h of treatment, NCGC326 stabilized HiBiT-GCase protein levels more effectively in the HiBiT-GCase-WT line than in the HiBiT-GCase-N370S line (*SI Appendix*, Fig. S15 *A*–*C*), although the LysoFix-GBA responses were similar (*SI Appendix*, Fig. S15*E*). GCase-WT and GCase-N370S responded to pladienolide B with similar potency but lower efficacy than GCase-L444P in the HiBiT assay (*SI Appendix*, Fig. S15 *B* and *C*). HiBiT-WT and HiBiT-N370S H4 cells were then treated for 3 d with the three single agents or the combination of NCGC326 and trans-ISRIB, and GCase protein levels were evaluated by western blot (*SI Appendix*, Fig. S16). Pladienolide B showed a pronounced effect, while NCGC326 and trans-ISRIB continued to demonstrate an apparent synergy. Finally, the compounds were validated in another cell model, fibroblasts from a healthy control (WT/WT) or patients with GD and *GBA1* genotype N370S/N370S or L444P/L444P (*SI Appendix*, Fig. S17). After 6 d of treatment, pladienolide B showed a highly potent pharmacological profile in the LysoFix-GBA assay (*SI Appendix*, Fig. S17*B*), and stabilization of GCase protein levels in L444P/L444P fibroblasts was confirmed by western blot (*SI Appendix*, Fig. S17*C*). Taken together, these results suggest that our preclinical pipeline can discover small-molecule modulators of WT-, N370S-, and L444P-GCase. N370S and L444P are the most common pathogenic *GBA1* variants and are the most relevant to *GBA1*-PD (81). Yet, interventions targeting GCase-WT could have considerable impact for the broader PD population, as it has been reported that, even in idiopathic PD, GCase levels tend to be low, reflecting an inverse relationship between GCase and α-synuclein (82).

## Discussion

A major challenge in the field of small-molecule GCase enhancers is the lack of adequate cell-based assays amenable to HTS format which can drive the identification, pharmacological validation, and stepwise medicinal chemistry optimization of new chemical matter. To enable rational development of GCase enhancers as a therapeutic strategy, we leveraged an integrated approach, using basic science knowledge surrounding the *GBA1* molecular target to inform the design of physiologically relevant, translational assays that can accelerate the drug discovery process.

Based on the current paradigm, the pathophysiology of GCase deficiency extends largely from missense mutations in *GBA1* leading to reduced folding efficiency and/or structural instability of the protein and, consequently, its increased targeting for ERAD. Importantly, these missense-mutant proteins could be catalytically competent in the lysosomal environment, where low pH and high substrate concentration provide a stabilizing milieu (21, 22), but their inability to bypass the ER quality control system obstructs trafficking. In particular, the L444P variant of GCase suffers from a perturbed hydrophobic core on a noncatalytic domain (26), which causes overzealous ERAD (20) and correlates with a clinically severe phenotype. ERAD is the rate-limiting step in the folding of mutant GCase (69), as it controls the reservoir of folding intermediates retained in the ER. The ERAD machinery is part of the larger proteostasis network, which also consists of proteinaceous molecular chaperones that promote protein folding, as well as stress-responsive signaling pathways, such as the UPR, heat shock response, and integrated stress response (ISR), that provide dynamic regulation. Aging-related decline in the robustness and capacity of the proteostasis network escalates protein misfolding and partially underlies the development of neurodegenerative diseases, such as PD (31).

The proteostasis network represents a rational entry point for stabilization of mutant GCase levels, either through enhanced folding mediated by molecular chaperones or through escape from ERAD (22, 69). Its modification can be achieved with small-molecule PRs, which are a generic biological approach to enzyme enhancement with wide applicability to LSDs and other protein-misfolding diseases (22, 27, 30). PCs are a separate class of small molecules that are tailored for GCase stabilization through direct binding (42); their mechanisms of action can include increasing the thermodynamic stability or kinetic accessibility of the native state, or otherwise inducing conformations that facilitate enzyme trafficking (33–36, 63). After PCs and PRs act on mutant GCase to increase its ER export, it passes to the Golgi network, where it undergoes maturation of its glycosylation status before final trafficking to the lysosome. Translocation of GCase from the ER to the lysosome depends upon sorting receptor LIMP-2 (63) and cochaperone progranulin (83), while its optimal lysosomal activity requires cofactor saposin C (17, 36). Numerous other genetic modifiers of GCase trafficking and activity likely exist, potentially contributing to the phenotypic heterogeneity observed in GD and the variable penetrance of *GBA1*-PD (84, 85).

Most prior efforts to identify small-molecule modulators of GCase have failed to incorporate these cellular complexities in the discovery phase, relying instead on biochemical assays with purified protein or homogenates (39, 40, 48). Such assays do not align with the true function of PCs, which is salvage of misfolded protein from ERAD, nor do they allow for the identification of PRs, which indirectly regulate GCase through their effects on distinct molecular pathways. Furthermore, cell-free biochemical assays neglect factors such as membrane permeability, intracellular bioavailability, or cytotoxicity. Targeting GCase enhancement in live cells is thus better suited to identify physiologically relevant small-molecule therapeutics. To this end, we assembled a toolkit of complementary, high-throughput cellular assay modalities.

The primary assay in this tiered pipeline focuses on stabilization of the severely misfolded, ERAD-prone L444P variant of GCase in whole cells. To measure GCase protein abundance, we appended a small HiBiT peptide reporter tag to GCase. HiBiT (1.3 kDa) undergoes high-affinity structural complementation with exogenous LgBiT (18 kDa) in a lytic assay, reconstituting an active luciferase enzyme that affords a sensitive and quantitative readout. This method of tagging was selected over alternative, larger-size reporters, such as NanoLuc luciferase (19 kDa), HaloTag (33 kDa), and GFP (27 kDa), as we hypothesized it would be less likely to perturb protein–protein interactions involved in the degradation or trafficking of GCase (86). Our results indicate that an N-terminal HiBiT tag has negligible influence on GCase maturation, trafficking, and function. To be considered reliable, a quantitative reporter must represent the target biology with high fidelity. Previous studies indicate that stable integration of a HiBiT reporter, via gene editing, more faithfully maintains biological function compared to plasmid-based overexpression systems (87). Moreover, overexpression of proteins can have mechanistic consequences, including overload of protein quality-control machinery, promiscuous interactions, and stochiometric imbalance (88), which could generate artifacts. Given these considerations, we utilized TALEN-mediated gene editing (61, 62) to stably incorporate a single copy of the HiBiT-tagged *GBA1* variant into an intragenic safe-harbor locus within a *GBA1*-KO background, as affirmed by ddPCR of the selected clones; this workflow constrained *GBA1* expression to a level comparable with the endogenous alleles in the hypertriploid *GBA1*-WT H4 cell line.

When coupled with titration-based qHTS on a fully integrated robotic screening platform, the HiBiT-GCase-L444P assay generates high-quality pharmacological data, in the form of efficacy and potency values extracted from concentration–response curves (47). We deployed the assay in a screen of 10,779 small molecules within the NCATS bioactive collections, which include approved drugs, investigational agents, and annotated tool compounds (59). The detection of chemical matter with established relevance to GCase biology provided confidence in the HiBiT-GCase-L444P screening approach. Ambroxol, a known active-site-directed PC (20, 43, 66) currently being evaluated in a multicenter Phase 3A clinical trial for patients with PD and known *GBA1* status (NCT05778617), was identified as a highly active compound in the unbiased primary screen. Furthermore, the screen identified 32 compounds with efficacy > 300% (fourfold higher than DMSO), of which 27 (84%) were annotated as HDAC inhibitors. As PRs, HDAC inhibitors can reprogram the proteostasis network by causing posttranslational hyperacetylation of histones, transcription factors, and molecular chaperones (71, 89). This class of small molecules has been investigated in the context of numerous protein-misfolding diseases, including cystic fibrosis (89) and Niemann-Pick disease (90). The compounds have also been previously reported as enhancers of mutant GCase that might be developed as GD therapeutics (71, 72). For example, the HDAC inhibitor vorinostat increased the half-life of L444P-mutant GCase by reducing its targeting for ERAD through the molecular chaperone HSP90β (71, 72). Notably, we have not defined the precise contributions of transcriptional versus posttranslational effects of the HDAC inhibitors on GCase in the H4 model system.

The HiBiT primary screen was also able to rank order analogs of in-house lead PCs NCGC607 and NCGC758, indicating its value as a phenotypically relevant, cell-based tool for lead optimization via medicinal chemistry efforts. Importantly, NCGC326 was identified as a novel analog of NCGC607 with a nearly threefold improvement in efficacy and similar potency. While NCGC758 was inactive as a stabilizer of L444P-mutant GCase, its analog, LTI-291/BIA 28-6156 (75, 76), a compound currently being evaluated in a Phase 2 clinical trial for patients with *GBA1*-PD (NCT05819359), was detected as a hit with similar efficacy to NCGC607. Collectively, these results underscore the reliability of the HiBiT-GCase-L444P assay, its ability to detect both PCs and PRs, and its utility as a workhorse for lead discovery and development.

In addition to validating the high-throughput assay, our primary screen of annotated, chemogenomic libraries enables both drug repositioning and hypothesis generation, which can guide the identification of novel molecular targets regulating the *GBA1* pathway. Analysis of the target profiles of 140 confirmed hit compounds revealed 30 enriched molecular targets, which mainly represented epigenetic regulators, such as HDACs 1-11, NCOR1/2, bromodomain-containing proteins (BRD2, BRD4, and BRDT), CHD4, and SIRT6 (*SI Appendix*, Fig. S6*B*). The analysis also implicated the Wnt/β-catenin signaling pathway, which is known to be impaired under GCase deficiency (91). Five confirmed hits inhibited GSK3β, a negative Wnt regulator, while another hit (SKL2001) directly activated Wnt signaling. In agreement, pharmacological Wnt activation was previously shown to rescue defects in dopaminergic neurogenesis (91) and bone matrix deposition (92) in iPSCs derived from patients with GD. Across the identified molecular targets, the top enriched pathway was NOTCH signaling (*SI Appendix*, Table S2), which has also been previously linked to *GBA1*-PD (93).

Two of the most potent hits identified in our screen were pladienolide B (EC_50_ = 9.4 nM) and trans-ISRIB (EC_50_ = 11.9 nM). Pladienolide B is an inhibitor of the splicing factor SF3B1 (94) that causes production of alternatively spliced transcripts (95). While this molecular target has translational potential (95), its mechanistic linkage to GCase stabilization is unclear. Prior studies indicate that low levels of pladienolide B enhance protein-folding capacity by modulating the levels of molecular chaperones (96). As a PR, ISRIB inhibits the ISR, which responds to the presence of misfolded proteins by reducing global protein synthesis rates (97). Interestingly, ISRIB inhibits chronic, low-grade ISR activation but spares acute, cytoprotective ISR activation, explaining its lack of overt toxicity in vivo (98). The potency with which ISRIB modifies its target [IC_50_ = 5 nM (73)] aligns with the value determined in our primary screen. Furthermore, its favorable safety profile combined with its cognitive memory-enhancing effects (73) and its demonstrated efficacy in animal models of neurodegenerative disease, such as prion disease (99) and Alzheimer’s disease (100), make it an attractive therapeutic candidate for *GBA1*-PD. Recently, ISRIB was derivatized into the investigational drug ABBV-CLS-7262, which is under clinical trials for Amyotrophic Lateral Sclerosis (Phase 2/3; NCT05740813) and Vanishing White Matter disease (Phase 1B/2; NCT05757141). Based on their intriguing mechanisms of action, pladienolide B and ISRIB represent high-priority hits for subsequent validation.

Owing to their distinct mechanisms of action, PCs and PRs are candidates for coformulation into a synergistic combination therapy (27). This approach is reminiscent of the fixed-dose triple combination therapy used to treat protein misfolding in cystic fibrosis, which involves coadministration of two orthogonal PCs with a CFTR channel gating potentiator (101). We hypothesized that PRs would increase the population of folded GCase-L444P in the ER, upon which PCs could then act, thus synergistically installing an export-permissive, corrective environment in the ER (27). To facilitate design of a highly potent and efficacious combination therapy, we deployed a matrix combination screening approach (102, 103) using our HiBiT-GCase-L444P qHTS platform. The combinations of PC NCGC326 with PRs trans-ISRIB or ARV-825 displayed the greatest synergy. Given these results, the widespread deployment of HiBiT-driven matrix combination screening may provide a general strategy to identify combination therapies for other LSDs and LOF conditions.

Since our drug discovery pipeline addresses GD as a protein-misfolding disease, focusing on defects in GCase folding, trafficking, and lysosomal activity, complementary evidence must be provided at the level of the biochemical phenotype, which is most relevant to the disease pathology. Increments in GCase activity as measured by hydrolysis of a synthetic fluorescent substrate, like LysoFix-GBA, might not correlate with improvement in hydrolysis of the natural substrates, GluCer and GluSph (49, 80). GluCer is the primary storage product in GD and the major glycolipid substrate of GCase; GluSph is the minor glycolipid substrate, being degraded with a ~100-fold lower turnover number, and its accumulation is thought to be largely driven by deacylation of GluCer by acid ceramidase (104, 105). Since GluSph is almost undetectable in normal tissues, it represents a sensitive and specific biomarker for GD and its response to therapy (106). Pathologically, GluSph is neurotoxic (107, 108), possibly as a result of its amphipathic nature and effects on lysosomal membrane permeabilization (109), and it accumulates up to 1,000-fold in the brains of patients with neuronopathic GD (110). Collectively, these features render GluSph a suitable functional endpoint for evaluation of candidate lead compounds. Given that our HiBiT-GCase-L444P H4 cell model exhibited threefold or higher accumulation of GluSph relative to the HiBiT-GCase-WT line, we tested whether the GCase stabilizers discovered from our pipeline could rescue the lipid phenotype by enhancing intralysosomal active GCase. NCGC326 completely reversed the biochemical defect, underscoring the capacity of our pipeline to identify physiologically relevant chemical matter. Interestingly, ambroxol and trans-ISRIB did not lower GluSph levels, despite enhancing GCase-L444P lysosomal activity at the concentrations tested, demanding further investigation.

In this work, we develop and deploy a suite of novel, high-throughput-amenable, quantitative assay technologies that can further the development of small-molecule therapeutic strategies to enhance GCase. Despite the strengths of our assays, the study has some limitations. The chief liability of our HiBiT-based primary assay is that the reporter tag was not appended to the endogenous *GBA1* allele. Rather, exogeneous HiBiT-tagged *GBA1* is constitutively expressed from cDNA integrated into a safe-harbor site within a *GBA1*-KO background; therefore, the model is geared to capture posttranslational dynamics and is inadequate to identify GCase stabilizers acting through direct epigenetic or transcriptional regulation from the native locus (111, 112). Moreover, the HiBiT assay was executed in an immortalized human cell line (H4 neuroglioma), which may feature differential wiring or capacity of the proteostasis network relative to patient cells. To begin to address this limitation, we performed hit validation in patient-derived fibroblasts using the LysoFix-GBA assay; however, our secondary assays should be further adapted to patient-derived macrophages or neurons for optimal physiological relevance to GD and PD (77). Future experiments would benefit from assay technologies enabling measurement of glycosphingolipid substrate levels in high-throughput fashion, including in cell models with greater fold lipid accumulation. Our matrix combination screening approach focused on synergistic combinations of a single PC with select PRs; however, it is possible that dissimilar PRs acting on different modules in the proteostasis network could also exhibit synergy (69). This would be better explored with an all-versus-all matrix design (103). Finally, while the small molecules identified in this work may hold therapeutic potential for *GBA1*-PD, or even sporadic PD (3, 14, 15), our pipeline does not feature assays for quantitation of a PD phenotype, such as α-synuclein aggregation (50).

In conclusion, we integrated basic science knowledge surrounding the *GBA1* target with translational expertise to construct a multilevel drug discovery pipeline for GD and *GBA1*-PD. Our approach identified small molecules that increase GCase-L444P protein levels (HiBiT-GCase assay), lysosomal activity (LysoFix-GBA assay), and lysosomal translocation (hGCase-1/23 immunofluorescence assay). These include a PR with a novel mechanism of action, pladienolide B, and an improved derivative of an existing PC, NCGC326. Both reversed GluSph accumulation in the H4 model. Finally, we used a combinatorial matrix screening approach to identify synergistic actions of PRs with a PC in enhancing GCase-L444P levels. The hits identified in this screen and validated in orthogonal assays should be prioritized for further investigation, with the goal of providing a potent and efficacious therapeutic for patients with neuronopathic GD, *GBA1*-PD, or other diseases associated with GCase activity defects (113, 114).

## Materials and Methods

A detailed *Materials and Methods* section is provided in *SI Appendix*. This section includes a description of 1) plasmid construction; 2) H4 cell culture and transfection; 3) stable integration of HiBiT-*GBA1* transgene into H4 *GBA1*-knockout cell line; 4) copy number determination assay; 5) LIMP-2 knockdown in H4 cells; 6) western blotting; 7) glycosylation analysis (Endo H and PNGase F assay); 8) AlphaLISA; 9) GCase activity assay (4-MUG); 10) immunocytochemistry; 11) lipidomic analysis; 12) microscale thermophoresis; 13) miniaturization and high-throughput screening of HiBiT-GCase and CellTiter-Glo assays; 14) primary screen hit selection; 15) target profile and pathway analysis of hit compounds; 16) miniaturization and high-throughput screening of LysoFix-GBA secondary assay; 17) activator assays; 18) drug synergy evaluation; 19) fibroblast studies; 20) preparation of N-(4-iodophenyl)-2-(2-((4-iodophenyl)amino)-2-oxoethoxy)benzamide (NCGC00241326); and 21) statistical analysis.

## Supplementary Material

Appendix 01 (PDF)

Dataset S01 (XLSX)

Dataset S02 (XLSX)

## Data Availability

Final primary screen hits arising from this work are listed in *SI Appendix*, Table S1. All study data are included in the article and/or supporting information.

## References

[1] E. Sidransky , Multicenter analysis of glucocerebrosidase mutations in Parkinson’s disease. N. Engl. J. Med. **361**, 1651–1661 (2009). 19846850 10.1056/NEJMoa0901281 PMC2856322

[2] M. A. Nalls , A multicenter study of glucocerebrosidase mutations in dementia with Lewy bodies. JAMA Neurol. **70**, 727–735 (2013). 23588557 10.1001/jamaneurol.2013.1925 PMC3841974

[3] F. Richter , A GCase chaperone improves motor function in a mouse model of synucleinopathy. Neurotherapeutics **11**, 840–856 (2014). 25037721 10.1007/s13311-014-0294-x PMC4391384

[4] S. P. Sardi , Augmenting CNS glucocerebrosidase activity as a therapeutic strategy for Parkinsonism and other Gaucher-related synucleinopathies. Proc. Natl. Acad. Sci. U.S.A. **110**, 3537–3542 (2013). 23297226 10.1073/pnas.1220464110 PMC3587272

[5] J. Maple-Grødem , Association of GBA genotype with motor and functional decline in patients with newly diagnosed Parkinson disease. Neurology **96**, e1036–e1044 (2021). 33443131 10.1212/WNL.0000000000011411 PMC8055329

[6] A. A. Szwedo , GBA and APOE impact cognitive decline in Parkinson’s disease: A 10-year population-based study. Mov. Disord. **37**, 1016–1027 (2022). 35106798 10.1002/mds.28932 PMC9362732

[7] N. W. Barton , Replacement therapy for inherited enzyme deficiency–macrophage-targeted glucocerebrosidase for Gaucher’s disease. N. Engl. J. Med. **324**, 1464–1470 (1991). 2023606 10.1056/NEJM199105233242104

[8] N. J. Weinreb , Effectiveness of enzyme replacement therapy in 1028 patients with type 1 Gaucher disease after 2 to 5 years of treatment: A report from the Gaucher Registry. Am. J. Med. **113**, 112–119 (2002). 12133749 10.1016/s0002-9343(02)01150-6

[9] A. Gehrlein , Targeting neuronal lysosomal dysfunction caused by β-glucocerebrosidase deficiency with an enzyme-based brain shuttle construct. Nat. Commun. **14**, 2057 (2023). 37045813 10.1038/s41467-023-37632-4 PMC10097658

[10] G. Altarescu , The efficacy of enzyme replacement therapy in patients with chronic neuronopathic Gaucher’s disease. J. Pediatr. **138**, 539–547 (2001). 11295718 10.1067/mpd.2001.112171

[11] B. Rosenbloom , The incidence of Parkinsonism in patients with type 1 Gaucher disease: Data from the ICGG Gaucher Registry. Blood Cells Mol. Dis. **46**, 95–102 (2011). 21067946 10.1016/j.bcmd.2010.10.006 PMC4662380

[12] N. Giladi , Safety and efficacy of venglustat in GBA1-associated Parkinson’s disease: An international, multicentre, double-blind, randomised, placebo-controlled, phase 2 trial. Lancet Neurol. **22**, 661–671 (2023). 37479372 10.1016/S1474-4422(23)00205-3

[13] S. Mullin , Ambroxol for the treatment of patients with Parkinson disease with and without glucocerebrosidase gene mutations: A nonrandomized, noncontrolled trial. JAMA Neurol. **77**, 427–434 (2020). 31930374 10.1001/jamaneurol.2019.4611 PMC6990847

[14] L. F. Burbulla , A modulator of wild-type glucocerebrosidase improves pathogenic phenotypes in dopaminergic neuronal models of Parkinson’s disease. Sci. Transl. Med. **11**, eaau6870 (2019). 31619543 10.1126/scitranslmed.aau6870 PMC7359409

[15] J. R. Mazzulli , Gaucher disease glucocerebrosidase and α-synuclein form a bidirectional pathogenic loop in synucleinopathies. Cell **146**, 37–52 (2011). 21700325 10.1016/j.cell.2011.06.001 PMC3132082

[16] B. Liou , Analyses of variant acid beta-glucosidases: Effects of Gaucher disease mutations. J. Biol. Chem. **281**, 4242–4253 (2006). 16293621 10.1074/jbc.M511110200

[17] R. J. Tamargo, A. Velayati, E. Goldin, E. Sidransky, The role of saposin C in Gaucher disease. Mol. Genet. Metab. **106**, 257–263 (2012). 22652185 10.1016/j.ymgme.2012.04.024 PMC3534739

[18] M. Schmitz, M. Alfalah, J. M. Aerts, H. Y. Naim, K. P. Zimmer, Impaired trafficking of mutants of lysosomal glucocerebrosidase in Gaucher’s disease. Int. J. Biochem. Cell Biol. **37**, 2310–2320 (2005). 15982918 10.1016/j.biocel.2005.05.008

[19] I. Bendikov-Bar, M. Horowitz, Gaucher disease paradigm: From ERAD to comorbidity. Hum. Mutat. **33**, 1398–1407 (2012). 22623374 10.1002/humu.22124

[20] I. Bendikov-Bar, I. Ron, M. Filocamo, M. Horowitz, Characterization of the ERAD process of the L444P mutant glucocerebrosidase variant. Blood Cells Mol. Dis. **46**, 4–10 (2011). 21106416 10.1016/j.bcmd.2010.10.012

[21] A. R. Sawkar , Chemical chaperones and permissive temperatures alter localization of Gaucher disease associated glucocerebrosidase variants. ACS Chem. Biol. **1**, 235–251 (2006). 17163678 10.1021/cb600187q

[22] F. Wang, G. Agnello, N. Sotolongo, L. Segatori, Ca^2+^ homeostasis modulation enhances the amenability of L444P glucosylcerebrosidase to proteostasis regulation in patient-derived fibroblasts. ACS Chem. Biol. **6**, 158–168 (2011). 21043486 10.1021/cb100321m

[23] G. Babajani, M. B. Tropak, D. J. Mahuran, A. R. Kermode, Pharmacological chaperones facilitate the post-ER transport of recombinant N370S mutant β-glucocerebrosidase in plant cells: Evidence that N370S is a folding mutant. Mol. Genet. Metab. **106**, 323–329 (2012). 22592100 10.1016/j.ymgme.2012.04.018 PMC3425598

[24] S. S. Vembar, J. L. Brodsky, One step at a time: Endoplasmic reticulum-associated degradation. Nat. Rev. Mol. Cell Biol. **9**, 944–957 (2008). 19002207 10.1038/nrm2546 PMC2654601

[25] I. Ron, M. Horowitz, ER retention and degradation as the molecular basis underlying Gaucher disease heterogeneity. Hum. Mol. Genet. **14**, 2387–2398 (2005). 16000318 10.1093/hmg/ddi240

[26] H. Dvir , X-ray structure of human acid-beta-glucosidase, the defective enzyme in Gaucher disease. EMBO Rep. **4**, 704–709 (2003). 12792654 10.1038/sj.embor.embor873 PMC1326319

[27] T. W. Mu , Chemical and biological approaches synergize to ameliorate protein-folding diseases. Cell **134**, 769–781 (2008). 18775310 10.1016/j.cell.2008.06.037 PMC2650088

[28] D. S. Ong, T. W. Mu, A. E. Palmer, J. W. Kelly, Endoplasmic reticulum Ca^2+^ increases enhance mutant glucocerebrosidase proteostasis. Nat. Chem. Biol. **6**, 424–432 (2010). 20453863 10.1038/nchembio.368 PMC2873071

[29] D. S. Ong , FKBP10 depletion enhances glucocerebrosidase proteostasis in Gaucher disease fibroblasts. Chem. Biol. **20**, 403–415 (2013). 23434032 10.1016/j.chembiol.2012.11.014 PMC3624024

[30] B. Calamini , Small-molecule proteostasis regulators for protein conformational diseases. Nat. Chem. Biol. **8**, 185–196 (2011). 22198733 10.1038/nchembio.763 PMC3262058

[31] E. T. Powers, R. I. Morimoto, A. Dillin, J. W. Kelly, W. E. Balch, Biological and chemical approaches to diseases of proteostasis deficiency. Annu. Rev. Biochem. **78**, 959–991 (2009). 19298183 10.1146/annurev.biochem.052308.114844

[32] A. R. Sawkar , Chemical chaperones increase the cellular activity of N370S beta-glucosidase: A therapeutic strategy for Gaucher disease. Proc. Natl. Acad. Sci. U.S.A. **99**, 15428–15433 (2002). 12434014 10.1073/pnas.192582899 PMC137733

[33] J. Zheng , β-glucocerebrosidase modulators promote dimerization of β-glucocerebrosidase and reveal an allosteric binding site. J. Am. Chem. Soc. **140**, 5914–5924 (2018). 29676907 10.1021/jacs.7b13003 PMC6098685

[34] J. Benz , Novel β-glucocerebrosidase activators that bind to a new pocket at a dimer interface and induce dimerization. Angew. Chem. Int. Ed. Engl. **60**, 5436–5442 (2021). 33238058 10.1002/anie.202013890

[35] N. Palmer , Fragment-based discovery of a series of allosteric-binding site modulators of β-glucocerebrosidase. J. Med. Chem. **67**, 11168–11181 (2024). 38932616 10.1021/acs.jmedchem.4c00702

[36] J. M. Gruschus , Dissociation of glucocerebrosidase dimer in solution by its co-factor, saposin C. Biochem. Biophys. Res. Commun. **457**, 561–566 (2015). 25600808 10.1016/j.bbrc.2015.01.024 PMC4361889

[37] R. A. Steet , The iminosugar isofagomine increases the activity of N370S mutant acid beta-glucosidase in Gaucher fibroblasts by several mechanisms. Proc. Natl. Acad. Sci. U.S.A. **103**, 13813–13818 (2006). 16945909 10.1073/pnas.0605928103 PMC1564243

[38] Z. Yu, A. R. Sawkar, L. J. Whalen, C. H. Wong, J. W. Kelly, Isofagomine- and 2,5-anhydro-2,5-imino-D-glucitol-based glucocerebrosidase pharmacological chaperones for Gaucher disease intervention. J. Med. Chem. **50**, 94–100 (2007). 17201413 10.1021/jm060677i PMC2543937

[39] M. B. Tropak , Identification of pharmacological chaperones for Gaucher disease and characterization of their effects on beta-glucocerebrosidase by hydrogen/deuterium exchange mass spectrometry. ChemBioChem **9**, 2650–2662 (2008). 18972510 10.1002/cbic.200800304 PMC2910749

[40] W. Zheng , Three classes of glucocerebrosidase inhibitors identified by quantitative high-throughput screening are chaperone leads for Gaucher disease. Proc. Natl. Acad. Sci. U.S.A. **104**, 13192–13197 (2007). 17670938 10.1073/pnas.0705637104 PMC1936979

[41] J. J. Marugan , Evaluation of quinazoline analogues as glucocerebrosidase inhibitors with chaperone activity. J. Med. Chem. **54**, 1033–1058 (2011). 21250698 10.1021/jm1008902 PMC3103057

[42] J. M. Benito, J. M. García Fernández, C. Ortiz Mellet, Pharmacological chaperone therapy for Gaucher disease: A patent review. Expert Opin. Ther. Pat. **21**, 885–903 (2011). 21457079 10.1517/13543776.2011.569162

[43] G. H. Maegawa , Identification and characterization of ambroxol as an enzyme enhancement agent for Gaucher disease. J. Biol. Chem. **284**, 23502–23516 (2009). 19578116 10.1074/jbc.M109.012393 PMC2749124

[44] A. Narita , Ambroxol chaperone therapy for neuronopathic Gaucher disease: A pilot study. Ann. Clin. Transl. Neurol. **3**, 200–215 (2016). 27042680 10.1002/acn3.292 PMC4774255

[45] Y. M. Kim , Pharmacologic properties of high-dose ambroxol in four patients with Gaucher disease and myoclonic epilepsy. J. Med. Genet. **57**, 124–131 (2020). 31649052 10.1136/jmedgenet-2019-106132 PMC7029246

[46] D. Ysselstein , Evaluation of strategies for measuring lysosomal glucocerebrosidase activity. Mov. Disord. **36**, 2719–2730 (2021). 34613624 10.1002/mds.28815 PMC8853444

[47] J. Inglese , Quantitative high-throughput screening: A titration-based approach that efficiently identifies biological activities in large chemical libraries. Proc. Natl. Acad. Sci. U.S.A. **103**, 11473–11478 (2006). 16864780 10.1073/pnas.0604348103 PMC1518803

[48] E. Goldin , High throughput screening for small molecule therapy for Gaucher disease using patient tissue as the source of mutant glucocerebrosidase. PLoS ONE **7**, e29861 (2012). 22272254 10.1371/journal.pone.0029861 PMC3260169

[49] S. Patnaik , Discovery, structure-activity relationship, and biological evaluation of noninhibitory small molecule chaperones of glucocerebrosidase. J. Med. Chem. **55**, 5734–5748 (2012). 22646221 10.1021/jm300063b PMC3400126

[50] E. Aflaki , A new glucocerebrosidase chaperone reduces α-synuclein and glycolipid levels in iPSC-derived dopaminergic neurons from patients with Gaucher disease and Parkinsonism. J. Neurosci. **36**, 7441–7452 (2016). 27413154 10.1523/JNEUROSCI.0636-16.2016 PMC4945664

[51] E. Aflaki , Macrophage models of Gaucher disease for evaluating disease pathogenesis and candidate drugs. Sci. Transl. Med. **6**, 240ra273 (2014). 10.1126/scitranslmed.3008659 PMC4161206 24920659

[52] J. R. Mazzulli , Activation of β-glucocerebrosidase reduces pathological α-synuclein and restores lysosomal function in Parkinson’s patient midbrain neurons. J. Neurosci. **36**, 7693–7706 (2016). 27445146 10.1523/JNEUROSCI.0628-16.2016 PMC4951575

[53] M. K. Schwinn , CRISPR-mediated tagging of endogenous proteins with a luminescent peptide. ACS Chem. Biol. **13**, 467–474 (2018). 28892606 10.1021/acschembio.7b00549

[54] T. B. Kinder, P. K. Dranchak, J. Inglese, High-throughput screening to identify inhibitors of the type I interferon-major histocompatibility complex class I pathway in skeletal muscle. ACS Chem. Biol. **15**, 1974–1986 (2020). 32459468 10.1021/acschembio.0c00343 PMC7859889

[55] H. G. Larson , A genome-edited ERα-HiBiT fusion reporter cell line for the identification of ERα modulators via high-throughput screening and CETSA. Assay Drug. Dev. Technol. **19**, 539–549 (2021). 34662221 10.1089/adt.2021.059 PMC8713567

[56] S. Zhu , A fixable fluorescence-quenched substrate for quantitation of lysosomal glucocerebrosidase activity in both live and fixed cells. Angew. Chem. Int. Ed. Engl. **62**, e202309306 (2023), 10.1002/anie.202309306. 37582679

[57] T. Jong, A. Gehrlein, E. Sidransky, R. Jagasia, Y. Chen, Characterization of novel human β-glucocerebrosidase antibodies for Parkinson’s disease research. J. Parkinsons Dis. **14**, 65–78 (2024). 38251062 10.3233/JPD-230295 PMC10836542

[58] K. Fredriksen , Pathological α-syn aggregation is mediated by glycosphingolipid chain length and the physiological state of α-syn in vivo. Proc. Natl. Acad. Sci. U.S.A. **118**, e2108489118 (2021). 34893541 10.1073/pnas.2108489118 PMC8685670

[59] R. Huang , The NCGC pharmaceutical collection: A comprehensive resource of clinically approved drugs enabling repurposing and chemical genomics. Sci. Transl. Med. **3**, 80ps16 (2011). 10.1126/scitranslmed.3001862 PMC3098042 21525397

[60] M. I. Davis , Identification of novel *Plasmodium falciparum* hexokinase inhibitors with antiparasitic activity. Antimicrob. Agents Chemother. **60**, 6023–6033 (2016). 27458230 10.1128/AAC.00914-16 PMC5038330

[61] T. Cerbini , Transcription activator-like effector nuclease (TALEN)-mediated CLYBL targeting enables enhanced transgene expression and one-step generation of dual reporter human induced pluripotent stem cell (iPSC) and neural stem cell (NSC) lines. PLoS ONE **10**, e0116032 (2015). 25587899 10.1371/journal.pone.0116032 PMC4294658

[62] R. Tian , CRISPR interference-based platform for multimodal genetic screens in human iPSC-derived neurons. Neuron **104**, 239–255.e212 (2019). 31422865 10.1016/j.neuron.2019.07.014 PMC6813890

[63] D. Reczek , LIMP-2 is a receptor for lysosomal mannose-6-phosphate-independent targeting of beta-glucocerebrosidase. Cell **131**, 770–783 (2007). 18022370 10.1016/j.cell.2007.10.018

[64] E. Conzelmann, K. Sandhoff, Partial enzyme deficiencies: Residual activities and the development of neurological disorders. Dev. Neurosci. **6**, 58–71 (1983). 6421563 10.1159/000112332

[65] U. H. Schueler , Correlation between enzyme activity and substrate storage in a cell culture model system for Gaucher disease. J. Inherit. Metab. Dis. **27**, 649–658 (2004). 15669681 10.1023/b:boli.0000042959.44318.7c

[66] I. Bendikov-Bar, G. Maor, M. Filocamo, M. Horowitz, Ambroxol as a pharmacological chaperone for mutant glucocerebrosidase. Blood Cells Mol. Dis. **50**, 141–145 (2013). 23158495 10.1016/j.bcmd.2012.10.007 PMC3547170

[67] L. M. Jonsson , Biosynthesis and maturation of glucocerebrosidase in Gaucher fibroblasts. Eur. J. Biochem. **164**, 171–179 (1987). 3549301 10.1111/j.1432-1033.1987.tb11008.x

[68] C. Yang , Mutant glucocerebrosidase in Gaucher disease recruits Hsp27 to the Hsp90 chaperone complex for proteasomal degradation. Proc. Natl. Acad. Sci. U.S.A. **112**, 1137–1142 (2015). 25583479 10.1073/pnas.1424288112 PMC4313839

[69] F. Wang, W. Song, G. Brancati, L. Segatori, Inhibition of endoplasmic reticulum-associated degradation rescues native folding in loss of function protein misfolding diseases. J. Biol. Chem. **286**, 43454–43464 (2011). 22006919 10.1074/jbc.M111.274332 PMC3234808

[70] J. M. Aerts , Comparative study on glucocerebrosidase in spleens from patients with Gaucher disease. Biochem. J. **269**, 93–100 (1990). 2198026 10.1042/bj2690093 PMC1131536

[71] J. Lu , Histone deacetylase inhibitors prevent the degradation and restore the activity of glucocerebrosidase in Gaucher disease. Proc. Natl. Acad. Sci. U.S.A. **108**, 21200–21205 (2011). 22160715 10.1073/pnas.1119181109 PMC3248545

[72] C. Yang , Histone deacetylase inhibitors increase glucocerebrosidase activity in Gaucher disease by modulation of molecular chaperones. Proc. Natl. Acad. Sci. U.S.A. **110**, 966–971 (2013). 23277556 10.1073/pnas.1221046110 PMC3549125

[73] C. Sidrauski , Pharmacological brake-release of mRNA translation enhances cognitive memory. eLife **2**, e00498 (2013). 23741617 10.7554/eLife.00498 PMC3667625

[74] M. Ri , Identification of Toyocamycin, an agent cytotoxic for multiple myeloma cells, as a potent inhibitor of ER stress-induced XBP1 mRNA splicing. Blood Cancer J. **2**, e79 (2012). 22852048 10.1038/bcj.2012.26 PMC3408640

[75] J. M. den Heijer , A randomized single and multiple ascending dose study in healthy volunteers of LTI-291, a centrally penetrant glucocerebrosidase activator. Br. J. Clin. Pharmacol. **87**, 3561–3573 (2021). 33576113 10.1111/bcp.14772 PMC8451761

[76] J. M. den Heijer , A phase 1B Trial in GBA1-associated Parkinson’s disease of BIA-28-6156, a glucocerebrosidase activator. Mov. Disord. **38**, 1197–1208 (2023), 10.1002/mds.29346. 37195859

[77] C. Wang , Scalable production of iPSC-derived human neurons to identify tau-lowering compounds by high-content screening. Stem Cell Rep. **9**, 1221–1233 (2017). 10.1016/j.stemcr.2017.08.019 PMC5639430 28966121

[78] Y. Naito , Novel beta-glucocerebrosidase chaperone compounds identified from cell-based screening reduce pathologically accumulated glucosylsphingosine in iPS-derived neuronal cells. SLAS Discov. **28**, 344–349 (2023), 10.1016/j.slasd.2023.06.002. 37369311

[79] S. Rogers , “Discovery, SAR, and biological evaluation of non-inhibitory chaperones of glucocerebrosidase” in Probe Reports from the NIH Molecular Libraries Program (National Center for Biotechnology Information (US), Bethesda, MD, 2012). 23762943

[80] M. E. D. Schulze , Identification of ß-glucocerebrosidase activators for glucosylceramide hydrolysis. ChemMedChem **19**, e202300548 (2024), 10.1002/cmdc.202300548. 38381042

[81] E. Sidransky, G. Lopez, The link between the GBA gene and parkinsonism. Lancet Neurol. **11**, 986–998 (2012). 23079555 10.1016/S1474-4422(12)70190-4 PMC4141416

[82] K. E. Murphy , Reduced glucocerebrosidase is associated with increased α-synuclein in sporadic Parkinson’s disease. Brain **137**, 834–848 (2014). 24477431 10.1093/brain/awt367 PMC3927701

[83] J. Jian , Progranulin recruits HSP70 to β-glucocerebrosidase and is therapeutic against Gaucher disease. EBioMedicine **13**, 212–224 (2016). 27789271 10.1016/j.ebiom.2016.10.010 PMC5264254

[84] C. Blauwendraat , Genetic modifiers of risk and age at onset in GBA associated Parkinson’s disease and Lewy body dementia. Brain **143**, 234–248 (2020). 31755958 10.1093/brain/awz350 PMC6935749

[85] A. D. Klein , Identification of modifier genes in a mouse model of Gaucher disease. Cell Rep. **16**, 2546–2553 (2016). 27568557 10.1016/j.celrep.2016.07.085

[86] J. R. Simard , High-throughput quantitative assay technologies for accelerating the discovery and optimization of targeted protein degradation therapeutics. SLAS Discov. **26**, 503–517 (2021). 33430712 10.1177/2472555220985049

[87] M. K. Schwinn, L. S. Steffen, K. Zimmerman, K. V. Wood, T. Machleidt, A Simple and scalable strategy for analysis of endogenous protein dynamics. Sci. Rep. **10**, 8953 (2020). 32488146 10.1038/s41598-020-65832-1 PMC7265437

[88] H. Moriya, Quantitative nature of overexpression experiments. Mol. Biol. Cell **26**, 3932–3939 (2015). 26543202 10.1091/mbc.E15-07-0512 PMC4710226

[89] D. M. Hutt , Reduced histone deacetylase 7 activity restores function to misfolded CFTR in cystic fibrosis. Nat. Chem. Biol. **6**, 25–33 (2010). 19966789 10.1038/nchembio.275 PMC2901172

[90] N. H. Pipalia , Histone deacetylase inhibitor treatment dramatically reduces cholesterol accumulation in Niemann-Pick type C1 mutant human fibroblasts. Proc. Natl. Acad. Sci. U.S.A. **108**, 5620–5625 (2011). 21436030 10.1073/pnas.1014890108 PMC3078401

[91] O. Awad , Altered differentiation potential of Gaucher’s disease iPSC neuronal progenitors due to Wnt/β-catenin downregulation. Stem. Cell Rep. **9**, 1853–1867 (2017). 10.1016/j.stemcr.2017.10.029 PMC5785733 29198828

[92] L. M. Panicker , Gaucher disease iPSC-derived osteoblasts have developmental and lysosomal defects that impair bone matrix deposition. Hum. Mol. Genet. **27**, 811–822 (2018). 29301038 10.1093/hmg/ddx442 PMC6454561

[93] G. M. Riboldi , Transcriptome deregulation of peripheral monocytes and whole blood in GBA-related Parkinson’s disease. Mol. Neurodegener. **17**, 52 (2022). 35978378 10.1186/s13024-022-00554-8 PMC9386994

[94] C. Cretu , Structural basis of splicing modulation by antitumor macrolide compounds. Mol. Cell **70**, 265–273.e268 (2018). 29656923 10.1016/j.molcel.2018.03.011

[95] T. Schneider-Poetsch, J. K. Chhipi-Shrestha, M. Yoshida, Splicing modulators: On the way from nature to clinic. J. Antibiot. (Tokyo) **74**, 603–616 (2021). 34345042 10.1038/s41429-021-00450-1 PMC8472923

[96] K. S. Kim Guisbert, I. Mossiah, E. Guisbert, Titration of SF3B1 activity reveals distinct effects on the transcriptome and cell physiology. Int. J. Mol. Sci. **21**, 9641 (2020). 33348896 10.3390/ijms21249641 PMC7766730

[97] M. Costa-Mattioli, P. Walter, The integrated stress response: From mechanism to disease. Science **368**, eaat5314 (2020). 32327570 10.1126/science.aat5314 PMC8997189

[98] H. H. Rabouw , Small molecule ISRIB suppresses the integrated stress response within a defined window of activation. Proc. Natl. Acad. Sci. U.S.A. **116**, 2097–2102 (2019). 30674674 10.1073/pnas.1815767116 PMC6369741

[99] M. Halliday , Partial restoration of protein synthesis rates by the small molecule ISRIB prevents neurodegeneration without pancreatic toxicity. Cell Death Dis. **6**, e1672 (2015). 25741597 10.1038/cddis.2015.49 PMC4385927

[100] M. M. Oliveira , Correction of eIF2-dependent defects in brain protein synthesis, synaptic plasticity, and memory in mouse models of Alzheimer’s disease. Sci. Signal. **14**, eabc5429 (2021). 33531382 10.1126/scisignal.abc5429 PMC8317334

[101] H. G. M. Heijerman , Efficacy and safety of the elexacaftor plus tezacaftor plus ivacaftor combination regimen in people with cystic fibrosis homozygous for the F508del mutation: A double-blind, randomised, phase 3 trial. Lancet **394**, 1940–1948 (2019). 31679946 10.1016/S0140-6736(19)32597-8 PMC7571408

[102] L. A. Mathews Griner , High-throughput combinatorial screening identifies drugs that cooperate with ibrutinib to kill activated B-cell-like diffuse large B-cell lymphoma cells. Proc. Natl. Acad. Sci. U.S.A. **111**, 2349–2354 (2014). 24469833 10.1073/pnas.1311846111 PMC3926026

[103] G. L. Lin , Therapeutic strategies for diffuse midline glioma from high-throughput combination drug screening. Sci. Transl. Med. **11**, eaaw0064 (2019). 31748226 10.1126/scitranslmed.aaw0064 PMC7132630

[104] N. Dekker , Elevated plasma glucosylsphingosine in Gaucher disease: Relation to phenotype, storage cell markers, and therapeutic response. Blood **118**, e118–e127 (2011). 21868580 10.1182/blood-2011-05-352971 PMC3685900

[105] M. J. Ferraz , Lysosomal glycosphingolipid catabolism by acid ceramidase: Formation of glycosphingoid bases during deficiency of glycosidases. FEBS Lett. **590**, 716–725 (2016). 26898341 10.1002/1873-3468.12104

[106] D. Elstein , Reductions in glucosylsphingosine (lyso-Gb1) in treatment-naïve and previously treated patients receiving velaglucerase alfa for type 1 Gaucher disease: Data from phase 3 clinical trials. Mol. Genet. Metab. **122**, 113–120 (2017). 28851512 10.1016/j.ymgme.2017.08.005

[107] U. H. Schueler , Toxicity of glucosylsphingosine (glucopsychosine) to cultured neuronal cells: A model system for assessing neuronal damage in Gaucher disease type 2 and 3. Neurobiol. Dis. **14**, 595–601 (2003). 14678774 10.1016/j.nbd.2003.08.016

[108] L. T. Lelieveld , Consequences of excessive glucosylsphingosine in glucocerebrosidase-deficient zebrafish. J. Lipid. Res. **63**, 100199 (2022). 35315333 10.1016/j.jlr.2022.100199 PMC9058576

[109] K. Stahl-Meyer , Galactosyl- and glucosylsphingosine induce lysosomal membrane permeabilization and cell death in cancer cells. PLoS ONE **17**, e0277058 (2022). 36409725 10.1371/journal.pone.0277058 PMC9678304

[110] O. Nilsson, L. Svennerholm, Accumulation of glucosylceramide and glucosylsphingosine (psychosine) in cerebrum and cerebellum in infantile and juvenile Gaucher disease. J. Neurochem. **39**, 709–718 (1982). 7097276 10.1111/j.1471-4159.1982.tb07950.x

[111] H. Braunstein , UPR activation and CHOP mediated induction of GBA1 transcription in Gaucher disease. Blood Cells Mol. Dis. **68**, 21–29 (2018). 27856178 10.1016/j.bcmd.2016.10.025

[112] J. Magalhaes, M. E. Gegg, A. Migdalska-Richards, A. H. Schapira, Effects of ambroxol on the autophagy-lysosome pathway and mitochondria in primary cortical neurons. Sci. Rep. **8**, 1385 (2018). 29362387 10.1038/s41598-018-19479-8 PMC5780491

[113] T. Logan , Rescue of a lysosomal storage disorder caused by Grn loss of function with a brain penetrant progranulin biologic. Cell **184**, 4651–4668.e4625 (2021). 34450028 10.1016/j.cell.2021.08.002 PMC8489356

[114] M. J. Simon, T. Logan, S. L. DeVos, G. Di Paolo, Lysosomal functions of progranulin and implications for treatment of frontotemporal dementia. Trends Cell Biol. **33**, 324–339 (2023). 36244875 10.1016/j.tcb.2022.09.006

