# Supplementary material for: High-throughput screening for small-molecule stabilizers of misfolded glucocerebrosidase in Gaucher disease and Parkinson’s disease
Source: Proc Natl Acad Sci U S A. 2024 Oct 10;121(42):e2406009121. doi: 10.1073/pnas.2406009121 (PMC11494340; doi:10.1073/pnas.2406009121)
Supplement: Supplementary file 1 — Appendix 01 (PDF) [file pnas.2406009121.sapp.pdf]

PNAS

Supporting Information for

High-throughput screening for small-molecule stabilizers of misfolded glucocerebrosidase in Gaucher disease and Parkinson's disease

Darian Williams[#1], Logan M. Glasstetter[#2], Tiffany T. Jong[#2], Tiffany Chen[2], Abhijeet Kapoor[1], Sha Zhu[3], Yanping Zhu[3], Raul Calvo[1], Alexandra Gehrlein[4], Kimberly Wong[2], Andrew N. Hogan[2], David J. Vocadlo[3], Ravi Jagasia[*4], Juan J. Marugan[*1], Ellen Sidransky[*2], Mark J. Henderson[*1], Yu Chen[*2]

[1]Division of Preclinical Innovation, National Center for Advancing Translational Sciences, National Institutes of Health, Rockville, MD 20850.

[2]Molecular Neurogenetics Section, Medical Genetics Branch, National Human Genome Research Institute, National Institutes of Health, Bethesda, MD 20892.

[3]Department of Chemistry and Department of Molecular Biology and Biochemistry, Simon Fraser University, Burnaby, BC, V5A 1S6, Canada.

[4]Roche Pharma Research and Early Development, Neuroscience and Rare Diseases Discovery and Translational Area, Roche Innovation Center Basel, 4070 Basel, Switzerland.

[#]These co-first authors contributed equally (D.W., L.M.G., T.T.J.).

*These co-corresponding authors contributed equally (ravi.jagasia@roche.com; maruganj@mail.nih.gov; ellen.sidransky@nih.gov; mark.henderson2@nih.gov; yu.chen@nih.gov).

This PDF file includes:

Materials and Methods

Figures S1 to S17

References

Materials and Methods

Plasmid construction

The *CLYBL*-targeting pC13N-iCAG.copGFP vector (Addgene #66578) [1] was used as a backbone to generate three constructs encoding different N-terminal-HiBiT-*GBA1* variants (WT, N370S, L444P) under the control of a constitutive chimeric CAG promoter. A custom gene block (GENEWIZ) encoding the *GBA1* signaling peptide, HiBiT tag, and Gly/Ser (GS) linker was inserted into the pC13N-iCAG.copGFP vector through BsrGI and MluI digestion and Hi-T4™ ligation (Cat.#M2622; New England Biolabs), thus replacing copGFP and generating the pC13N_N-HiBiT plasmid. *GBA1* transgenes were PCR-amplified from in-house cDNA (using the following 5' to 3' primer sequences: Forward: TGGGAATTCTGGTGGAGGATCCgcccgcccctgc; Reverse: acctgaggagtgaattcacgcgttcactggcgacgccac) with Q5® Hot Start High-Fidelity master mix (Cat.#M0494, New England Biolabs) and purified by DNA gel electrophoresis. The pC13N_N-HiBiT vector was prepared to receive the transgene inserts through BamHI and MluI digestion. The linearized vector was assembled with the transgene amplicons via NEBuilder® HiFi DNA Assembly (Cat.#E5520; New England Biolabs), thus generating the three pC13N_N-HiBiT-*GBA1* plasmids. The three ligation products were separately transformed into One Shot™ TOP10 Chemically Competent *E. coli* (Cat.#C404010; Invitrogen), which underwent selection with Kanamycin. A single colony from each plate was then grown in liquid bacterial culture with Kanamycin; the plasmid DNA was amplified using a NucleoBond Xtra Midi kit (Cat.#740410; Macherey-Nagel) and the sequence was verified with Sanger sequencing (GENEWIZ).

H4 cell culture and transfection

The H4 human neuroglioma cell line (HTB-148) was obtained from American Type Culture Collection (ATCC). An H4 *GBA1* loss-of-function (*GBA1*-KO) clonal cell line was generated through zinc-finger nuclease-mediated targeted integration of gene disruption cassettes into exon 4 of *GBA1* alleles, as previously described [2]. H4 cells were cultured in DMEM + GlutaMAX™ with high glucose (Cat.#10566-016; Gibco) supplemented with 10% heat-inactivated fetal bovine serum and 1 mM sodium pyruvate (Cat.#11360-070; Gibco), with or without 100 U/mL Penicillin-Streptomycin (Cat.#15140-122; Gibco). Cell cultures were maintained in a humidified incubator (37°C, 5% CO_2) and routinely tested negative for mycoplasma contamination.

HiBiT-*GBA1* constructs were transfected into the H4 *GBA1*-KO cell line using Lipofectamine™ 3000 (Cat.#L3000001; Invitrogen), following the manufacturer's protocol, with Opti-MEM™ Reduced Serum Medium (Cat.#31985062; Gibco) used for dilution.

Stable integration of HiBiT-GBA1 transgene into H4 GBA1-knockout cell line

HiBiT-*GBA1* transgenes were stably incorporated into the H4 *GBA1*-KO line using TALEN-enhanced integrative gene transfer [1, 3]. Briefly, *GBA1*-KO H4 cells were seeded at an initial density of 800,000 cells/well in a 6-well plate. After 1 day, the cells were transfected with one of three pC13N_N-HiBiT-*GBA1* donor plasmids (WT, N370S, or L444P; 750 ng/well), along with left and right TALENs (pZT-C13-L1: Addgene #62196; pZT-C13-R1: Addgene #62197; 375 ng/well each) targeting the human Citrate Lyase Beta-Like (*CLYBL)* intragenic safe-harbor locus, which is located in intron 2 (between exons 2 and 3). Lipofectamine™ 3000 (Cat.#L3000001; Invitrogen) was used as the transfection reagent, following the manufacturer's protocol, with Opti-MEM™ Reduced Serum Medium (Cat.#31985062; Gibco) used for dilution.

After 24 h, selection with Geneticin™ (600 ng/µL; G418 sulfate; Cat.#10131027; Gibco) was performed for 1 week to enrich for H4 cells expressing the splicing acceptor – T2A self-cleaving peptide (SA-T2A)-linked Neomycin resistance cassette (NeoR/KanR). With this approach, expression of the resistance gene is driven by the active endogenous *CYLBL* promoter; thus, the antibiotic selection enriches for targeted – as opposed to random – integration events [1, 4]. Following antibiotic selection, single clones with stable integration of the HiBiT-*GBA1* construct were isolated via fluorescence-activated cell sorting into a 96-well plate. Individual surviving clones were then expanded, and selected clones underwent quality control via Sanger sequencing (GENEWIZ) and copy number determination.

Copy number determination assay

The Droplet Digital polymerase chain reaction (ddPCR) copy number determination assay [5, 6] was performed on a QX200 ddPCR system (Bio-Rad Laboratories) at the Center for Cancer Research (CCR) Genomics Core facility (National Cancer Institute, Bethesda, MD). For sample preparation, a ddPCR reaction mix was prepared which consisted of 1.1 µL of 20X FAM-conjugated, HiBiT-directed target probe (Unique Assay ID: dCNS206145924; MIQE Context: ACAGGATTGCTTCTACTTCAGGCAGTGTCGTGGGCATCAGGTATGGTGAGCGGCTGGCGG CTGTTCAAGAAGATTAGCGGGAGCTCCGGTGGCTCGAGCGGTGGGAATTCTGGTGGAGGA TCC), 1.1 µL of 20X HEX-conjugated reference probe (RPP30; Unique Assay ID: dHsaCP1000485; MIQE Context: ATGAGGAACCTGAAACTTCATGTTAAGTAACTTGTAAGTGGTAGTGCATAGACTTTAAATCAG GCAGACTGACACTAGAGTTCACATTCATAACCACTCCTCAAATGTCCTCCTACTCTTGAC), 1.1 µL of HindIII-HF (Cat.#R3104S; New England Biolabs) diluted with CutSmart® Buffer (Cat.#B7204S; New England Biolabs), 7.7 µL (50 ng) of DNA sample, and 11 µL of 2X ddPCR Supermix for Probes (No dUTP). A plate loaded with 22 µL/well of total sample mixture was placed in an Automated Droplet Generator, after which it was heat sealed with a foil sheet and transferred to a C1000 Touch™ Thermal Cycler. PCR was conducted with a ramp rate of 2°C/s for 1 cycle of enzyme activation (95°C for 10 min), 40 cycles of denaturation (94°C for 30 sec) and annealing/extension (60°C for 1 min), and 1 cycle of enzyme deactivation (98°C for 10 min), after which samples were held at 4°C. The plate was read on a QX200 Droplet Reader, and data were analyzed with QuantaSoft Analysis Pro software (Bio-Rad Laboratories). The results of the ddPCR copy number determination assay informed selection of clones with stable integration of 1 copy of HiBiT-*GBA1*, which was indicated by a FAM/HEX ratio of ~0.5.

LIMP-2 knockdown in H4 cells

The gRNA targeting LIMP-2 (acagttgacgaattgctctg) was synthesized by Integrated DNA Technologies and cloned into pCAG-eCas9-GFP-U6-gRNA, an all-in-one CRISPR/Cas9 vector with high-fidelity eSpCas9, to generate pCAG-eCas9-GFP-U6-LIMP2_gRNA. pCAG-eCas9-GFP-U6-gRNA was a gift from Jizhong Zou (Addgene plasmid # 79145; http://n2t.net/addgene:79145; RRID:Addgene_79145). pCAG-eCas9-GFP-U6-LIMP2_gRNA was transfected into *GBA1*-WT and HiBiT-hGCase-WT H4 cells. GFP+ *GBA1*-WT and HiBiT-hGCase-WT H4 cells were enriched by FACS sorting and used for Western blot analysis of GCase and LIMP-2.

Western blotting

Cell pellets were lysed in 1% Triton X-100 lysis buffer [1% Triton X-100; 10% glycerol; 150 mM NaCl; 20 mM HEPES pH 7.4; 1 mM EDTA; 1.5 mM $MgCl_2$] supplemented with tablets of cOmplete™, Mini, EDTA-free Protease Inhibitor Cocktail (Cat.#11836170001; 1 tablet/10 mL) and PhosSTOP™ Phosphatase Inhibitor Cocktail (Cat.#04906837001; 1 tablet/10 mL). Protein was extracted through pipetting, three 10-sec pulsed bath sonication steps (S-4000, Misonix), and three freeze-thaw cycles. Thereafter, the lysates were centrifuged for 15 min (21,000 x *g*; 4°C), and the supernatant containing extracted protein was collected. Protein concentration was evaluated using a Pierce™ BCA Protein Assay Kit (Cat.#23225; Thermo Scientific).

Protein lysates were boiled in loading buffer [1:9 ratio of 2-mercaptoethanol:4x Laemmli Sample Buffer (Cat.#1610747; Bio-Rad Laboratories)] for 10 min. Proteins were loaded at 17 – 20 µg/lane on a 4–20% midi Criterion™ TGX Stain-Free™ precast polyacrylamide gel (Cat.#5678094; Bio-Rad Laboratories), resolved via gel electrophoresis, and transferred onto a polyvinylidene difluoride (PVDF) membrane via the Trans-Blot® Turbo™ Transfer System (Bio-Rad Laboratories). The membrane was dried, reactivated with methanol, rinsed with water and TBST (1X TBS; 0.1% Tween), and blocked for 1 h at RT [in 1X TBST; 5% (w/v) BSA (Cat.#A-7888; Sigma-Aldrich)]. The membrane was probed overnight (4°C) with primary antibody [in 1X TBST with 2% (w/v) BSA]. Thereafter, it was washed with TBST (3 x 10 min) at RT, incubated with secondary antibody [in 1X TBST with 2% (w/v) BSA], and washed again with TBST (3 x 10 min, RT). The blot was visualized with SuperSignal West Pico PLUS Chemiluminescent Substrate (Cat.#34580; Thermo Scientific) using the ChemiDoc MP Imaging System (Bio-Rad Laboratories). For loading control, total protein was captured via Stain-Free™ gel imaging or Coomassie staining [SimplyBlue™ SafeStain (Cat.#LC6060; Life Technologies)]. Stain-Free™ gels were imaged after electrophoresis and prior to transfer using the stain-free gel setting on the ChemiDoc MP Imaging System. Coomassie was stained on membranes following a 30-minute incubation (37 °C) in Restore™ PLUS stripping buffer (Cat.#46430X4; Thermo Scientific) and thorough drying. Membranes were incubated at RT with Coomassie stain for 10 minutes or until satisfactory staining was achieved, rinsed with methanol and water, then imaged with either the colorimetric or PonceauS settings on the ChemiDoc MP Imaging System.

The following anti-GBA1 primary antibodies were used: 2E2 (mouse monoclonal; Cat.#H00002629-M01; Abnova; 1:2,000) and R386 (rabbit polyclonal; in-house; 1:10,000). LIMP-2 was detected with the EPR26243-125 rabbit monoclonal antibody (Cat.#ab314217; Abcam;1:1,000). The following goat Horseradish Peroxidase (HRP)-coupled secondary antibodies were used: anti-mouse IgG (Cat.#ab205719; Abcam; 1:10,000) and anti-rabbit IgG (Cat.#ab205718; Abcam; 1:2,000 – 1:20,000).

Glycosylation analysis (Endo H and PNGase F assay)

Glycosidase sensitivity analysis was performed as previously described with some modifications [7]. Briefly, H4 cell pellets were lysed in 1% Triton X-100 lysis buffer supplemented with Pierce™ Protease and Phosphatase Inhibitor Mini Tablets (1 tablet/10 mL; Cat.#A32959)]. The cells were sonicated for 10 quick pulses using a Sonic Dismembrator Model D100 (Fisher Scientific), left standing for 15-30 min on ice, and then centrifuged for 15 min (13,000 x *g*; 4°C). The supernatant containing lysate was collected and stored at -80°C until digestion.

For each condition, 40 – 50 µg of protein lysate from each sample was denatured in 1X Glycoprotein Denaturing Buffer (Cat.#B1704S; New England Biolabs) with 20 µL total volume at 100°C for 10 min. The denatured protein lysates were digested with glycosidase enzymes, Endo

H_f (Cat.#P0703S; New England Biolabs) or PNGase F (Cat.#P0708L; New England Biolabs). For Endo H_f digestion, 20 µL of denatured protein lysate was combined with 2.5 µL of 10X GlycoBuffer 3 (Cat.#B1720S; New England Biolabs) and 2.5 µL of Endo H_f in a total reaction volume of 25 µL. For PNGase F digestion, 20 µL of denatured protein lysate was combined with 2.6 µL of 10X GlycoBuffer 2 (Cat.#B3704S; New England Biolabs), 2.6 µL of 10% NP-40 (Cat.#B2704S; New England Biolabs), and 1.5 µL of PNGase F in a total reaction volume of 26.7 µL. Undigested control samples were prepared by combining 20 µL of denatured protein lysate with 5 µL of water. The reactions were incubated at 37°C for 1.5 h, after which Western blots were run to examine the digestions. The Endo H-sensitive fraction was designated as immature, ER-retained GCase, while the Endo H-resistant fraction was considered post-ER-localized GCase [7, 8]. Both fractions are responsive to PNGase F treatment.

AlphaLISA

Amplified Luminescent Proximity Homogeneous Assay (AlphaLISA) was performed using mouse monoclonal antibodies hGCase-1/17 and hGCase-1/23, as previously described [2]. Briefly, $1x10^6$ H4 cells were washed once with PBS and lysed in 80 µL of GCase lysis buffer (0.05 M citric acid, 0.05 M KH_2PO_4, 0.05 M K_2HPO_4, 0.11 M KCl, 0.01 M NaCl, 0.001 M $MgCl_2$, pH 6.0 with 0.1% (v/v) Triton X-100, supplemented with freshly added protease inhibitor). Samples were diluted 1/5 or 1/10 in 1X Immunoassay buffer (Cat.#AL000F; PerkinElmer). For calibration, a standard curve ranging from 2.4 pM – 1,250 pM of imiglucerase (Genzyme) was generated through 2x serial dilution. 10 µL of samples or standards were incubated for 4 h at RT in the dark with 1 nM of biotinylated hGCase-1/23 and 20 µg/mL of hGCase-1/17-conjugated Acceptor beads (50:1, beads:antibody). Thereafter, 40 µg/mL of AlphaScreen Streptavidin Donor beads (Cat.#6760002; PerkinElmer) were added and incubated for 1 h at RT in the dark. The assay was performed in a flat white, 384-well OptiPlate (Cat.#6007290, PerkinElmer), which was read on a Tecan Spark plate reader (excitation: 680 nm; emission: 520–620 nm). GCase concentration was calculated based on a sigmoidal non-linear regression of the imiglucerase standard curve and then normalized to total protein concentration.

GCase activity assay (4-MUG)

Enzymatic activity of GCase was determined based on cleavage of the fluorogenic substrate 4-methylumbelliferyl-β-D-glucopyranoside (4-MUG) [9]. Briefly, freshly-prepared GCase buffer [McIlvaine buffer (0.2 M Na_2HPO_4 and 0.1 M citric acid titrated to pH 5.4); cOmplete™, Mini, EDTA-free Protease Inhibitor Cocktail (Cat.#11836170001; 1 tablet/10 mL); 0.25% (v/v) Triton X-100 (Cat.#T9284; Sigma-Aldrich)] was activated with 0.2% (w/v) sodium taurocholate (Cat.#86339; Sigma-Aldrich). Protein was extracted from H4 cell pellets in GCase buffer through pipetting, at least two 10-sec pulsed bath sonication steps (S-4000, Misonix), and 3 freeze-thaw cycles. Thereafter, the lysates were centrifuged for 15 min (21,000 x *g*; 4°C), and the supernatant containing extracted protein was collected. Protein concentration was measured [Pierce™ BCA Protein Assay Kit (Cat.#23225; Thermo Scientific)], and samples were diluted to 1 µg/µL, unless specified otherwise. Final protein concentration was verified by a second BCA assay and used for data normalization.

The 4-MUG GCase activity assay was conducted in a Greiner black 384-well plate (Cat.#781209), to which protein samples were added (10 µL/well) and further diluted with GCase buffer (5 µL/well). The plate was sealed, centrifuged, and incubated (37°C) in a shaking

plate incubator at 600 rpm for 15 min. After brief centrifugation, 15 µL of assay buffer (2.5 mM 4-MUG; 0.25% v/v DMF in GCase buffer) was added to each assay well. The plate was sealed, centrifuged, and incubated (37°C) for 60 min, shaking at 450 rpm. After incubation, the plate was centrifuged, and 30 µL of 1 M glycine stop solution (pH 10.5) was added to each well. Fluorescence was top read on a FlexStation® 3 Multi-Mode Microplate Reader (Molecular Devices; 365 nm excitation wavelength; 449 nm emission wavelength; 435 nm cutoff; 6 reads/well). For H4 cells, relative GCase activity was calculated by adjusting for protein concentration, correcting for *GBA1*-KO H4 cell background, and normalizing to *GBA1*-WT or HiBiT-WT signal.

Immunocytochemistry

For studies of GCase colocalization with LAMP1 or LIMP-2, H4 cells were seeded into 8-well imaging chambers at a density of 30,000 cells/chamber, 48 h prior to immunostaining. For high-content screening with small molecules, H4 cells were seeded into 96-well, black, optically clear, flat-bottom, tissue-culture treated PhenoPlates (PerkinElmer) at 5,000 cells/well in 200 µL media and treated at ~50% confluence.

For immunostaining [2], cells were washed once with PBS (pH 7.4) and fixed with 4% paraformaldehyde (20 or 30 min, RT). Fixed cells were washed with PBS (3 x 5 min) and incubated overnight (4°C) with primary antibody in ICC antibody diluent [PBS containing 0.05% or 0.1% saponin (filtered) and 1% BSA]. After primary antibody incubation, cells were washed with PBS (3 x 5 min, at least) and incubated for 1 h (RT) with secondary antibody in antibody diluent. After secondary antibody incubation, cells were washed with PBS (3 x 5 min, at least) and underwent nuclear staining. For Hoechst staining, Hoechst 33342 solution (Cat.#62249; ThermoFisher Scientific) was diluted in PBS to 5 µg/mL and added during the third wash. For DAPI staining, cells were stored at 4°C in PBS with NucBlue™ Fixed Cell ReadyProbes™ Reagent (DAPI) (Cat.#R37606; ThermoFisher Scientific) until imaging. Imaging plates were sealed with a heat-resistant film (Cat.#89496-565; VWR International).

The following primary antibodies were used: GCase (5 µg/mL; hGCase-1/23; mouse monoclonal; Roche [2]); LAMP1 (1:500; D2D11, rabbit monoclonal; Cat.#9091; Cell Signaling Technology); LIMPII (1:200; EPR26243-125, rabbit monoclonal; Cat.#ab314217; Abcam). The following secondary antibodies were used: Rhodamine Red™-X (RRX) AffiniPure Donkey Anti-Rabbit IgG (H+L) (1:200; Cat.#711-295-152; Jackson ImmunoResearch Laboratories, Inc.); Alexa Fluor® 488 AffiniPure Donkey Anti-Mouse IgG (H+L) (1:400; Cat.#715-545-150; Jackson ImmunoResearch Laboratories, Inc.).

Confocal images were acquired with a Zeiss LSM 880 confocal microscope and Airyscan images with a Zeiss LSM 980 confocal microscope, both using a 63x oil objective. Pearson's correlation coefficient was calculated using Fiji ImageJ Colocalization. High-content imaging was conducted on a PerkinElmer Opera Phenix Plus confocal high-content screening system; images were captured in 27 fields per well with a 63x water, NA 1.15 objective. Image analysis was performed using PerkinElmer Columbus software and algorithms to quantify the sum per well of mean GCase intensity (Alexa 488 channel) in spots of LAMP1 (TRITC channel), which was normalized to number of nuclei (DAPI channel).

Lipidomic analysis

For initial analysis of glucosylsphingosine (GluSph) levels in frozen pellets of untreated H4 cells, cells were lysed by addition of distilled water followed by protein precipitation with methanol, as described [2, 10]. The dry lipid samples were reconstituted in a mixture of acetonitrile/water 90/10 (v/v) containing 1% DMSO. Analysis was performed via positive ion electrospray LC-MS/MS in multiple reaction-monitoring (MRM) mode, using deuterated compounds as internal standards. A Waters Xevo-TQ-S mass spectrometer connected to a complete Waters Acquity I-class UPLC system was used as previously described, with a mixture of acetonitrile/methanol/water 40/40/20 (v/v/v) as the wash solvent [2, 10]. Samples were analyzed on a BEH glycan amide column (100 × 2.1 mm, 1.7 µm particle size; Waters Corporation, Switzerland) with a flow rate of 0.25 mL/min and an oven temperature of 30°C. Eluent A consisted of 100 mM ammonium acetate, while eluent B was acetonitrile. Glycospecific separation was achieved by isocratic elution with 90% eluent B, followed by a washing step with 10% eluent B and column reconditioning. Each sample contained $1x10^6$ cells. Lipid levels were calculated by linear regression, using the peak area ratio of analyte to internal standard as the response, and expressed as pmol/million cells.

For other studies, lipid levels were determined by supercritical fluid chromatography (SFC) separation coupled with tandem mass spectrometry (MS/MS) detection [9, 11, 12]. Lipid extraction and SFC-MS/MS analysis were performed by the Analytical Unit of the Lipidomics Shared Resource at the Medical University of South Carolina (MUSC) Hollings Cancer Center. As an endpoint in the drug discovery pipeline, lipids were extracted from frozen cell pellets of compound- or DMSO-treated H4 cells, and levels of GluSph were determined. Levels of various species of glucosylceramide (GluCer) in frozen cell pellets of untreated H4 cells ($2x10^6$ cells per sample) were also evaluated. Cell pellets were washed with 0.15 M NaCl solution (Cat.#46-032-CV; Corning) and stored at -80°C prior to analysis. Extracted lipid samples and synthetic standards were processed on a setup comprising a Waters Acquity SFC/UPC2 Chromatography System and TSQ Quantum Access Max Triple Quadrupole Mass Spectrometers (Thermo Scientific) operating in positive MRM mode and employing a gradient elution. Quantitation required the generation of analyte-specific eight-point calibration curves (analyte/internal standard peak area ratio versus analyte concentration), as described previously [13]. Lipid measurements [pmol/total sample] were normalized to levels of inorganic phosphate [nmol] in each sample, as determined by Bligh & Dyer (B&D) re-extraction [14] of an aliquot from the original total sample extract.

Microscale thermophoresis

Microscale thermophoresis (MST) experiments were performed using the Monolith NT.Automated instrument (NanoTemper Technologies). Briefly, enzymatically-active recombinant human GCase with a C-terminal 6-His tag (Cat.#7410-GHB-020; Bio-Techne) was purchased as a 0.2 µm filtered solution in 50 mM sodium citrate, pH 5.5 at a concentration of 0.319 mg/mL. The recombinant GCase was further diluted with assay buffer (50 mM sodium citrate, pH 5.5) to 0.160 mg/mL and incubated (RT) for 30 min with 50 nM RED-tris-NTA 2nd Generation dye from the Monolith His-Tag Labeling Kit (Cat.#MO-L018; NanoTemper Technologies). The labeling reaction mixture was centrifuged for 10 min at 4°C and 15,000 x *g* to remove protein aggregates, after which the supernatant was diluted 2.5x with assay buffer. For each compound measured, a 12-point, 2x titration series of compound in DMSO was prepared; 1 µL of compound in DMSO from each point in the titration series was combined with 9 µL of assay buffer and 10 µL of labeled GCase in a 384-well plate, which was left standing for 30 min (RT). The resultant final concentration of labeled GCase was ~570 nM (in 5% DMSO). MST measurements were conducted with medium MST power, 15% excitation power, red (Pico)

excitation color, and 1.5-sec on time using Monolith NT.Automated capillary chips (Cat.#MO-AK002). Data analysis was performed using MO.Affinity Analysis v2.3 software (NanoTemper Technologies), with data fit to a K_d model.

Miniaturization and high-throughput screening of HiBiT-GCase and CellTiter-Glo assays

H4 cells expressing HiBiT-GCase (WT, N370S, or L444P) were grown to 70% confluency and seeded into 1536-well solid white TC-treated microplates (Cat.#782073, Greiner) at a density of 2,000 cells/well in 5 µL media, covered with stainless steel cell culture lids, and incubated for 24 h at 37°C and 5% CO_2. Cells were then treated with 50 nL of compounds or vehicle (DMSO) via acoustic dispensing (Echo, Beckman Coulter) and incubated for 24 h at 37°C and 5% CO_2. Assessment of total cellular HiBiT-GCase protein abundance was performed using the Nano-Glo® HiBiT Lytic Detection System (Cat.#N3040, Promega), according to the manufacturer's protocol. Briefly, the LgBiT Protein (1:100) and the Nano-Glo® HiBiT Lytic Substrate (1:50) were diluted into RT Nano-Glo® HiBiT Lytic Buffer and added at a 1:1 ratio with cell culture media. Cells were incubated with the HiBiT lytic mixture for 10 minutes at RT, and luminescence was measured on a PHERAstar FSX microplate reader with a LUM plus optical module (3600 gain). Settling time and measurement time were selected for optimal precision measurement over speed. Data analysis was performed by comparing the fold change in HiBiT luminescence in compound-treated cells versus vehicle-treated cells. Assessment of cell viability was performed using Promega CellTiter-Glo® Luminescent Cell Viability Assay according to the manufacturer's recommendation. Briefly, CellTiter-Glo was added at a 1:1 ratio in replicate assay plates with cell culture media. Cells were incubated with the CellTiter-Glo buffer for 10 minutes at RT, and luminescence was measured on a PHERAstar FSX microplate reader using the same settings as the HiBiT assay.

Primary screen hit selection

Primary screen hits were determined based on a system of curve classes [15], which provides a heuristic measure of data confidence. The amended qHTS curve classification system (CC-v2) was utilized [16]. Curve classes of 1 are complete curves containing two asymptotes and an inflection point, with data confidence decreasing from curve class 1.1 to 1.4. Curve classes of 2 are incomplete curves containing one asymptote and an inflection point, with data confidence decreasing from curve class 2.1 to 2.4. Compounds with a curve class of 5 are inconclusive and were included, so as to not lose any potential hits that could not be classified. For the primary screens, all compounds from CC-v2 1.1-1.4, 2.1-2.4, and 5 were included; further hit selection was applied based on an efficacy cutoff of 40%, and compounds that showed efficacy at a single concentration while also displaying inhibitory effects at a majority of other concentrations were removed (formally defined as an AUC default cutoff of -50%). For the follow-up screens, data were included for all compounds irrespective of curve class, and hit selection was applied using the above cutoffs.

Target profile and pathway analysis of hit compounds

To gain insight into possible mechanisms of actions for the 140 confirmed follow-up hits, we first built a protein-target profile for each hit compound by collecting target annotations from the NCATS in-house database as well as by querying human target proteins for each compound from five public repositories: ChEMBL [17], DrugBank [18], IUPHAR [19], PharmGKB [20], and

PubChem [21]. A bioactivity cutoff of 10 μM or less was used when querying targets from PubChem, ChEMBL, and IUPHAR. Of the 140 hits, target annotations were obtained for 83 compounds while the remaining 57 could not be resolved. Each hit was annotated with at least one target protein (represented by their UniProt accession number), and collectively these 83 hits mapped to 274 unique target proteins, as many compounds target multiple proteins (hit-compounds-targets profile). A protein-target profile was also built for the entire collection of primary screening compounds (background-compounds-targets profile) which collectively mapped to 2773 unique target proteins. Fisher's exact test was used to conduct a statistical overrepresentation analysis to identify targets that were over-represented in the hit-compounds-targets profile compared to background. The resulting *p*-value from the Fisher's exact test was corrected via the false discovery rate (FDR) using the Benjamini–Hochberg method. Applying an FDR cutoff of 5% resulted in 30 enriched targets. The known and predicted interaction network of these targets was determined using the STRING database [22]. The UniProt accession number of the enriched targets was then used as input to the Reactome pathway database [23] to identify pathways that are overrepresented by these targets, revealing potential pathways affected by hit compounds.

Miniaturization and high-throughput screening of LysoFix-GBA secondary assay

H4 cells expressing HiBiT-GCase-L444P were grown to 70% confluency and seeded into 384-well black, optically clear flat-bottom, tissue-culture treated PhenoPlates (PerkinElmer) at a concentration of 25,000 cells/well in 40 μL Fluorobrite media for 24 h at 37°C and 5% CO_2. Cells were then treated with 50 nL of compounds or vehicle (DMSO) via acoustic dispensing and incubated for either 24 h or 72 h at 37°C and 5% CO_2. To assess lysosomal GCase activity, LysoFix-GBA was synthesized as previously described [24] and resuspended in DMSO to a stock concentration of 10 mM. Working stocks were prepared by diluting LysoFix-GBA in DMSO. Cells were then treated to desired concentration with 50 nL of the LysoFix-GBA working stock via acoustic dispensing and either imaged immediately for live-cell imaging at 37°C and 5% CO_2 or incubated for 2 h at 37°C and 5% CO_2 and imaged after adding Hoechst-33342 prepared in Fluorobrite media (1 μg/mL) for 15 min. Imaging was performed on a PerkinElmer Opera Phenix Plus confocal high-content screening system. Image analysis was performed using PerkinElmer Columbus software and algorithms to quantify the number, intensity, and area of LysoFix-GBA spots per cell indicated by nuclei number. The data were then represented as fold change of integrated LysoFix-GBA spot intensity per cell in compound-treated cells versus vehicle-treated cells. Presented images are contrasted to the highest intensity condition within each experiment.

Activator assays

Recombinant GCase-WT (imiglucerase) was sourced from residual solution remaining after clinical infusions of imiglucerase (Cerezyme®, Genzyme Corporation; activity: 14 units/mL; specific activity: 42.2 units/mg; Mr = 60,430) [25]. Glycerol was added to the enzyme solution to 50%, and small aliquots were stored at -80°C. Following enzyme concentration-response analysis, imiglucerase, along with compounds or vehicle (DMSO), was added to GCase buffer (pH 5.4, activated with 0.2% w/v sodium taurocholate) and a GCase activity assay was performed, with a final concentration of 0.57 nM imiglucerase and 1% DMSO in the assay reaction mixture.

Lysates of H4 cells and human fibroblasts (WT/WT, L444P/L444P, and D409H/D409H) extracted in GCase buffer (pH 5.4, activated with 0.2% w/v sodium taurocholate) were used for GCase activity assays. Compounds or vehicle (DMSO) were spiked into the lysates, such that the final concentration of DMSO in the assay reaction mixture was 1%. To provide an adequate dynamic range for the activator assay, *GBA1*-WT H4, HiBiT-WT H4, and WT/WT fibroblast lysates were diluted to 0.3 µg/µL total protein, while HiBiT-N370S H4, HiBiT-L444P H4, L444P/L444P fibroblast, and D409H/D409H fibroblast lysates were diluted to 1 µg/µL total protein. For H4 cells, correction was made for compound effect in the *GBA1*-KO H4 cell background (lysates diluted to either 0.3 µg/µL or 1 µg/µL). Final protein concentration was verified by a second BCA assay, which was not used for data normalization.

Spleen tissue was collected at splenectomy from a patient with GD and *GBA1* genotype N370S/N370S, and written informed consent was obtained [26]. Samples of ~90 mg of human patient spleen tissue were each homogenized in 1.5 mL of freshly-prepared GCase buffer titrated to pH 4.7 [McIlvaine buffer (0.2 M Na_2HPO_4 and 0.1 M citric acid); cOmplete™, Mini, EDTA-free Protease Inhibitor Cocktail (Cat.#11836170001; 1 tablet/10 mL); PhosSTOP™ Phosphatase Inhibitor Cocktail Tablets (Cat.#04906837001; 1 tablet/10 mL); 0.25% (v/v) Triton X-100 (Cat.#T9284; Sigma-Aldrich)] using an Omni tissue homogenizer (Cat.#TH115; Omni International). Samples were homogenized at 75% power five times for five seconds each, briefly kept on ice, then homogenized at 50% power five times for five seconds each. Protein lysates were then sonicated twice in 10-sec pulses using a bath sonicator, the lysates were centrifuged for 20 min (10,000 x *g*; 4°C), and supernatant was collected. The spleen extracts contained 2.2 – 2.9 µg/µL of total protein. GCase activity assay was performed as described above, except that the assay buffer was titrated to pH 4.7 and was not activated with sodium taurocholate. Compounds or vehicle (DMSO) were spiked into the spleen extracts, such that the final concentration of DMSO in the assay reaction mixture was 1%. To correct for background, a subset of protein samples (10 µL/well) added to the 384-well assay plate were diluted with GCase buffer (5 µL/well) containing 0.8 mM of conduritol B epoxide (CBE; DMSO, 8% v/v), an irreversible inhibitor of GCase, and incubated for 15 min (600 rpm, 37°C). However, given that a dose-response curve describing inhibition of GCase by CBE in the spleen extracts was not generated, and that N370S GCase can display an increased IC_{50} for inhibitors like CBE [27, 28], the assumption cannot be made that GCase was completely inhibited by CBE under the experimental conditions.

Drug synergy evaluation

Synergy of drug combinations was analyzed by the SynergyFinder package [29] using the Loewe additivity model [30] to calculate synergy scores. Each drug concentration combination had three replicates which were used to fit dose-response curves. For compounds NCGC00347424 (pladienolide B), NCGC00389337 (epoxomicin), and NCGC00386288 (AZD2858), the top-three concentrations were removed from analysis, as a dose-response curve could not be fit due to toxicity at higher concentrations. A response matrix and corresponding synergy score matrix were generated for each combination. In general, negative, zero, and positive scores in the synergy matrix indicate antagonistic, additive, and synergistic interactions between drugs, respectively.

Fibroblast studies

Fibroblasts were obtained by skin punch biopsy from a healthy control (WT/WT) and three patients with GD: a male with *GBA1* genotype N370S/N370S that had type 1 GD and PD, a male with *GBA1* genotype L444P/L444P that had type 3 GD, and a female with *GBA1* genotype D409H/D409H that had type 3 GD. Fibroblast cultures were maintained in a humidified incubator (37°C, 5% CO_2) in DMEM + GlutaMAX^{TM} with high glucose (Cat.#10566-016; Gibco) supplemented with 15% heat-inactivated fetal bovine serum and 100 U/mL Penicillin-Streptomycin (Cat.#15140-122; Gibco).

For LysoFix-GBA studies, fibroblasts were seeded into 384-well PerkinElmer PhenoPlates (25,000 cells in 40 µL media) for 24 h and treated with compounds for 144 h; the LysoFix-GBA assay was then performed.

For Western blot, L444P/L444P fibroblasts were treated at confluence in a T-175 flask with vehicle (DMSO, 0.3% v/v) or pladienolide B (100 nM) for 6 days. Upon harvest, the fibroblasts were washed twice with RT PBS, incubated on ice in 15 mL of PBS for 30 min, and removed via cell-scrapping technique. The cell suspension was centrifuged at 1,500 *g* x 5 min, re-suspended in 1.5 mL of PBS, and transferred to a microcentrifuge tube. The tube was centrifuged again at 1,500 *g* x 5 min, the supernatant was discarded, and the dry pellet was stored at -80°C until protein extraction and Western blot analysis.

Preparation of N-(4-iodophenyl)-2-(2-((4-iodophenyl)amino)-2-oxoethoxy)benzamide (NCGC00241326)

(a)

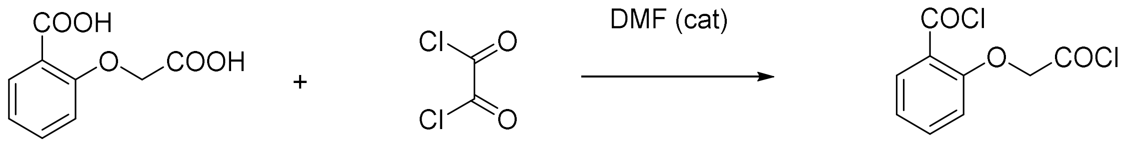

2-(carboxymethoxy)benzoic acid (0.196 g, 1 mmol) and oxalyl dichloride (0.429 mL, 5.00 mmol) in DCM (1 mL) was allowed to stir at RT. One drop of DMF (3.87 µL, 0.050 mmol) was added. The solution was stirred at RT for 30 min. The solution was concentrated. The residue was used as is for the next step.

(b)

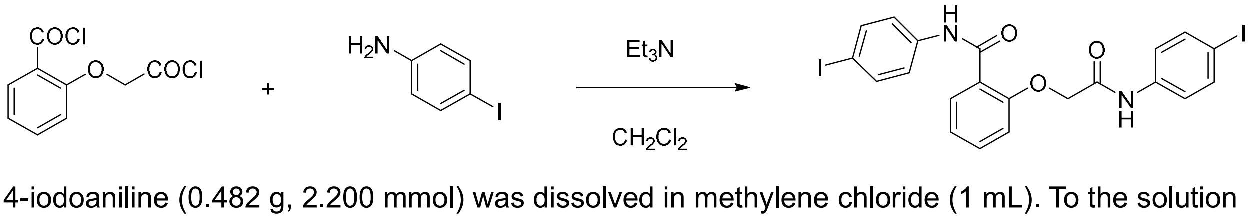

4-iodoaniline (0.482 g, 2.200 mmol) was dissolved in methylene chloride (1 mL). To the solution was added triethylamine (0.348 ml, 2.500 mmol). The solution was stirred at RT. A solution of 2-(2-chloro-2-oxoethoxy)benzoyl chloride (0.233 g, 1 mmol) in methylene chloride (1.000 mL) was added dropwise over a period of 1 min. The solution was stirred at RT for 2 hr. The solution was filtered. The solid was washed with methylene chloride. The solid was dried *in vacuo* to give the desired product, N-(4-iodophenyl)-2-(2-((4-iodophenyl)amino)-2-oxoethoxy)benzamide (117.4 mg, 20% yield). ^{1}H NMR (400 MHz, DMSO) δ 10.77 (s, 1H), 10.46 (s, 1H), 7.85 (dd, J = 7.7, 1.8 Hz, 1H), 7.77 – 7.69 (m, 6H), 7.60 (ddd, J = 8.9, 7.4, 1.8 Hz, 1H), 7.54 – 7.48 (m, 2H), 7.28 –

7.23 (m, 1H), 7.20 (td, J = 7.5, 0.9 Hz, 1H), 5.01 (s, 2H). Contains 3.5% methylene chloride. (LC-MS, ESI pos.) Calculated for $C_{21}H_{16}I_2N_2O_3$: 599.18 (M + H), Measured: 598.9.

Analytical analysis was performed on an Agilent LC/MS (Agilent Technologies, Santa Clara, CA). A 7-min gradient of 4% to 100% acetonitrile (containing 0.025% trifluoroacetic acid) in water (containing 0.05% trifluoroacetic acid) was used with an 8-min run time at a flow rate of 1.0 mL/min. A Phenomenex Luna C18 column (3 micron, 3 x 75 mm) was used at a temperature of 50°C. Purity determination was performed using an Agilent diode array detector.

Statistical analysis

Differences between groups were analyzed via either ordinary one-way ANOVA or two-way ANOVA with Tukey's or Šídák's multiple comparisons tests, or Brown-Forsythe and Welch ANOVA tests with Dunnett's T3 multiple comparisons test. Statistical analysis was performed using GraphPad Prism software (version 10.1.2).

Supplemental Figures:

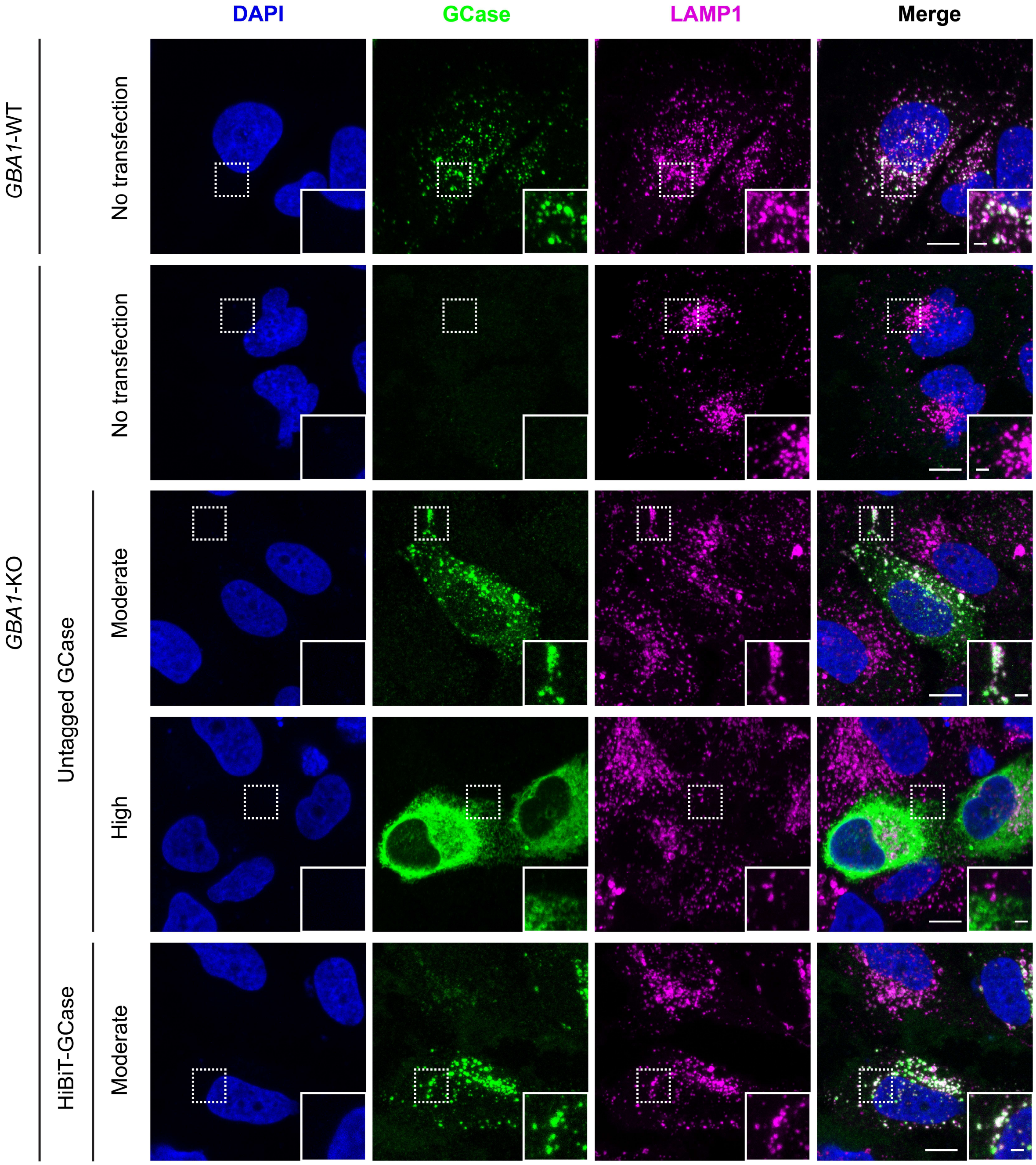

Figure S1. Overexpression of *GBA1* drives mis-localization. *GBA1*-KO H4 cells were transfected with constructs containing the HiBiT-*GBA1* transgene or untagged *GBA1*. Transfection produced a heterogenous population of cells with either low, moderate, or high levels of GCase expression; the latter perturbed GCase trafficking to the lysosome. GCase was stained with hGCase-1/23 antibody (green), while lysosomes were stained for LAMP1 (magenta). Scale bar: 10 μm; inset scale bar: 1 μm.

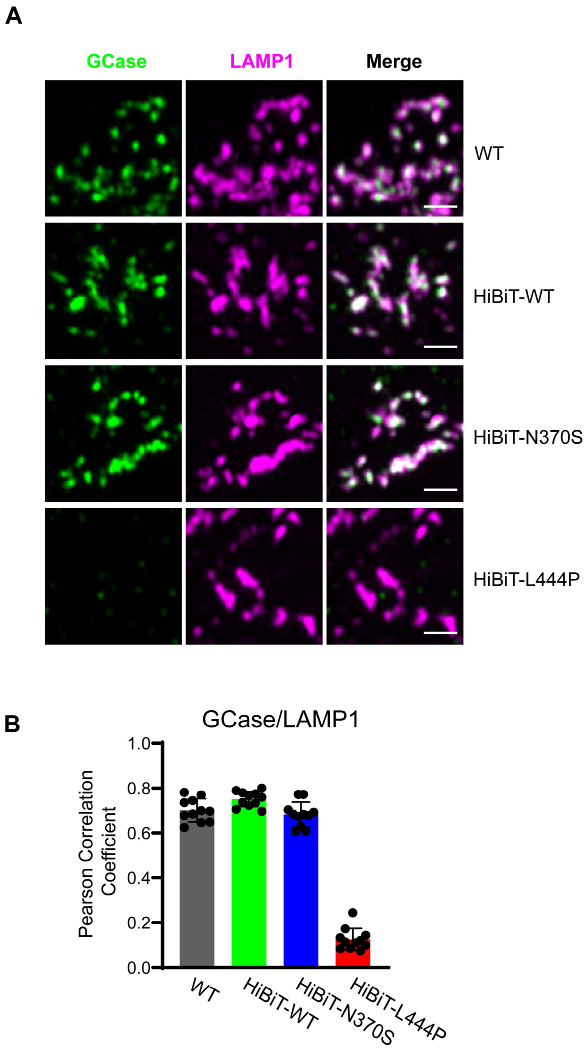

Figure S2. Colocalization of HiBiT-hGCase with LAMP1 and LIMP2. (**A, B**) *GBA1*-WT, HiBiT-GCase-WT, HiBiT-GCase-N370S, and HiBiT-GCase-L444P H4 cells were stained for GCase (green) and LAMP1 (magenta), and their colocalization was quantified using Pearson's correlation coefficient. (Error bars: SD [*n* = 11]). (**C, D**) *GBA1*-WT, HiBiT-GCase-WT, HiBiT-GCase-N370S, and HiBiT-GCase-L444P H4 cells were stained for GCase (green) and LIMP-2 (magenta), and their colocalization was quantified using Pearson's correlation coefficient. (Error bars: SD [*n* = 11-12]). Scale bar: 1 μm.

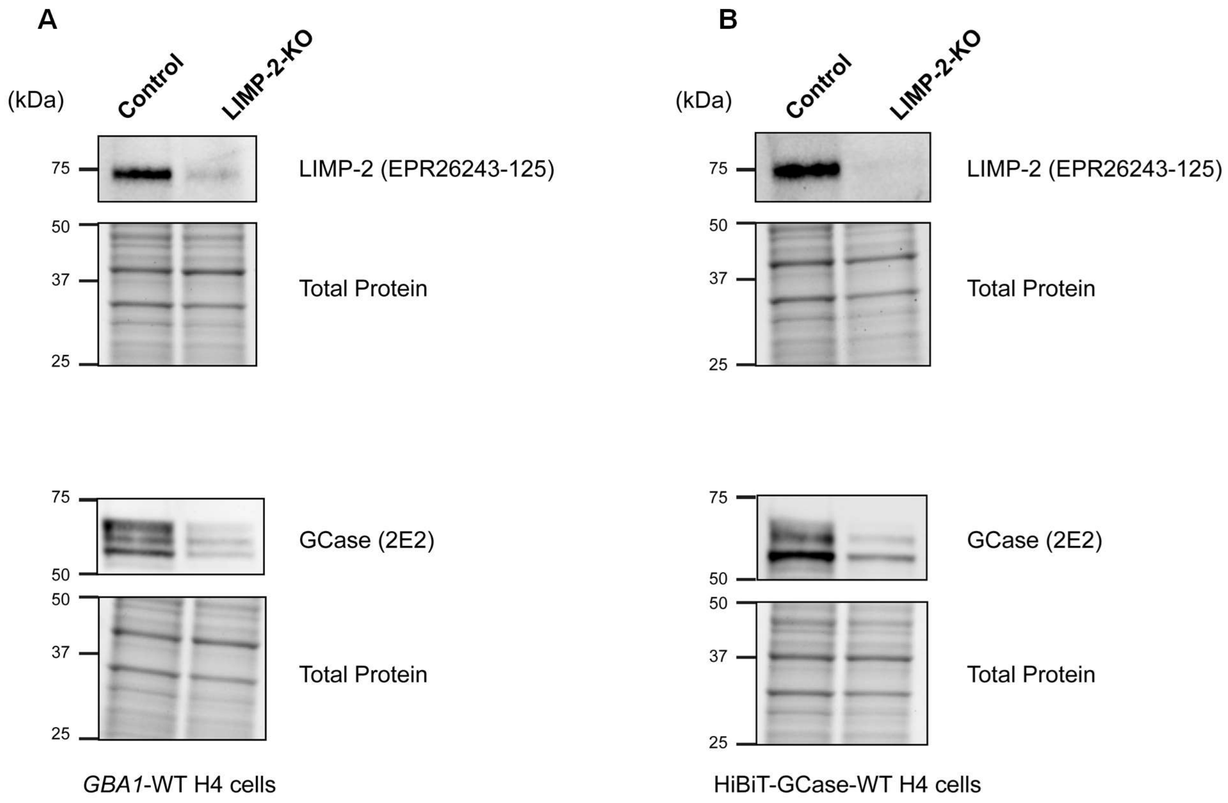

Figure S3. Lysosomal targeting of HiBiT-GCase-WT depends on LIMP-2. LIMP-2 (encoded by the *SCARB2* gene) was knocked down by transfecting pCAG-eCas9-GFP-U6-LIMP2_gRNA into (**A**) *GBA1*-WT H4 cells and (**B**) HiBiT-hGCase-WT H4 cells. Levels of LIMP-2 and GCase in FACS-enriched GFP+ *GBA1*-WT and HiBiT-hGCase-WT H4 cells were evaluated by Western blot, with total protein as the loading control. Loss of LIMP-2 impairs the delivery of GCase to lysosomes, leading to its secretion from cells and resulting in less GCase detected in cell lysates.

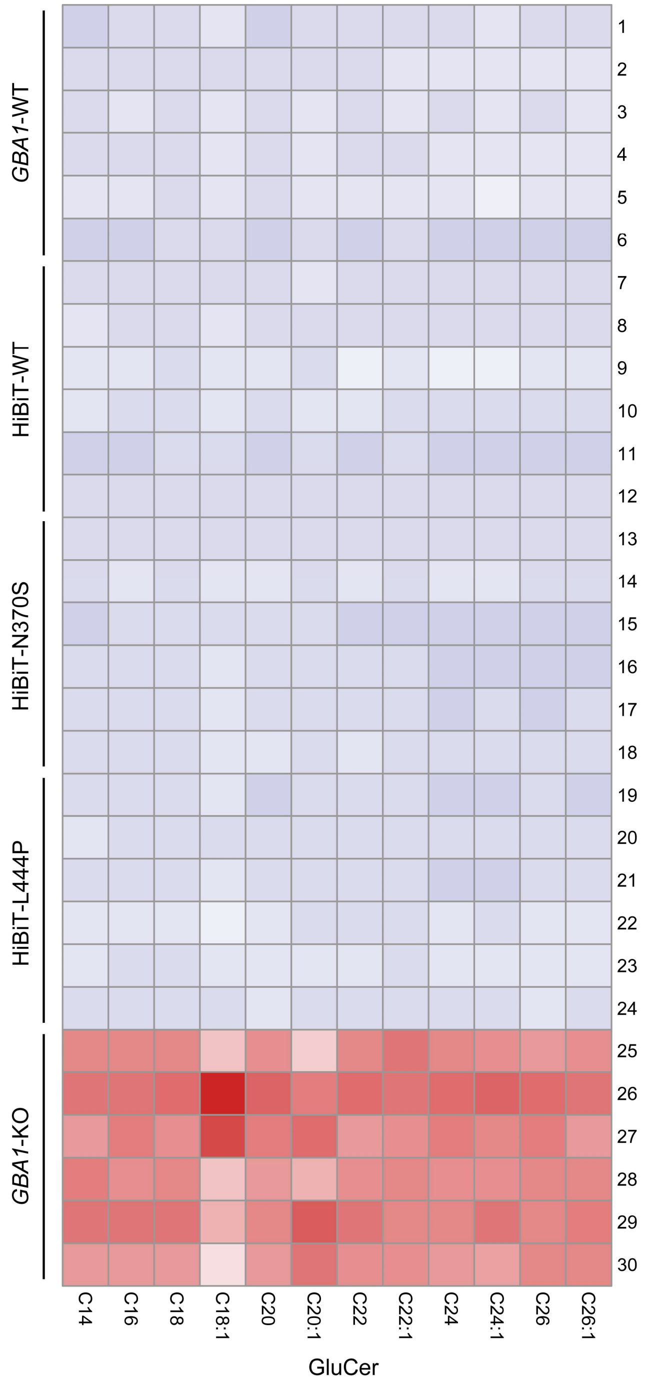

Figure S4. GluCer levels in HiBiT-GCase H4 cell lines. Levels of GluCer species with different acyl chain lengths were quantified by supercritical fluid chromatography (SFC) separation coupled with tandem mass spectrometry (MS/MS) detection (SFC-MS/MS) in *GBA1*-WT, *GBA1*-KO, and HiBiT-hGCase H4 cell lines. Each cell line is represented by six replicate samples containing 2 million cells each. Data are presented as a heatmap.

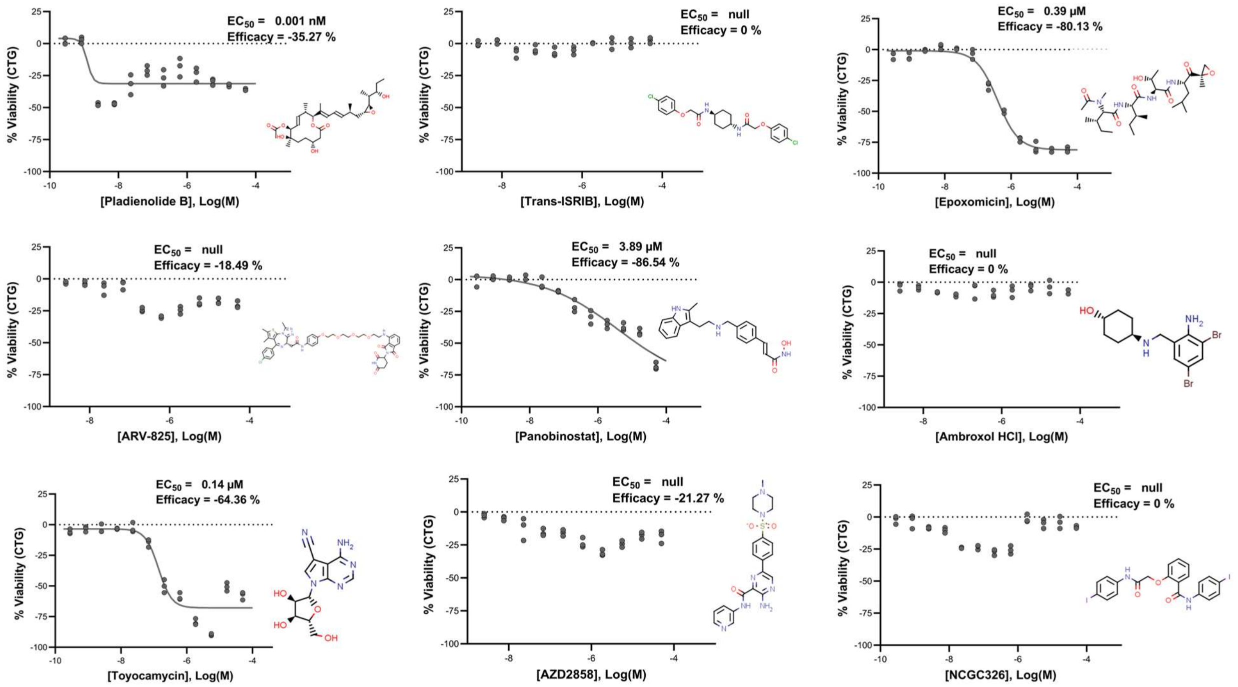

Figure S5. Follow-up cytotoxicity testing of selected primary qHTS hits using CellTiter-Glo assay. HiBiT-GCase-L444P H4 cells were seeded into 1536-well solid white plates (2,000 cells in 5 µL media) for 24 h and incubated with a titration of representative hits from the HiBiT primary screen for 24 h at concentrations ranging from 0.3 nM – 50 µM (12-point, 3x dilution series; *n* = 3). The CellTiter-Glo assay was then performed as described in the methods. Response values (% viability) are based on change in luminescence (RLU) in compound-treated versus DMSO-treated cells.

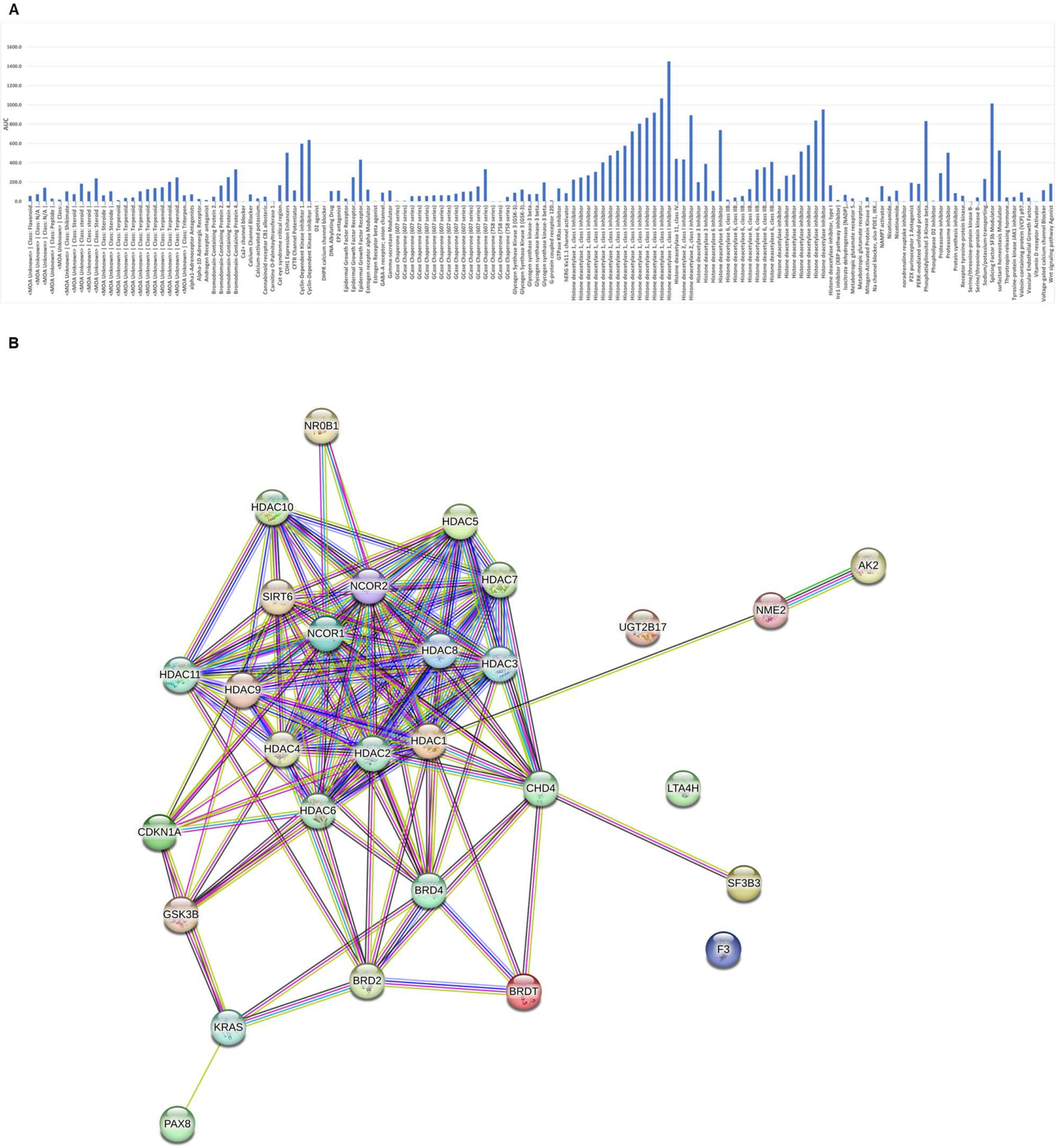

Figure S6. Primary qHTS hit mechanism of action analysis and interaction network of molecular targets. (**A**) Primary qHTS hits were sorted by mechanism of action and analyzed by their effect in the HiBiT assay using AUC. (**B**) Known and predicted protein-protein interaction network from STRING database for the 30 enriched targets identified from target profiles of final hit compounds. **Table S2** lists the pathways in the Reactome database that are overrepresented by these enriched targets, revealing potential pathways affected by the hit compounds.

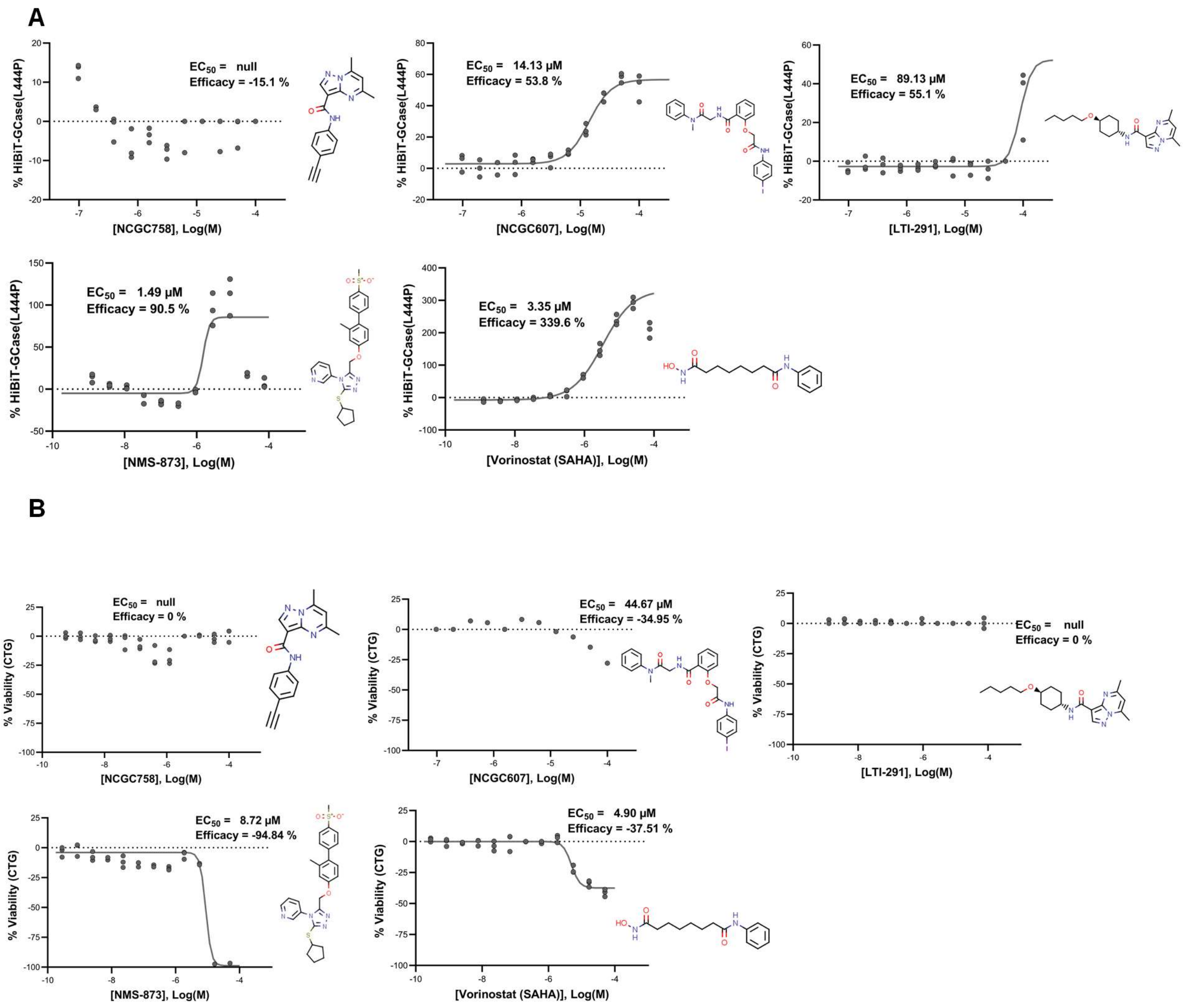

Figure S7. Primary qHTS curves and cytotoxicity testing for additional compounds. HiBiT-GCase-L444P H4 cells were seeded into 1536-well solid white plates (2,000 cells in 5 µL media) for 24 h and treated with a titration of NCGC758, NCGC607, LTI-291, NMS-873, and vorinostat (SAHA) for 24 h, after which either the HiBiT assay (**A**) or CellTiter-Glo assay (**B**) was performed as described in the methods. Data are from three independent replicates.

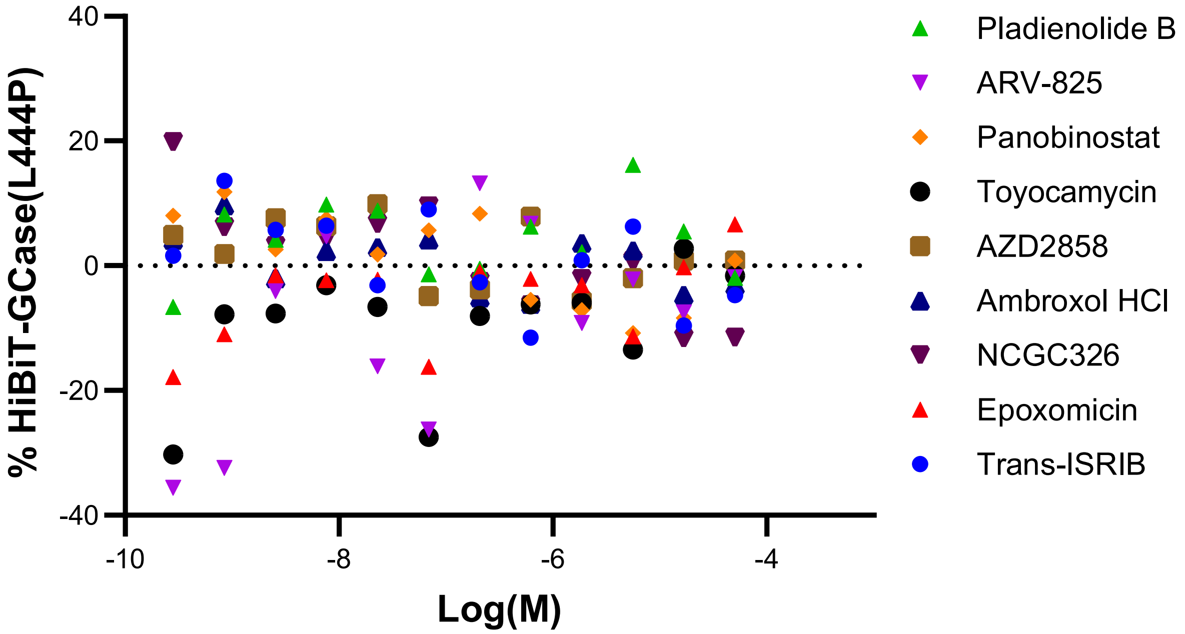

Figure S8. HiBiT-GCase-L444P assay interference testing for selected hits. Selected final hit compounds that increased HiBiT-GCase-L444P levels in H4 cells in the follow-up screen were tested for their ability to interfere with the reconstituted luciferase enzyme in the HiBiT-GCase assay. HiBiT-GCase-L444P H4 cells were seeded into 1536-well solid white plates (2,000 cells in 5 μL media) for 48 h and treated with a titration of compounds for 30 min, after which the HiBiT assay was performed to determine if the compounds were directly affecting HiBiT luminescence. Data are from three independent replicates.

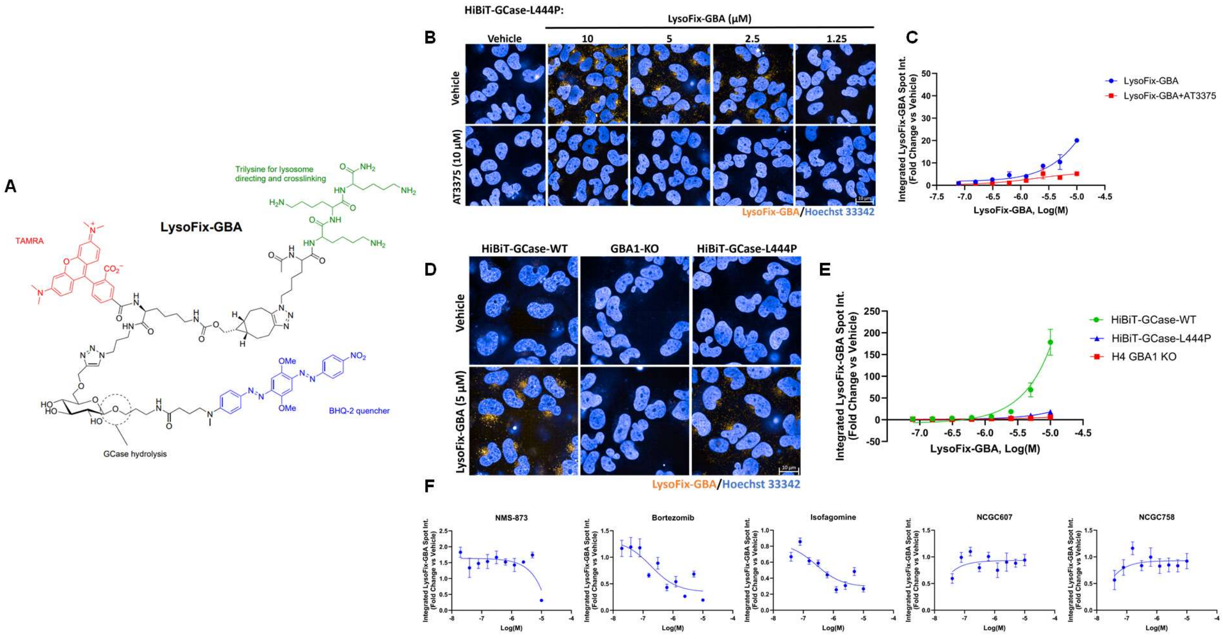

Figure S9. Implementation of LysoFix-GBA high-content screening assay in HiBiT-GCase-L444P H4 reporter line. (**A**) Chemical structure of LysoFix-GBA. (**B**) To optimize LysoFix-GBA concentration, HiBiT-GCase-L444P H4 cells were seeded into 384-well PerkinElmer PhenoPlates (25,000 cells in 40 µL media) for 24 h, followed by treatment with *GBA1* inhibitor AT3375 (10 µM) or vehicle (DMSO) for 24 h. Cells were then incubated with LysoFix-GBA (78 nM – 10 µM; 8-point, 2x dilution series) for 2 h at 37°C and imaged after 15 min of nuclear staining with Hoechst-33342 (1 µg/mL) in Fluorobrite media. Scale bar: 10 µm. (**C**) Data are represented as fold change in integrated LysoFix-GBA spot intensity per cell, relative to DMSO control. (Error bars: SEM [*n* = 4 – 6]). (**D, E**) Following the same approach, HiBiT-GCase-WT, *GBA1*-KO, and HiBiT-GCase-L444P H4 cells were tested against a titration of LysoFix-GBA. (Error bars: SEM [*n* = 3]). Scale bar: 10 µm. (**F**) HiBiT-GCase-L444P H4 cells were treated with a titration of NMS-873, bortezomib, isofagomine, chaperone NCGC607, or chaperone NCGC758 for 24 h, and *GBA1* activity was assessed by LysoFix-GBA (5 µM). (Error bars: SEM [*n* = 4 – 6]).

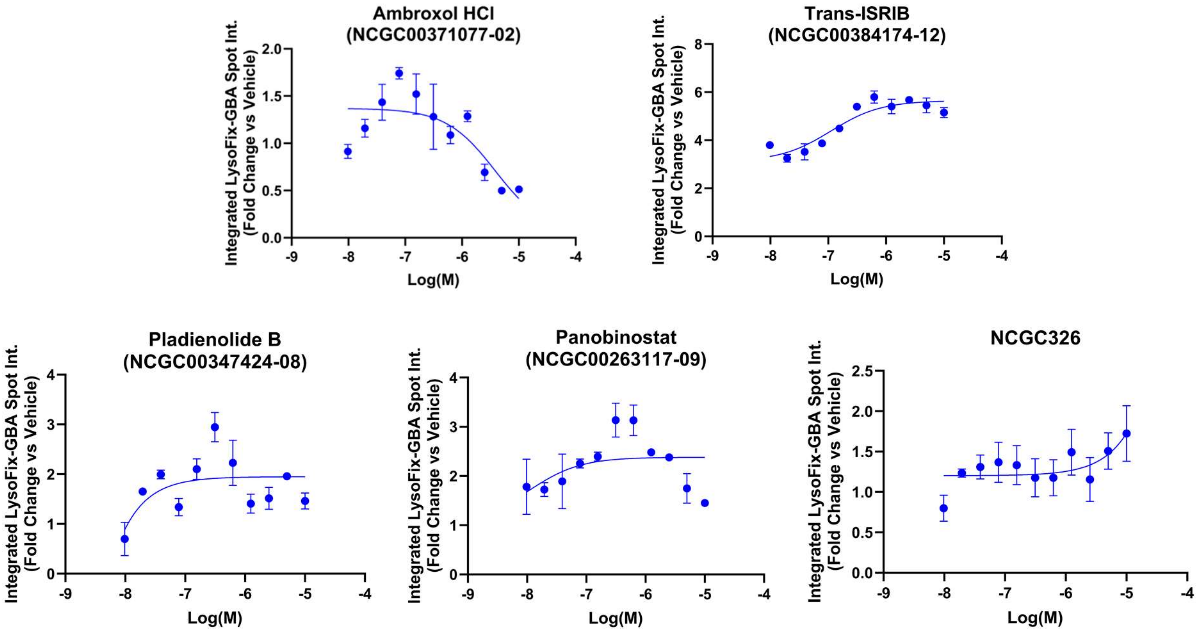

Figure S10. Evaluation of hit compounds with LysoFix-GBA assay after 72 h of compound incubation. HiBiT-GCase-L444P H4 cells were seeded into 384-well PerkinElmer PhenoPlates (25,000 cells in 40 µL media) and incubated for 24 h. Thereafter, the cells were treated with a titration of compounds (9.8 nM – 10 µM; 11-point, 2x dilution series) for 72 h and then incubated with LysoFix-GBA (5 µM) for 2 h at 37°C and 5% CO_2. High-content imaging was performed after 15 min of nuclear staining with Hoechst-33342 (1 µg/mL) in Fluorobrite media. Data are represented as the fold change (compound-treated vs. DMSO-treated) in integrated LysoFix-GBA spot intensity per cell. Dose-response curves were fit using log(agonist) vs. response (three parameters). (Error bars: SEM [*n* = 3 – 5]).

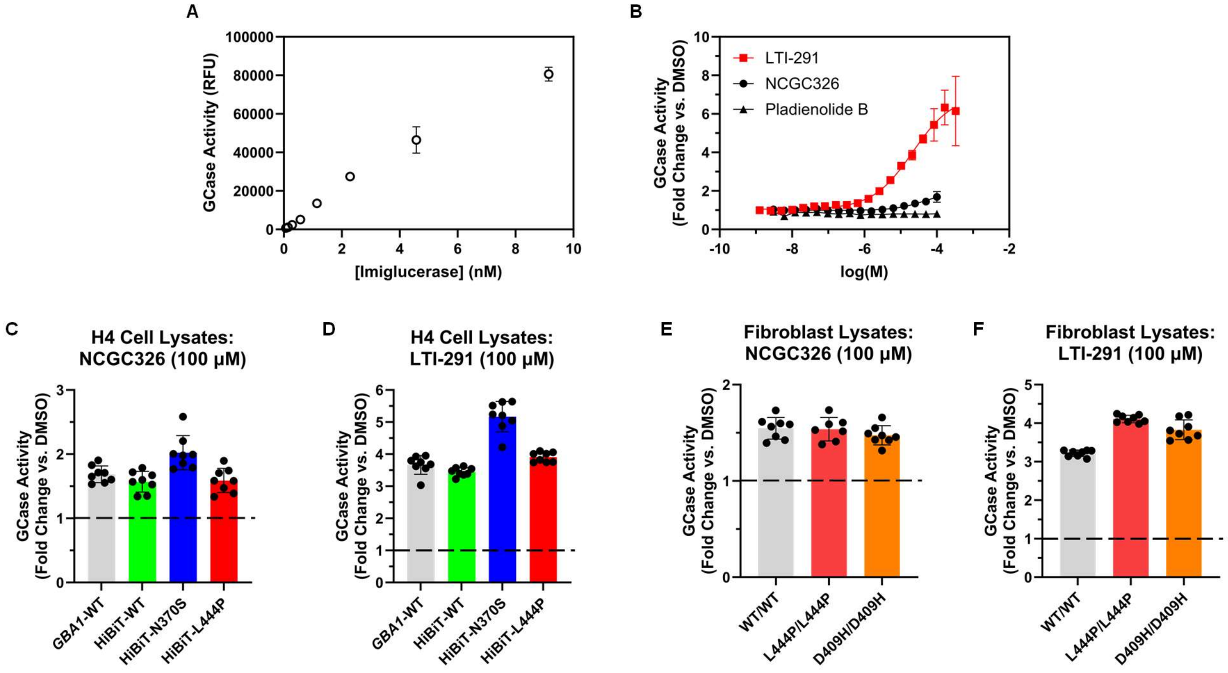

Figure S11. Evaluation of compounds in activator assay using recombinant GCase and cell lysates. (**A**) Analysis of GCase activity (cleavage of 4-MUG) versus final concentration of imiglucerase (recombinant GCase-WT) in assay reaction mixture (pH 5.4, activated with 0.2% w/v sodium taurocholate). (Error bars: SD [*n* = 7 – 8]). (**B**) Fold change in GCase activity (cleavage of 4-MUG) with compounds versus vehicle (DMSO, 1% v/v) spiked into a solution of imiglucerase in GCase buffer (pH 5.4, activated with 0.2% w/v sodium taurocholate). Pladienolide B is a negative control, as it is a proteostasis regulator that is not expected to directly bind GCase. LTI-291, a known activator of GCase that accelerates cleavage of 4-MUG, is a positive control. Dose-response data for NCGC326 and LTI-291 were fit using log(agonist) vs. response (variable slope; four parameters), while data for pladienolide B are shown with a connecting line. (Error bars: SD [*n* = 5 – 8]). Fold change in GCase activity (cleavage of 4-MUG) with (**C**) NCGC326 (100 µM) or (**D**) LTI-291 (100 µM) versus vehicle (DMSO, 1% v/v) spiked into lysates of H4 cells. Compound effect was corrected for response in *GBA1*-KO H4 cell lysates. (Error bars: SD [*n* = 8]). Fold change in GCase activity with (**E**) NCGC326 (100 µM) or (**F**) LTI-291 (100 µM) versus vehicle (DMSO, 1% v/v) spiked into lysates of human fibroblasts with *GBA1* genotypes WT/WT, L444P/L444P, and D409H/D409H. (Error bars: SD [*n* = 7 – 8]).

Gaucher Spleen Homogenate (N370S/N370S)

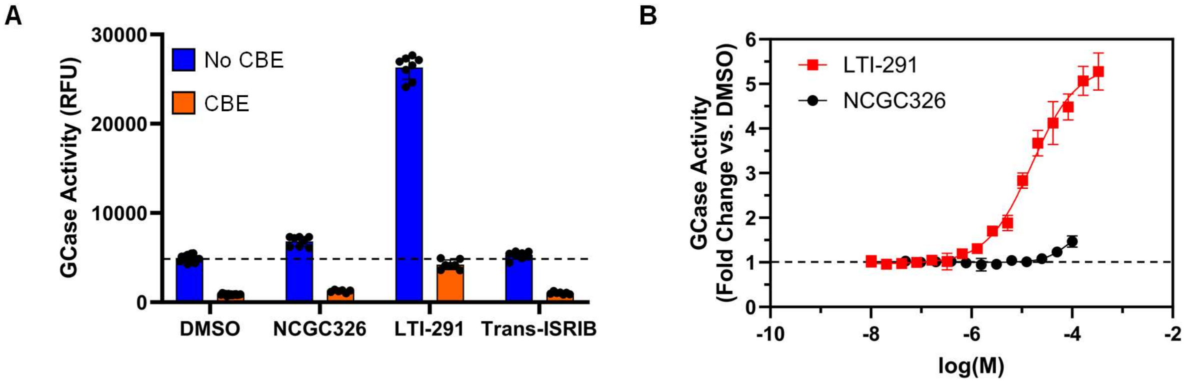

Figure S12. Evaluation of compounds in activator assay using Gaucher spleen homogenate (N370S/N370S). (**A**) GCase activity assay (cleavage of 4-MUG) for vehicle (DMSO, 1% v/v), NCGC326 (100 μM), LTI-291 (100 μM), or trans-ISRIB (50 μM) spiked into extracts of spleen tissue from a patient with Gaucher disease and *GBA1* genotype N370S/N370S buffered to pH 4.7, without sodium taurocholate activation. The final concentration of the compounds in the assay reaction mixture is the value reported. Data (relative fluorescence units, RFU) are shown with or without addition of an irreversible GCase inhibitor, conduritol B epoxide (CBE). Trans-ISRIB is a negative control, as it is a proteostasis regulator that is not expected to directly bind GCase. (Error bars: SD [DMSO: *n* = 14 – 16; NCGC326, LTI-291, trans-ISRIB: *n* = 6 – 8]). (**B**) Fold change in GCase activity (cleavage of 4-MUG) for LTI-291 or NCGC326 versus vehicle (DMSO, 1% v/v) spiked into spleen extracts from a patient with Gaucher disease and *GBA1* genotype N370S/N370S; subtraction of CBE-inhibited activity was performed. Dose-response data were fit using log(agonist) vs. response (variable slope; four parameters). (Error bars: SD [*n* = 6 – 8]).

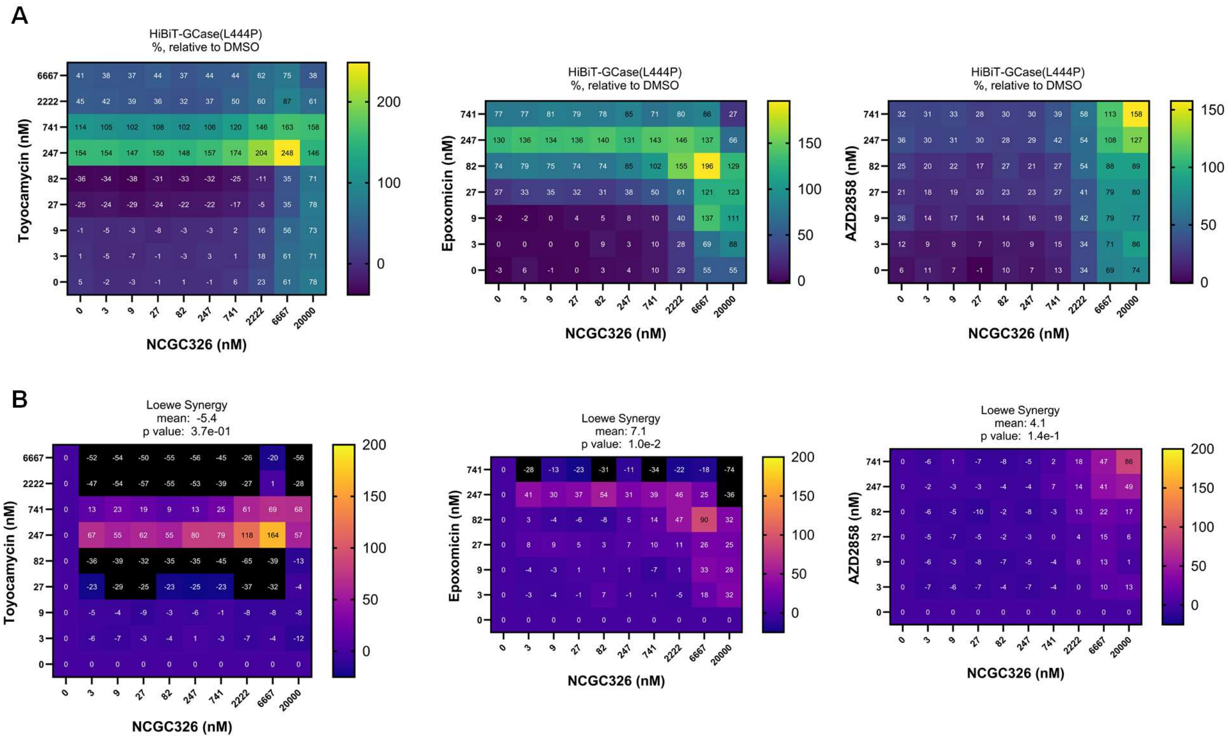

Figure S13. Matrix combination screening with NCGC326 and additional hits from primary HiBiT screen. HiBiT-GCase-L444P H4 cells were tested in 10 x 10 pairwise dose-response combinatorial matrix format. Cells were treated for 24 h with chaperone NCGC326 in a 9-point titration (3 nM – 20 µM, 3x dilution) against the same 9-point titration of toyocamycin, epoxomicin, or AZD2868; the HiBiT-GCase lytic assay was then performed. Luminescence response values were normalized to intraplate DMSO-treated controls, such that 100% activity reflects a doubling of HiBiT-GCase levels. Synergy was evaluated based on the dose-response matrix (**A**) and the Loewe synergy score (**B**). In general, negative, zero, and positive synergy scores indicate antagonistic, additive, and synergistic interactions, respectively, between drugs. If a dose-response curve could not be fit due to toxicity at top concentrations, these concentrations were omitted from the analysis. n = 3.

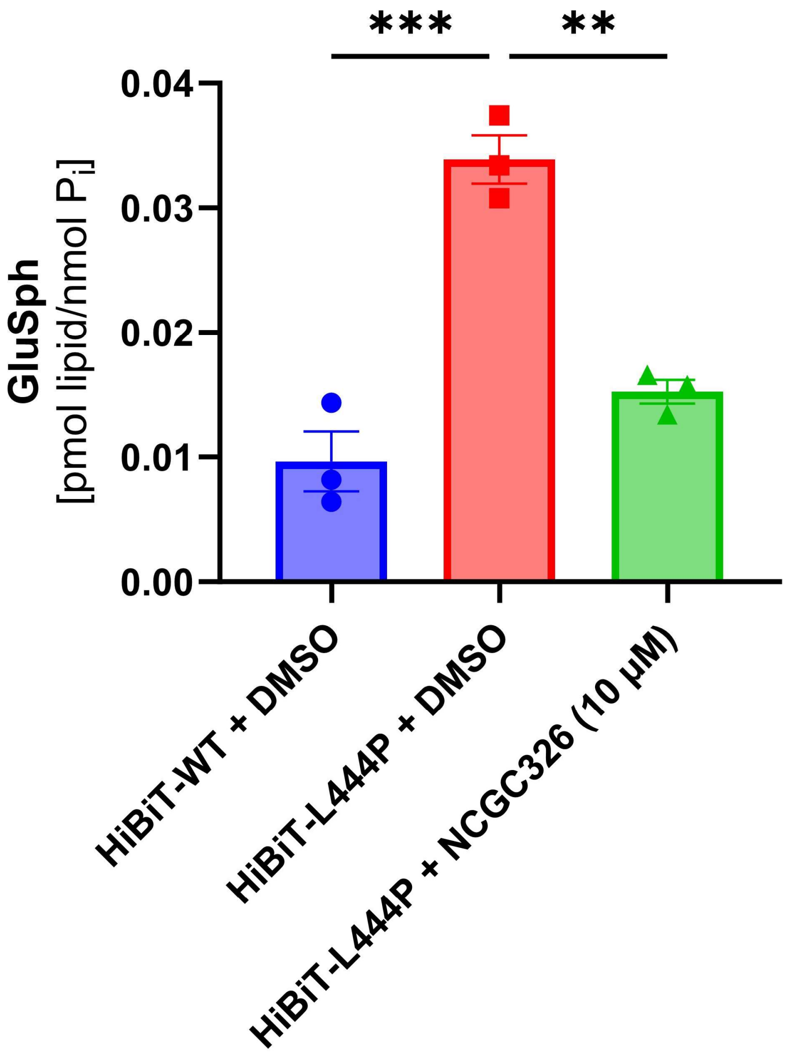

Figure S14. Treatment with NCGC326 (10 µM) reduces lipid accumulation in HiBiT-L444P H4 cells. HiBiT-GCase-WT and HiBiT-GCase-L444P H4 reporter lines were treated with vehicle (DMSO) or NCGC326 (10 µM) for 3 days. Levels of glucosylsphingosine (GluSph) were evaluated by supercritical fluid chromatography (SFC) separation coupled with tandem mass spectrometry (MS/MS) detection and normalized to total cellular inorganic phosphate (P_i) levels. (Error bars: SEM [*n* = 3]). **P ≤ 0.01, ***P ≤ 0.001.

.

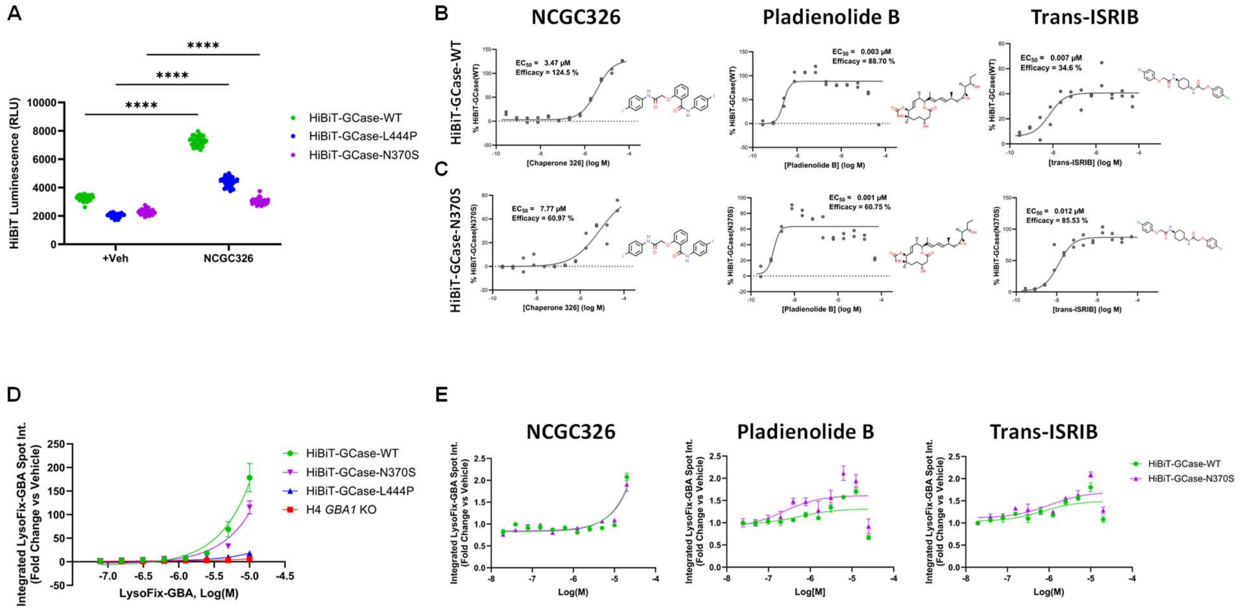

Figure S15. Evaluation of selected hits from the HiBiT-GCase-L444P screen in the HiBiT-GCase-WT and HiBiT-GCase-N370S reporter cell lines using the HiBiT assay and LysoFix-GBA assay. (**A**) H4 neuroglioma cells expressing HiBiT-GCase-WT, -N370S, or -L444P were treated with vehicle (DMSO; 0.5%) or NCGC326 (50 µM) for 24 h, and the HiBiT lytic assay was performed. Data are shown as raw luminescence (RLU). (Error bars: SEM [n = 32], two-way ANOVA with Šídák's multiple comparisons test). (**B**) HiBiT-GCase-WT or (**C**) HiBiT-GCase-N370S H4 cells were treated with a titration of NCGC326, pladienolide B, or trans-ISRIB (12-point, 3x, 50 µM top) for 24 h, and the HiBiT lytic assay was performed. [n = 2]. (**D**) HiBiT-GCase-WT, HiBiT-GCase-N370S, HiBiT-GCase-L444P, or *GBA1*-KO H4 cells were treated with a titration of LysoFix-GBA (8-point, 2x, 10 µM top) for 2 h, and the LysoFix-GBA assay was performed. (Error bars: SEM [n = 4]). (**E**) HiBiT-GCase-WT or HiBiT-GCase-N370S H4 cells were treated with a titration of NCGC326, pladienolide B, or trans-ISRIB (11-point, 2x, 20 µM top) for 24 h, and the LysoFix-GBA assay was performed. (Error bars: SEM [n = 4 – 6]).

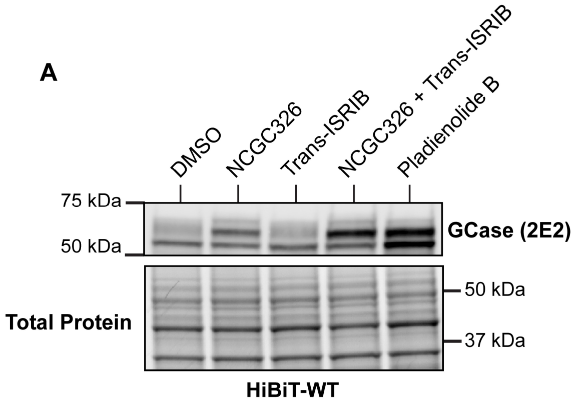

Figure S16. Western blots for HiBiT-WT and HiBiT-N370S H4 cells treated with compounds. H4 cells expressing (**A**) HiBiT-hGCase-WT and (**B**) HiBiT-hGCase-N370S were treated with vehicle (DMSO, 0.3% v/v), NCGC326 (25 μM), trans-ISRIB (1.25 μM), or the combination of NCGC326 (25 μM) and trans-ISRIB (1.25 μM) for 3 days, or with pladienolide B (100 nM) for 24 h. GCase protein levels in cell lysates were visualized on Western blot using an anti-GCase antibody (2E2, 1:2,000), with total protein as the loading control.

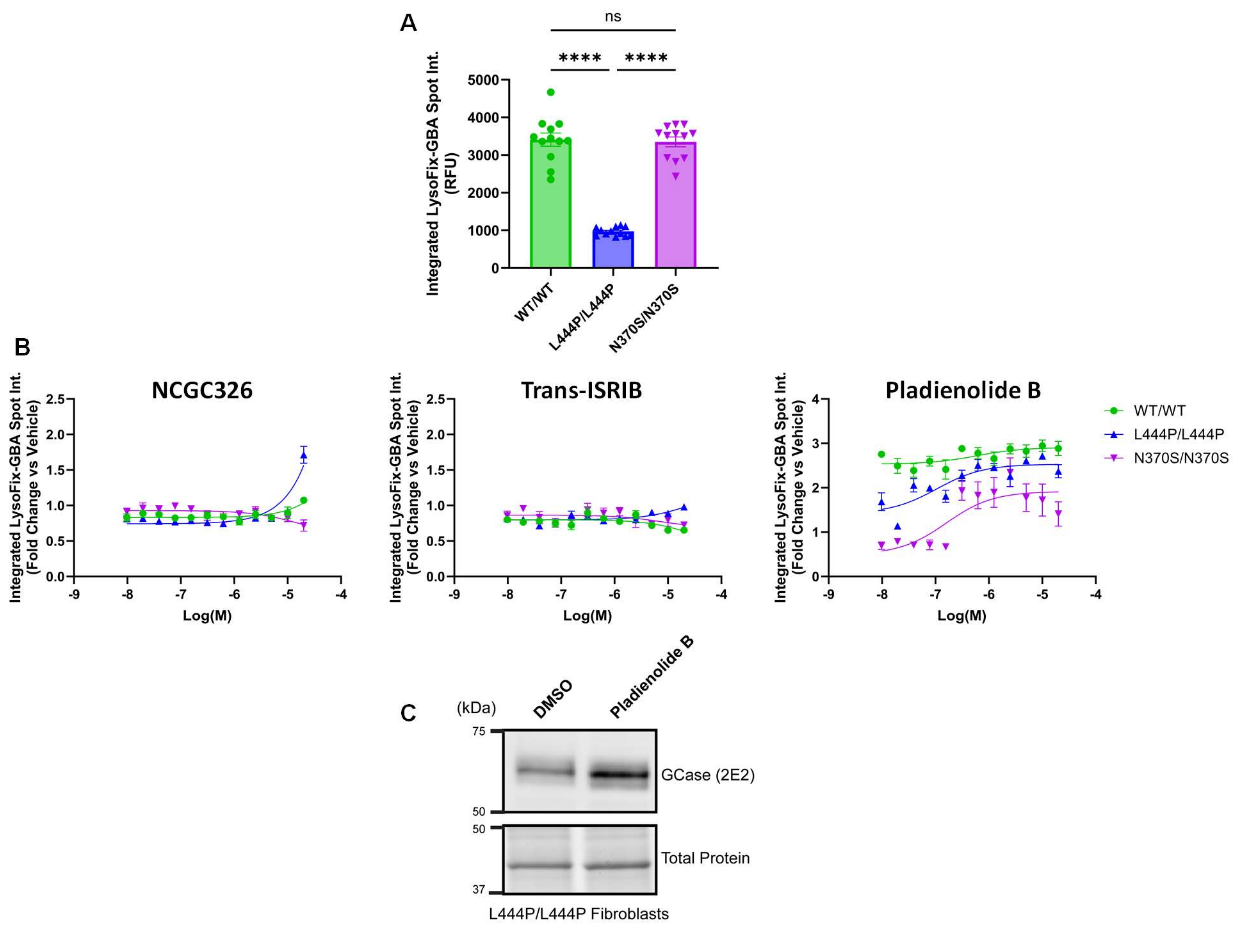

Figure S17. Evaluation of selected hits from HiBiT-GCase-L444P H4 cell-based screen in human fibroblasts. (**A**) Fibroblasts from a healthy control (WT/WT) or patients with Gaucher disease and *GBA1* genotypes L444P/L444P or N370S/N370S were seeded into 384-well PerkinElmer PhenoPlates (25,000 cells in 40 µL media) for 24 h, incubated with LysoFix-GBA (5 µM) for 2 h at 37°C, and imaged after 15 min of nuclear staining with Hoechst-33342 (1 µg/mL) in Fluorobrite media. Data are shown as integrated LysoFix-GBA spot intensity per cell, non-normalized. (Error bars: SEM [n = 12], one-way ANOVA with Šídák's multiple comparisons test). (**B**) WT/WT, L444P/L444P, or N370S/N370S fibroblasts were treated with a titration of NCGC326, trans-ISRIB, or pladienolide B (12-point, 2x, 20 µM top) for 144 h, and the LysoFix-GBA assay was performed. Data are shown as integrated LysoFix-GBA spot intensity per cell relative to DMSO control (Error bars: SEM [n = 6]). (**C**) L444P/L444P fibroblasts were treated with vehicle (DMSO, 0.3% v/v) or pladienolide B (100 nM) for 6 days, after which cell lysates were prepared, and GCase protein levels were visualized on Western blot using an anti-GCase antibody (2E2, 1:2,000), with total protein as the loading control.

References:

[1] Cerbini T, Funahashi R, Luo Y, Liu C, Park K, Rao M, et al. Transcription activator-like effector nuclease (TALEN)-mediated CLYBL targeting enables enhanced transgene expression and one-step generation of dual reporter human induced pluripotent stem cell (iPSC) and neural stem cell (NSC) lines. PLoS One. 2015;10(1):e0116032.
[2] Jong T, Gehrlein A, Sidransky E, Jagasia R, Chen Y. Characterization of Novel Human β-glucocerebrosidase Antibodies for Parkinson's Disease Research. J Parkinsons Dis. 2024;14(1):65-78.
[3] Tian R, Gachechiladze MA, Ludwig CH, Laurie MT, Hong JY, Nathaniel D, et al. CRISPR Interference-Based Platform for Multimodal Genetic Screens in Human iPSC-Derived Neurons. Neuron. 2019;104(2):239-55.e12.
[4] Cerbini T, Luo Y, Rao MS, Zou J. Transfection, selection, and colony-picking of human induced pluripotent stem cells TALEN-targeted with a GFP gene into the AAVS1 safe harbor. J Vis Exp. 2015(96).
[5] Chen Y, Tristan CA, Chen L, Jovanovic VM, Malley C, Chu PH, et al. A versatile polypharmacology platform promotes cytoprotection and viability of human pluripotent and differentiated cells. Nat Methods. 2021;18(5):528-41.
[6] Roberts B, Haupt A, Tucker A, Grancharova T, Arakaki J, Fuqua MA, et al. Systematic gene tagging using CRISPR/Cas9 in human stem cells to illuminate cell organization. Mol Biol Cell. 2017;28(21):2854-74.
[7] Cuddy LK, Mazzulli JR. Analysis of lysosomal hydrolase trafficking and activity in human iPSC-derived neuronal models. STAR Protoc. 2021;2(1):100340.
[8] Ron I, Horowitz M. ER retention and degradation as the molecular basis underlying Gaucher disease heterogeneity. Hum Mol Genet. 2005;14(16):2387-98.
[9] Mazzulli JR, Xu YH, Sun Y, Knight AL, McLean PJ, Caldwell GA, et al. Gaucher disease glucocerebrosidase and α-synuclein form a bidirectional pathogenic loop in synucleinopathies. Cell. 2011;146(1):37-52.
[10] Gehrlein A, Udayar V, Anastasi N, Morella ML, Ruf I, Brugger D, et al. Targeting neuronal lysosomal dysfunction caused by β-glucocerebrosidase deficiency with an enzyme-based brain shuttle construct. Nat Commun. 2023;14(1):2057.
[11] Bielawski J, Pierce JS, Snider J, Rembiesa B, Szulc ZM, Bielawska A. Sphingolipid analysis by high performance liquid chromatography-tandem mass spectrometry (HPLC-MS/MS). Adv Exp Med Biol. 2010;688:46-59.
[12] Fredriksen K, Aivazidis S, Sharma K, Burbidge KJ, Pitcairn C, Zunke F, et al. Pathological α-syn aggregation is mediated by glycosphingolipid chain length and the physiological state of α-syn in vivo. Proc Natl Acad Sci U S A. 2021;118(50).
[13] Bielawski J, Pierce JS, Snider J, Rembiesa B, Szulc ZM, Bielawska A. Comprehensive quantitative analysis of bioactive sphingolipids by high-performance liquid chromatography-tandem mass spectrometry. Methods Mol Biol. 2009;579:443-67.
[14] Bligh EG, Dyer WJ. A rapid method of total lipid extraction and purification. Can J Biochem Physiol. 1959;37(8):911-7.
[15] Inglese J, Auld DS, Jadhav A, Johnson RL, Simeonov A, Yasgar A, et al. Quantitative high-throughput screening: a titration-based approach that efficiently identifies biological activities in large chemical libraries. Proc Natl Acad Sci U S A. 2006;103(31):11473-8.
[16] Huang R. A Quantitative High-Throughput Screening Data Analysis Pipeline for Activity Profiling. Methods Mol Biol. 2022;2474:133-45.
[17] Mendez D, Gaulton A, Bento AP, Chambers J, De Veij M, Félix E, et al. ChEMBL: towards direct deposition of bioassay data. Nucleic Acids Res. 2019;47(D1):D930-d40.
[18] Knox C, Wilson M, Klinger CM, Franklin M, Oler E, Wilson A, et al. DrugBank 6.0: the DrugBank Knowledgebase for 2024. Nucleic Acids Res. 2024;52(D1):D1265-d75.

[19] Harding SD, Armstrong JF, Faccenda E, Southan C, Alexander SPH, Davenport AP, et al. The IUPHAR/BPS Guide to PHARMACOLOGY in 2024. Nucleic Acids Res. 2024;52(D1):D1438-d49.
[20] Whirl-Carrillo M, Huddart R, Gong L, Sangkuhl K, Thorn CF, Whaley R, Klein TE. An Evidence-Based Framework for Evaluating Pharmacogenomics Knowledge for Personalized Medicine. Clin Pharmacol Ther. 2021;110(3):563-72.
[21] Kim S, Chen J, Cheng T, Gindulyte A, He J, He S, et al. PubChem 2023 update. Nucleic Acids Res. 2023;51(D1):D1373-d80.
[22] Szklarczyk D, Franceschini A, Wyder S, Forslund K, Heller D, Huerta-Cepas J, et al. STRING v10: protein-protein interaction networks, integrated over the tree of life. Nucleic Acids Res. 2015;43(Database issue):D447-52.
[23] Milacic M, Beavers D, Conley P, Gong C, Gillespie M, Griss J, et al. The Reactome Pathway Knowledgebase 2024. Nucleic Acids Res. 2024;52(D1):D672-d8.
[24] Zhu S, Deen MC, Zhu Y, Gilormini PA, Chen X, Davis OB, et al. A Fixable Fluorescence-Quenched Substrate for Quantitation of Lysosomal Glucocerebrosidase Activity in Both Live and Fixed Cells. Angew Chem Int Ed Engl. 2023:e202309306.
[25] Urban DJ, Zheng W, Goker-Alpan O, Jadhav A, Lamarca ME, Inglese J, et al. Optimization and validation of two miniaturized glucocerebrosidase enzyme assays for high throughput screening. Comb Chem High Throughput Screen. 2008;11(10):817-24.
[26] Goldin E, Zheng W, Motabar O, Southall N, Choi JH, Marugan J, et al. High throughput screening for small molecule therapy for Gaucher disease using patient tissue as the source of mutant glucocerebrosidase. PLoS One. 2012;7(1):e29861.
[27] van Weely S, van den Berg M, Barranger JA, Sa Miranda MC, Tager JM, Aerts JM. Role of pH in determining the cell-type-specific residual activity of glucocerebrosidase in type 1 Gaucher disease. J Clin Invest. 1993;91(3):1167-75.
[28] Liou B, Kazimierczuk A, Zhang M, Scott CR, Hegde RS, Grabowski GA. Analyses of variant acid beta-glucosidases: effects of Gaucher disease mutations. J Biol Chem. 2006;281(7):4242-53.
[29] Ianevski A, Giri AK, Aittokallio T. SynergyFinder 2.0: visual analytics of multi-drug combination synergies. Nucleic Acids Res. 2020;48(W1):W488-w93.
[30] Greco WR, Bravo G, Parsons JC. The search for synergy: a critical review from a response surface perspective. Pharmacol Rev. 1995;47(2):331-85.
